# Recent Advances and Strategies in MXene-Based Electrodes for Supercapacitors: Applications, Challenges and Future Prospects

**DOI:** 10.3390/nano14010062

**Published:** 2023-12-25

**Authors:** Surya V. Prabhakar Vattikuti, Jaesool Shim, Pitcheri Rosaiah, Alain Mauger, Christian M. Julien

**Affiliations:** 1 School of Mechanical Engineering, Yeungnam University, Gyeongsan 38541, Republic of Korea; vsvprabu@gmail.com (S.V.P.V.); jshim@ynu.ac.kr (J.S.); 2 Department of Physics, Saveetha School of Engineering, Saveetha Institute of Medical and Technical Sciences (SIMATS), Thandalam, Chennai 602105, India; rosaiah.yu@gmail.com; 3 Institut de Minéralogie, de Physique des Matériaux et de Cosmologie (IMPMC), Sorbonne Université, UMR-CNRS 7590, 4 Place Jussieu, 75005 Paris, France; alain.mauger@sorbonne-universite.fr

**Keywords:** supercapacitors, MXenes, electrodes, nanostructures, wearable devices

## Abstract

With the growing demand for technologies to sustain high energy consumption, supercapacitors are gaining prominence as efficient energy storage solutions beyond conventional batteries. MXene-based electrodes have gained recognition as a promising material for supercapacitor applications because of their superior electrical conductivity, extensive surface area, and chemical stability. This review provides a comprehensive analysis of the recent progress and strategies in the development of MXene-based electrodes for supercapacitors. It covers various synthesis methods, characterization techniques, and performance parameters of these electrodes. The review also highlights the current challenges and limitations, including scalability and stability issues, and suggests potential solutions. The future outlooks and directions for further research in this field are also discussed, including the creation of new synthesis methods and the exploration of novel applications. The aim of the review is to offer a current and up-to-date understanding of the state-of-the-art in MXene-based electrodes for supercapacitors and to stimulate further research in the field.

## 1. Introduction

The discovery of graphene obtained through the mechanical exfoliation of graphite in 2004 was a major milestone in the materials industry. This discovery showed that the atomic layers of graphene have unique physical properties such as mechanical, electronic, optical, and electrical properties compared to the bulk material. As a result, this led to an increase in research on the synthesis and characterization of two-dimensional (2D) materials, which are thin crystalline solids formed layer by layer through van der Waals forces and covalent bonds. In the last two decades, a variety of 2D materials have been studied, including transition-metal oxides (TMOs) and disulfides (TMDs), borophene, silicones, phosphine, germanene, and many others. One particularly promising area of research is the use of MXene-based electrodes in supercapacitor (SC) applications [1,2,3,4].

**Figure 1:**
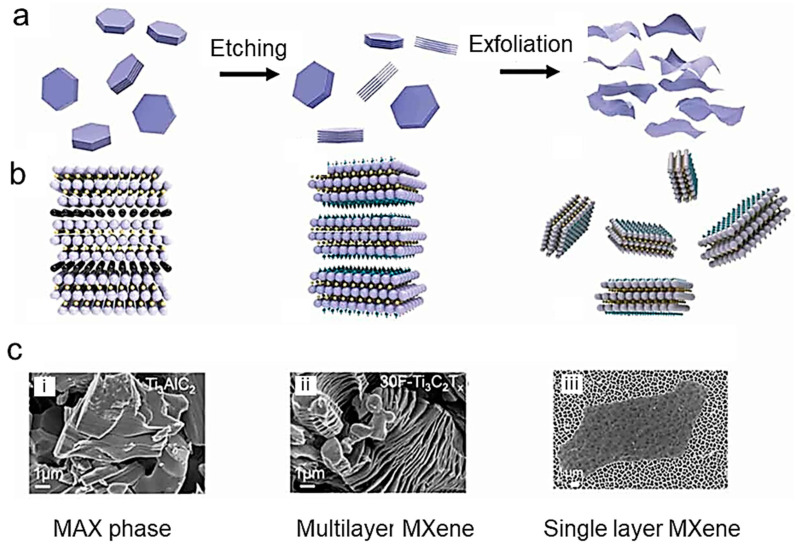
Consecutive steps of schematic steps for MXene synthesis from their MAX phase, taking Ti_3_AlC_2_ for the precursor. (**a**) Schematic diagram, an atomic structure diagram, and SEM images of the stepped MAX-phase of the precursor; (**b**) schematic diagram, an atomic structure diagram, and SEM images of accordion Ti_3_C_2_T*_x_* MXene. (**c**) Schematic diagram of MAX phase (**i**), an atomic structure diagram of multilayer MXene (**ii**), and TEM images of single-layer Ti_3_C_2_T*_x_* MXene (**iii**). Reproduced with permission from Chen and co-workers [6]. Copyright 2021 under the terms of the Creative Commons CC BY license.

MXenes, consisting of transition-metal carbides and nitrides, have become a large family of 2D materials of composition of M_n+1_X_n_T*_x_*, where M stands for a transition metal (e.g., Sc, Ti, V, Ta, Cr, Zr, Nb, Mo, Hf), X represents either carbon or nitrogen (*n* = 1, 2, or 3), and T denotes the terminal group (–F, –OH, –O, etc.) that originates from the etchant. Moreover, the physical and chemical properties of MXenes are significantly affected by the aforesaid functional groups. The first member of MXenes, Ti_3_C_2_T*_x_*, was synthesized by the Gogotsi group [5] at Drexel University in 2011 through a selective etching approach. Figure 1 shows a schematic representation of the MXene structures [6]. These electrode materials have high electrical conductivity, allowing for fast-ion transport and efficient charge storage, special hydrophilic properties, mechano-ceramic nature, and ease of processing [7]. Additionally, their unique structure with exposed transition-metal sites enables highly active electrochemical reactions. These properties make MXenes promising for various applications, including energy storage, electrical contacts for transistors, and photodetectors [8]. However, challenges such as the stability and scalability of MXene-based electrodes still need to be addressed.

The development of MXene-based nanostructures, such as nanostructured electrodes, functional device patterning, and high-quality thin films, is crucial to realize these applications [9,10,11,12]. MXenes can be produced by selectively etching the “A” elements in M_n+1_AX_n_ precursor compounds (named MAX-phases). Solution-based synthesis is a commonly used method for producing high-quality MXene powder films and structures in large quantities. MXenes are a new class of 2D materials that have received attention due to their diverse properties, such as high electrical conductivity, fast ion diffusion pathways, and tailored physical-chemical properties. Solution-based synthesis methods are popular for MXene, as they allow the scalable production of high-quality MXene structures.

The properties of MXenes determine the processing strategy used to create functional MXene-based devices, such as nanohybrid inks for inkjet printing and high-yield strength for screen printing. Large particle sizes and highly viscous inks are not suitable, as they cause nozzle-clogging problems [13]. High yield strength and pseudoplasticity are essential where high solids content is required for screen printing and extrusion printing [14]. Therefore, properties such as the structural features, concentrations, and rheological properties of MXenes determine the corresponding processing strategies that are most efficient at the lowest manufacturing cost. On the other hand, outlining the property-to-process mapping used to guide the MXene synthesis process is important for connecting MXene materials and their functional devices. Recently, MXenes have been considered one of the most competitive candidates as cathode materials for lithium-sulfur batteries due to their high electrical conductivity, strong interaction with Li polysulfides, and easy production [15]. However, there has been little attention to their electrochemical asymmetric/symmetric devices, which is the reason why we found it desirable to focus this review on the recent progress of MXene-based electrodes for supercapacitor applications and their device performance. Applications, challenges, and future prospects for MXene-based electrode processing for high-performance supercapacitor devices are discussed in Section 11.

## 2. Synthesis of MXenes

The development of functional MXene devices requires a good understanding of the synthesis methods and the properties of MXenes. Synthesis methods are based on chemical and/or mechanical exfoliation of the bulk crystal of M_n+1_AX_n_, in which “M” denotes a *d*-block transition metal, “A” is an IIIA or IVA element, i.e., Si, Al, Ge or Sn, and “X” represents carbon or nitrogen with *n* = 1, 2, or 3 (so-called MAX-phase). The layers “M” and “A” are intercalated in the MAX-phases forming a hexagonal lattice (*P*6_3_/*mmc* space group). Researchers have used various synthesis strategies to produce high-quality MXene-based electrodes, including solution-based synthesis [16,17], electrochemical etching [18], deposition techniques [19], 3D printing methods [20], hydrothermal methods [21], and templating methods [22,23]. For a discussion around top-down synthetic methods and challenges in scalable MXene manufacturing, see the recent review by Lim et al. [24]. Computational modeling and in situ and ex situ characterization data have been examined to rationalize the reactivity and selectivity of MXenes towards various common etching and delamination methods. The specific properties of MXenes result from several factors: effects of MAX phase, predominant precursor, and non-MAX layered materials.

Studies have shown that incorporating MXenes into supercapacitor electrodes can significantly improve specific capacitance and energy density compared to conventional materials [25,26]. A two-step process was performed to prepare exfoliated Ti_3_C_2_T*_x_*, including etched aluminum from the Ti_3_AlC_2_ MAX-phase using concentrated hydrofluoric acid and then intercalated multilayered sheets with organic molecules (hydrazine monohydrate). This intercalation reaction increases the *c*-lattice parameter from 19.5 to 26.8 Å. In another work, Gogotsi’s group also reported a more effective synthetic method employing the mixture of lithium fluoride (LiF) and hydrochloride (HCl) as the etching agent to cleave the “M-A” bond in the MAX precursor. Herein, HF was formed in situ with lithium ions intercalated during the etching process, resulting in Ti_3_C_2_T*_x_* “clay” [27]. In all cases, the exfoliation process of the Ti_3_AlC_2_ MAX-phases and the formation of MXenes can be described using the following reactions [28]:

Ti_3_AlC_2_ + 3HF → AlF_3_ + Ti_3_C_2_ + 1.5H_2_, (1)

Ti_3_C_2_ + 2H_2_O → Ti_3_C_2_(OH)_2_ + H_2_, (2)

Ti_3_C_2_ + 2HF → Ti_3_C_2_F_2_ + H_2_. (3)

Sang et al. developed an improved etching route to synthesize Ti_3_C_2_T*_x_* MXenes using a minimally intensive layer delamination (MILD) method starting from Ti_3_AlC_2_ powder, which eliminates the need for sonication and produces large Ti_3_C_2_T*_x_* flakes [29]. The etchant solution was prepared by dissolving 1 g of LiF in 20 mL of 6 mol L^−1^ HCl in a 100 mL polypropylene plastic vial. The reaction was allowed to proceed for 24 h at 35 °C. The acidic product was washed copiously with deionized (DI) water via centrifuging at 3500× *g* rpm until pH ≥ 6. At this stage, a dark green supernatant solution of large Ti_3_C_2_T*_x_* flakes was collected after 1 h of centrifuging at the same rpm. Up to 1.5 mg mL^−1^ of Ti_3_C_2_T*_x_* colloidal solution was collected. The conductivity of the single-layer Ti_3_C_2_T*_x_* thus obtained is ≈6.76 × 10^5^ S m^−1^. Recently, El-Ghazaly and co-workers [30] demonstrated a one-step synthesis method with local Ti_3_AlC_2_ MAX to Ti_3_C_2_T_z_ MXene conversion in milliseconds, facilitated by proton production through LiF solution dissociation under megahertz frequency acoustic excitation.

Several attempts have been made to replace the corrosive HF with bifluorides like KHF_2_ and NH_4_HF_2_ as the etching agent operating at 60 °C with enlargement of the interplanar space of Ti_3_C_2_ with either K^+^ or NH_4_^+^ cations [31]. Lipatov et al. [32] used LiF and HF as the etchant without sonication and adjusted the molar ratio of LiF to Ti_3_AlC_2_ to 7.5:1 to obtain 1.5 nm thick monolayer Ti_3_C_2_T*_x_* flakes. Wang et al. [33] proposed a facile hydrothermal route for the synthesis of Ti_3_C_2_T*_x_* using NH_4_F at 150 °C, in which NH_4_F is gradually hydrolyzed to generate HF as the etch agent. Wang et al. [34] used FeF_3_ instead of LiF to fabricate 2D Ti_3_C_2_ MXene, resulting in significant differences in terms of surface functionalization (including the insertion of iron cations between the MXene sheets), morphology, nature of impurities, water intercalation and reactivity in comparison with samples prepared using conventional etching methods. Peng et al. [35] developed a new approach to synthesizing 2D Ti_3_C_2_T*_x_* MXenes via a solvothermal treatment using the Ti_3_AlC_2_ MAX phase. The powders were made using the hot-press method and treated in a mixture of sodium tetrafluoroborate (NaBF_4_) and hydrochloric acid. In a typical synthesis, 0.75 g NaBF_4_ was dissolved in 15 mL 37 wt.% HCl, then 0.25 g Ti_3_AlC_2_ was added and stirred to mix uniformly. The suspension was transferred to a 100-mL autoclave and treated at 180 °C for 8–32 h to obtain Ti_3_C_2_. The MXene flakes were further prepared by sonication-assisted de-lamination using 0.5 g MXenes added to 10 mL dimethyl sulfoxide (DMSO). From the above-mentioned literature, it appears that the removal of Al in the atomic layer of the Ti_3_AlC_2_ depends mainly on the etch ability of HF, and the increasing *c*-parameter value relies on the radii of intercalated species.

Guo et al. [36] found that Ti_3_C_2_T*_x_* with –F surface termination negatively impacts its electrochemical performance as an electrode in supercapacitors. Therefore, the replacement of –F with –O surface terminations is critical and requires effective surface treatment. These authors replaced –F with –O surface terminations through a LiCl-KCl-K_2_CO_3_ molten salt treatment at atmospheric pressure and introduced potassium intercalation. This led to an increased –O content from 0.79 to 24.18 at.% and a decreased –F content from 11.23 to 3.43 at.%. The functionalized electrode showed a higher specific capacitance of 323.6 F g^−1^ at 1 A g^−1^ in 1 mol L^−1^ H_2_SO_4_ and excellent capacitance retention of 97% after 10,000 cycles at 10 A g^−1^. The mechanism of storage was attributed to the reversible transformation of Ti_3_C_2_O_2_/Ti_3_C_2_(OH)_2_ through the intercalation/extraction of hydronium (H^+^), leading to an increase in conductivity and in the electrochemically active surface area. Fu et al. [37] reported a method to synthesize Ti_3_C_2_T*_x_* for use as an electrode in supercapacitors using cetyltrimethylammonium bromide (CTAB) surfactant and HF as an etchant to exfoliate the material and increase the interlayer spacing. The resulting e-Ti_3_C_2_T*_x_* nanosheets had an interlayer spacing of 3.78 nm and exhibited a specific capacitance of 322 F g^−1^ at 5 mV s^−1^ in 1 mol L^−1^ H_2_SO_4_ electrolyte with 60% capacitance retention after 10,000 cycles. The authors found that the expanded interlayer spacing allows for improved electrochemical behavior and energy storage activity, especially in neutral and alkaline electrolytes. DFT studies confirmed that optimal energy storage activity can only be achieved with optimal interlayer spacing. Zhang et al. [38] reported a method for synthesizing Ti_3_C_2_@CuCl composite for use as electrodes in supercapacitors using different concentrations of CuCl_2_ solutions as a fluorine-free etchant. The method was performed at room temperature and was able to etch the MAX-phase in one step without the need for post-treatment. The use of 12 wt.% CuCl in 6 mol L^−1^ KOH increased the specific capacitance to 509 F g^−1^ at 1 A g^−1^, which is much higher than that of bare Ti_3_C_2_. Shen et al. [39] developed a one-pot green process for synthesizing MXene (Ti_3_C_2_Cl_2_) using a molten salt-assisted electrochemical etching. This process uses electrons as reactants to separate cathodic reduction and anodic etching, leading to a purer form of Ti_3_C_2_Cl_2_. By adding different inorganic salts, the surface terminals can be modified in situ, which shortens the modification steps and results in different surface terminations. This process is also environmentally friendly as no acidic waste is generated, and the used salts can be recycled. Wen et al. [40] demonstrated that vertically oriented Ti_3_C_2_T*_x_* and reduced graphene oxide (rGO) electrodes prepared from electrochemical co-deposition can be used as high-frequency AC filtering pseudocapacitors. The 3D vertical structure of the electrodes is considered being ideal, as it has a short ion-transport path and a fully exposed surface. The combination of electronic conductivity and large pseudocapacitive properties of Ti_3_C_2_T*_x_* results in an areal capacitance of 1.14 mF cm^−2^ with a phase angle of −80° at 120 Hz, which is twice those of others reported in MXene-based filter capacitors and exceeds most of electric double layer capacitors with a similar phase angle. The device consisting of PEDOT as the positive electrode and v-Ti_3_C_2_T*_x_*/rGO as the negative electrode achieved an energy density (ED) of 805 μF V^2^ cm^−2^ at 120 Hz. When used as a portable wind power generator, it was able to provide a reliable and stable DC signal, even when wind speed changes, making it a promising filter capacitor for miniaturization applications.

**Figure 2:**
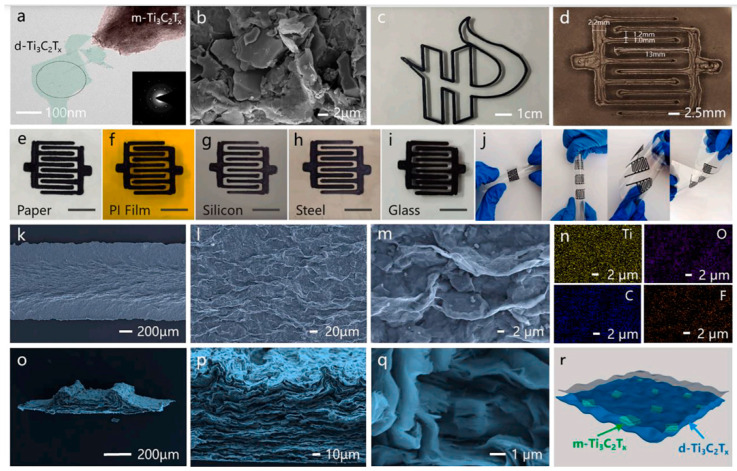
(**a**) TEM and SAED images of the MXene deposited ink. (**b**) SEM picture of the MXene sediment showing the presence of multi-layer MXene. (**c**) 3D printed school logos. (**d**) Optical photographs of the electrode. The MXene sediment inks printing on various substrates such as (**e**) paper, (**f**) PI film, (**g**) silicon wafer, (**h**) stainless steel plate, and (**i**) glass. Scale bars in Figure (**e**) to *i* are 1 cm. (**j**) The printed devices showed excellent adhesion to the substrate during repeated bending and twisting. (**k**–**m**,**o**–**q**) SEM images of the surface and cross-section of the prepared electrode at different magnifications. (**n**) EDS analysis of electrode surfaces corresponding to Ti, O, C, and F corresponding elemental maps. (**r**) Schematic diagram of the microstructure of the electrode. Reproduced with permission from [44]. Copyright 2023 Elsevier.

A mechanistic method was developed by Chen et al. [41] for creating nanoscale layered electrode structures made of Ti_3_C_2_ using a highly viscous reaction medium of 1-butyl-3-methylimidazolium chloride ([C_4_mim]Cl) ionic liquid, MAX-phase as a precursor, and NH_4_HF_2_ as an etchant. The formation of hydrogen bubbles in the interlayer space exfoliates the Ti_3_C_2_ powder and results in flexible worm-like morphologies of thin MXene stacks. A binder-free electrode with a compressive stress of 300 MPa on expanded Ti_3_C_2_ and a porosity of 28.2 was produced, providing an areal capacitance of 11.4 F cm^−2^ and a gravimetric capacitance of 304 F g^−1^ for an electrode with a thickness of 150 μm. Ai et al. [42] developed a high-yield synthesis process to produce two-dimensional V_2_C MXene, which was obtained by etching aluminum in a NaF/HCl solution under hydrothermal conditions. The resulting V_2_C electrode showed a high capacitance of 556.7 F g^−1^ at 2 mV s^−1^ in 1 mol L^−1^ Na_2_SO_4_, surpassing the capacitance of Ti_3_C_2_T*_x_* (100 F g^−1^). With a capacitance of 223.5 F g^−1^ at 100 mA g^−1^ and high conductivity, the V_2_C electrode offered good stability and retained over 5000 cycles, making it a promising option for fabricating high-capacity and stable MXene-based memory devices. Kim et al. [43] developed a flexible micro-supercapacitor (MSC) using photolithographic and solution processes. The process involves the fabrication of interdigitated micropatterns of MXene and 3D interconnected nanoporous MXene electrodes, which were transferred onto flexible substrates using a selective etching method. The 3D nanoporous interconnected MXenes were made by forming nanopores on MXene nanosheets in a 900 °C reduced atmosphere, which facilitated the formation of nanochannels in the vertical direction. This flexible MSC showed a volume capacitance of 1.727 F cm^−3^, an ED of 42 mWh cm^−3^, and a PD of 1.2 W cm^−3^, with a 140% increase in volume capacitance after 10,000 cycles. The process was scalable and allowed for the fabrication of 107 chips in an 8-inch wafer. Yuan et al. [44] proposed a new method for fabricating MSCs using 3D printing. The method uses an MXene aqueous precipitation ink to print MSCs with various structures directly on a substrate. The authors found that the 3D-printed MSCs had a high storage capacity, with a maximum areal capacitance of 2.38 F cm^−2^ and an ED of 207.8 μWh cm^−2^. They also found that the capacitance was maintained at 93.1% after testing. The results suggest that MXene deposition inks have potential for use in next-generation 3D printing of high-capacity energy-density devices (see Figure 2).

Kong et al. obtained a remarkable result with Ti_3_C_2_T*_x_* only [45]. The result was obtained with a 3D porous MXene foam using natural rubber as a template. The rubber particles not only created the pores but also prevented the stacking of the Ti_3_C_2_T*_x_* flakes. As a result, the electrode exhibited a capacitance of 480 F g^−1^ at 2 mV s^−1^ and a superior capacitance retention of 42.1% at 1000 mV s^−1^. The rubber is inexpensive, and this result provides an alternate route to produce foam electrodes on a large scale for portable and integrated supercapacitors. CdS@Nb_2_O_5_/Nb_2_CT*_x_* MXene heterojunction with hierarchical structure was synthesized via three steps. First, Nb_2_CT*_x_* MXene was prepared by hydrofluoric acid etching. The Nb_2_O_5_/Nb_2_CT*_x_* was prepared using a hydrothermal method, and finally, the CdS@Nb_2_O_5_/Nb_2_CT*_x_* was obtained by dispersion in Cd(CH_3_COO)_2_ solution using (3-aminopropyl)triethoxysilane (APTES) as a coupling agent for fixing Cd^2+^ under sonication [46].

For energy storage devices, one of the important factors to be considered for the synthesis is the synergy between the hybrid materials. For instance, 3D interconnected networks of 1T-MoS_2_/Ti_3_C_2_ MXene heterostructures prepared using magneto-hydrothermal synthesis provided enhancement in the electrochemical properties in supercapacitor applications [47]. Nb_2_CT*_x_* electrode was directly prepared with excellent lithium-ion storage capacity using a simple method of treating Nb_2_AlC with a mixed solution of HCl and LiF with large interlayer spacing, good surface group configuration, and pre-intercalated Li^+^. The Li-Nb_2_CT*_x_* was synthesized by etching Nb_2_AlC in a mixed solution of HCl (37% conc, 20 mL) and LiF (2.0 g) (closed condition, 60 °C, 90 h). Then, the obtained precipitates were washed 4 times with 1 mol L^−1^ HCl and deionized water, respectively (centrifuged at 8000 rpm). Finally, the precipitates were vacuum-dried overnight at 60 °C to obtain Li-Nb_2_CT*_x_* powders [48].

## 3. The Different MXene Phases

Numerous MXene phases have been reported, including Ti_3_C_2_T*_x_* [49], Ti_2_CT*_x_* [50], Nb_2_CT*_x_* [51], Nb_4_C_3_T*_x_* [52], Mo_2_C [53], V_2_CT*_x_* [54], among others. Of these phases, Ti_3_C_2_T*_x_* is commonly studied due to its superior intercalation pseudocapacitance behavior and electronic conductivity [55]. However, its large molecular mass and multiple atomic layers per formula unit limit its electrochemical performance.

### 3.1. Nb_2_CT_x_

Nb_2_CT*_x_* is one of the few atomic layer configuration materials among MXenes, and it is also considered a promising electrode material for energy storage [48]. For instance, Nb_2_CT*_x_* Li-ion supercapacitors were developed [56], and the electrochemical performance of the layered Nb_2_CT*_x_*/CNT composite electrodes was found to be superior to that of layered Nb_2_CT*_x_* [57]. However, Nb_2_CT*_x_* displays an electronic conductivity of 24 S cm^−1^, which is two orders of magnitude lower than that of the Ti_3_C_2_T*_x_* film (10^3^ S cm^−1^) [58,59]. This might be one of the factors hindering its application in aqueous supercapacitors. For instance, Lin et al. demonstrated that Nb_2_CT*_x_* can be used for photothermal tumor eradication in NIR-I and NIR-II bio-windows due to its biodegradable nature [51]. Zhang et al. showed that Nb_2_CT*_x_* Li-ion capacitors were superior in electrochemical performance compared to layered Nb_2_CT*_x_* [56]. However, the low conductivity can be improved by adding carbon nanotubes as a conductive agent, as shown by Xiao et al. [60]. The authors proposed a chemical etching method to synthesize highly crystalline Nb_2_CT*_x_* and used it in a Nb_2_CT*_x_*/CNT negative electrode/activated carbon positive electrode cell, which showed an ED of 154.1 μWh cm^−2^ at a PD of 74,843.1 μW cm^−2^ with a mass loading of 10 mg. This suggests that Nb_2_CT*_x_* has potential for use in high-performance asymmetric supercapacitor applications.

**Figure 3:**
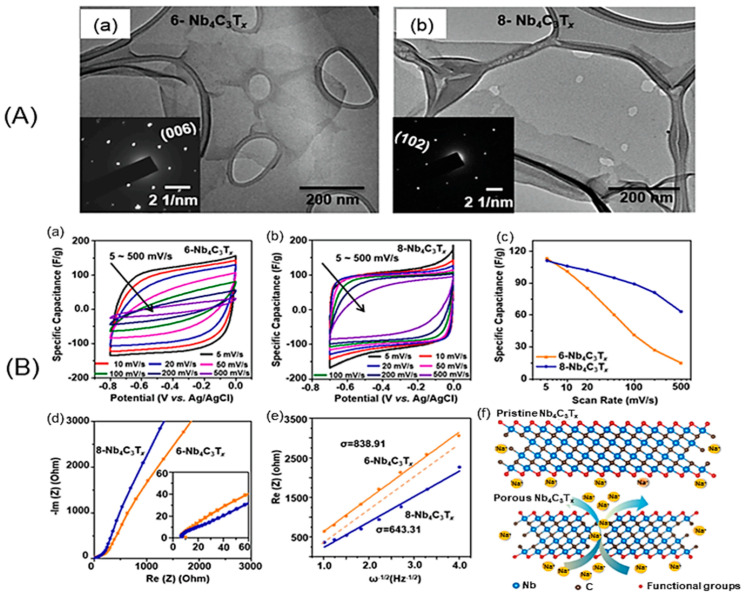
(**A**) TEM images of (**a**) 6-Nb_4_C_3_T*_x_* and (**b**) 8-Nb_4_C_3_T*_x_* flakes. The inset shows the SAED patterns of respective crystalline single-layer flakes. (**B**) Electrochemical performance of Nb_4_C_3_T*_x_* films in 1 mol L^−1^ Na_2_SO_4_ electrolyte. Cyclic voltammograms (CVs) of (**a**) 6-Nb_4_C_3_T*_x_* and (**b**) 8-Nb_4_C_3_T*_x_* at scan rates from 5 to 500 mV s^−1^ in 1 mol L^−1^ Na_2_SO_4_ and (**c**) corresponding specific capacitance as a function of scan rate. (**d**) Nyquist plots of Nb_4_C_3_T*_x_* MXene films, inset shows the high-frequency region of the spectra. (**e**) Linear fit showing the relationship between Real (Z) and ω^−1/2^ in the low-frequency region. (**f**) Schematic illustrating the transport of electrolyte ions through Nb_4_C_3_T*_x_* layers and ion diffusion pathways between MXene sheets and across an Nb_4_C_3_T*_x_* flake with a pinhole. Reproduced with permission from [63]. Copyright 2022 Elsevier.

Nasrin et al. [61] developed a new type of supercapacitor using Nb_2_C/Ti_3_C_2_ nanostructured 2D/2D MXenes that were interconnected and grown simultaneously. The device was obtained using a one-step chemical etching process that exposed and retained the active surface of the MXenes. This resulted in improved ion diffusion paths and charge storage kinetics, a remarkable potential window, as well as microstructural stability. The new supercapacitor showed the highest specific capacitance of 584 F g^−1^ at 2 A g^−1^ and an ED of 38.5 Wh kg^−1^ at a PD of 3840 W kg^−1^, with a remarkable cycling stability of 98% retention after 50,000 cycles. The enhanced performance was attributed to the undisentangled surface-active sites of the nanostructured interfacial interactions, which promotes a large increase in the pseudocapacitance of the two MXenes with broader operating voltages. Patra et al. [62] reported on the use of TiS_3_ nanosheets as a positive electrode in an asymmetric supercapacitor device, combined with Ti_3_C_2_T*_x_* as the negative electrode. The highest capacitance of TiS_3_ was 235 F g^−1^ at 5 mV s^−1^, with a battery-type charge storage mechanism, and the device demonstrated a cycle stability of 91%. Theoretical predictions and simulations showed that TiS_3_ materials have high-efficiency electrochemical storage capacity due to their high electrical conductivity, abundant electrochemically active sites, and fast faradaic redox kinetics. The charge storage activity can be tuned using various techniques such as phase engineering, defects, doping, and forming heterostructures or composites. Zhao et al. [63] developed a method to introduce nanopores into Nb_4_C_3_T*_x_* MXene sheets by adjusting the etching time. This method improved the ion diffusion paths, which were previously hindered by the restacking problem of 2D MXenes. The introduction of nanopores resulted in a 50% increase in rate capability within a charge/discharge time range of 1–2 s in 1 mol L^−1^ Li_2_SO_4_, Na_2_SO_4_, and (NH_4_)SO_4_ electrolytes. This method of introducing nanopores is cost-effective and minimizes the oxidation of the MXene, resulting in a high yield (Figure 3).

### 3.2. Ni-Co-Sulfides and MXenes

**Figure 4:**
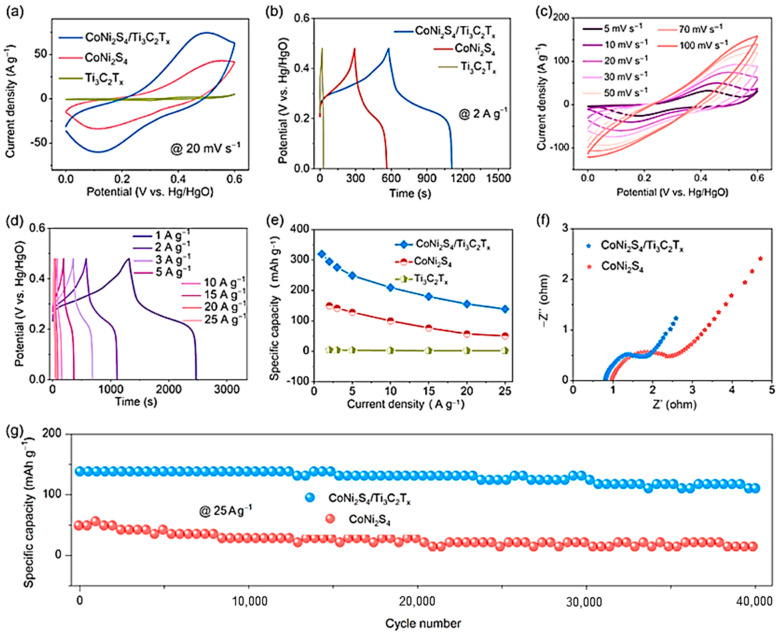
Electrochemical performance of the Ti_3_C_2_T*_x_*, CoNi_2_S_4_, and CoNi_2_S_4_/Ti_3_C_2_T*_x_* heteronanostructure. (**a**) CV curves at a scan rate of 20 mV s^−1^; (**b**) GCD curves at the current density of 2 A g^−1^; (**c**) CV curves at various scan rates; and (**d**) GCD curves at various current densities of the CoNi_2_S_4_/Ti_3_C_2_T*_x_* heteronanostructure; (**e**) specific capacity of different electrodes at various current densities; (**f**) Nyquist plots of CoNi_2_S_4_ and CoNi_2_S_4_/Ti_3_C_2_T*_x_*; (**g**) cycling stability of CoNi_2_S_4_ and CoNi_2_S_4_/Ti_3_C_2_T*_x_* at a current density of 25 A g^−1^ for 40,000 cycles. Reproduced with permission from [65]. Copyright 2023 Elsevier.

Ni-Co sulfide hybrid materials have been studied by He et al. [64]. They deposited Ni_1.5_Co_1.5_S_4_ nanoparticles on Ti_3_C_2_ nanosheets using a single-step hydrothermal method, which showed a high specific capacitance of 166.7 mAh g^−1^ at 1 A g^−1^ with a retention rate of 73.9% at 20 A g^−1^. The Ni_1.5_Co_1.5_S_4_/Ti_3_C_2_//activated carbon (AC) asymmetric device demonstrated an ED of 49.8 Wh kg^−1^ at a PD of 800 W kg^−1^ with 90% capacitance retention after 8000 cycles at 10 A g^−1^. This study suggests that multiscale tuning of atoms to components in hybrid systems can offer a feasible route for fabricating high-performance energy storage materials. Chen et al. [65] reported the formation of a sandwich-like nanostructure composed of CoNi_2_S_4_ and Ti_3_C_2_T*_x_* through a hydrothermal reaction. The CoNi_2_S_4_ nanosheets were uniformly distributed in the interlayer and on the surface of the Ti_3_C_2_T*_x_* MXene. This led to an increase in the interlayer distance of the host MXene, enabling fast ion movement and effectively accommodating the volume expansion of CoNi_2_S_4_. The electronic coupling between metals in CoNi_2_S_4_ and Ti_3_C_2_T*_x_* improved the electrical conductivity and optimized OH- uptake on the nanostructures. As a result, the CoNi_2_S_4_/Ti_3_C_2_T*_x_* nanostructured electrode showed a high specific capacitance of 320 mAh g^−1^ at 1 A g^−1^, maintaining 80% of its capacity even after 40,000 cycles at 25 A g^−1^ (see Figure 4). Good electronic coupling at the interface improves electrical conductivity by promoting stability and durability. It also enhances reactivity by increasing the absorption capacity for hydroxide ions.

Luo et al. [66] presented a simple method to prepare hierarchical transition metal sulfide-based electrodes for energy storage applications. A 2D hierarchical nanostructure of nickel cobalt sulfides (NiCoS) and ultrathin titanium carbide (Ti_3_C_2_) was prepared using co-precipitation and in situ sulfidation reactions. The interconnected porous network of NiCoS nanosheets on 2D Ti_3_C_2_ nanosheets led to a high surface area and active edge sites, improving the redox reaction kinetics. The combination of highly conductive and fast charge transfer Ti_3_C_2_ nanosheets and NiCoS resulted in the highest specific capacity of 759 C g^−1^ at 1 A g^−1^ with good rate capability. The assembled NiCoS/Ti_3_C_2_//activated carbon device delivered an ED of 22.6 Wh kg^−1^ at a PD of 400 W kg^−1^, with a good cycle performance of 91.2% after 10,000 cycles. In a second study, these researchers reported the preparation of a nickel sulfide/layered Ti_3_C_2_ (Ni-S/d-Ti_3_C_2_) nanostructured electrode using the solvothermal method [67]. The optimized Ni-S/d-Ti_3_C_2_ nanostructure showed the highest capacity of 840 C g^−1^ at 1 A g^−1^ and retained 64.3% at 30 A g^−1^. This result was attributed to the integration of d-Ti_3_C_2_ nanosheets, which can act as an electrical channel to accelerate electron transport at nanostructure interfaces during electrochemical reactions. The Ni-S/d-Ti_3_C_2_ nanostructure also showed good cycling stability when used as a cathode in an asymmetric device with a d-Ti_3_C_2_ thin film as the anode. Liu et al. [68] reported the preparation of a NiCo_2_Se_4_/Ti_3_C_2_T*_x_* nanostructure using a hydrothermal method. The conductive Ti_3_C_2_T*_x_* nanosheets were found to enhance the electrochemical performance of NiCo_2_Se_4_ and increase capacitance and charge storage through a synergistic effect. The NiCo_2_Se_4_/Ti_3_C_2_T*_x_* nanostructure showed the highest capacitance of 954 F g^−1^ at 1 A g^−1^, against 373.5 F g^−1^ for the NiCo_2_Se_4_ electrode alone. At this current density, the capacity retention was 93.9% after 3000 cycles. The assembled NiCo_2_Se_4_/Ti_3_C_2_T*_x_*//AC asymmetric device delivered a high ED of 22.4 Wh kg^−1^ at a PD of 800 W kg^−1^, with a 60.8% capacitance retention over 10,000 cycles at 5 A g^−1^, attributed to the fast ion/electrode transport facilitated by the conductive Ti_3_C_2_T*_x_*.

Zhang et al. [69] reported the preparation of Co_3_O_4_ nanoparticles immobilized on Ti_3_C_2_T*_x_* (Co-Ti_3_C_2_T*_x_*) nanostructures through a self-assembly process. They found that the Co-Ti_3_C_2_T*_x_* nanostructures showed the highest capacitance of 1081 F g^−1^ at 0.5 A g^−1^, while bulk Ti_3_C_2_T*_x_* electrodes had a capacitance of 89 F g^−1^. The assembled Co-Ti_3_C_2_T*_x_*//polyaniline-derived carbon on carbon fiber paper (PANI-C@CFP) device delivered an ED of 26.06 Wh kg^−1^ at a PD of 700 W kg^−1^ with 83% retention after 8000 cycles at 2 A g^−1^, due to the synergistic effect between Ti_3_C_2_T*_x_* and Co_3_O_4_ nanoparticles that improved both electrochemical activity and conductivity. The study suggests that coupling metal oxides to MXenes can enhance storage capacity due to their structural features with exposed active sites. Zhao et al. [70] reported the covalent functionalization of Ti_3_C_2_Cl_2_ nanodots dispersed on NiAl layered double hydroxides (LDH), resulting in an electrode with a capacitance of 2010 F g^−1^ at 1 A g^−1^, an ED of 100.5 Wh kg^−1^ at a PD of 300 W kg^−1^, and maintained 94.1%. The improved performance was attributed to the increased number of active sites and enhanced electrical conductivity of the Ti_3_C_2_Cl_2_ nanodots. The study demonstrates the potential of interlayer assembly of 2D layered materials with NiAl-LDH.

### 3.3. Vanadium Sulfide MXenes

Sharma et al. [71] prepared Ti_3_C_2_T*_x_* and 1T-VS_2_ nanosheet hybrids for supercapacitor applications through the hydrothermal method and studied the electrochemical activity of Ti_3_C_2_T*_x_*/1T-VS_2_ nanostructures with different ratios of Ti_3_C_2_T*_x_*. The Ti_3_C_2_T*_x_*/1T-VS_2_ hybrid device showed a high capacitance of 116 F g^−1^ at 0.8 A g^−1^, an operating voltage of 1.6 V, an ED of 41.13 Wh kg^−1^ at a PD of 793.5 W kg^−1^ with 85% capacitance retention and 100% Coulombic efficiency after 5000 cycles. This performance was attributed to the synergistic effect and charge storage kinetics of the Ti_3_C_2_T*_x_*/1T-VS_2_ nanostructure. The lower diffusion energy barrier of electrolytic ions in the Ti_3_C_2_T*_x_*/1T-VS_2_ hybrid allowed higher charge storage and enhanced the capacitance. The results were supported in DFT studies, which predicted lower diffusion energy barriers and higher capacitance due to the synergistic effect between VS_2_ and Ti_3_C_2_T*_x_*. Chen et al. [72] reported on a study of an asymmetric supercapacitor composed of a cathode made of hydrothermally synthesized Ti_3_C_2_T*_x_*/VS_2_ nanostructure and an anode made of Fe_3_O_4_@rGO hydrogel. The cathode showed a high specific capacity of 896 C g^−1^ (228.4 F g^−1^) at 1 A g^−1^, with a retention of 90.6% after 10,000 cycles at 20 A g^−1^. This was attributed to the microstructure with connected nanosheets and the synergistic effect of VS_2_ and conductive Ti_3_C_2_T*_x_* enhancing electrochemical conductivity. The asymmetric supercapacitor had a specific capacitance of 365.4 C g^−1^ at 1 A g^−1^, an ED of 73.9 Wh kg^−1^, and a PD of 728.2 W kg^−1^. The device maintained 90.7% capacitance after 10,000 cycles, indicating its potential for energy storage applications.

MXenes are prone to agglomeration or stacking with oxidation-labile surfaces and layered structures, which hinder their practical application prospects in energy utilization and storage devices. To address this issue, Li et al. [73] presented a strategy for transforming layered TiVCT*_x_* nanosheets into 3D stable tremella-like structured TiVCT*_x_*/poly-o-phenylenediamine (N-TiVCT*_x_*) nanostructures, using o-phenylenediamine (oPD) as a building block. The N-TiVCT*_x_* nanostructures showed improved stability and electrochemical behavior compared to TiVCT*_x_*, with a capacitance of 282 F g^−1^ at 10 mV s^−1^, a 50% increase compared to TiVCT*_x_*. The authors attribute the improved performance of N-TiVCT*_x_* to its tremella-like structure, which provides more efficient ion transport channels and large electrochemical interfaces, thereby maximizing the advantages of electrode capacity in energy storage devices.

### 3.4. Mo_2_Ti_2_C_3_ MXene

A supercapacitor using Mo_2_Ti_2_C_3_ MXene as the free-standing film electrode was reported by Gandla and co-workers [74]. The supercapacitor used 1mol L^−1^ 1-ethyl-3-methylimidazolium bis-(trifluoromethylsulfonyl)-imide (EMIMTFSI) in an acetonitrile electrolyte. Using etching and vacuum-assisted filtration techniques, the researchers achieved a layer spacing of 2.4 nm in the Mo_2_Ti_2_C_3_ MXene electrode without using any pre-intercalator. The symmetric Mo_2_Ti_2_C_3_ device delivered an ED of 188 Wh kg^−1^ at a PD of 22 kW kg^−1^ and a highest capacitance of 152 F g^−1^. These results were considered remarkable compared to other MXene-based electrodes.

### 3.5. Mo_2_CT_x_ MXenes

Mo_2_CT*_x_* is a two-dimensional material (MXene) made from Mo_2_Ga_2_C either by etching Ga in HF or using the polymer intercalation method [75,76]. Mo_2_CT*_x_* exhibits efficient electrocatalytic properties for hydrogen evolution and is used in the development of energy storage devices. The exact etching mechanism of Mo_2_CT*_x_* from Mo_2_Ga_2_C is not known and requires further research. Halim et al. [77] prepared Mo_2_CT*_x_* from Mo_2_Ga_2_C bulk using the polymer intercalation method. However, the etching mechanism of Mo_2_CT*_x_* from bulk Mo_2_Ga_2_C has not been determined, and further research on this topic is needed. Mo_2_CT*_x_*, a two-dimensional molybdenum carbide, has shown potential as an electrode for electrochemical energy storage due to the multiple oxidation states of Mo and its intrinsic properties [78]. However, more research is needed to understand the mechanisms and effects of different electrolytes on the electrochemical properties of Mo_2_CT*_x_*.

A recent study by He et al. [79] focused on the effect of different electrolytes (1 mol L^−1^ KOH, MgSO_4_, and H_2_SO_4_) on the supercapacitor performance of Mo_2_CT*_x_* MXene. The Mo_2_CT*_x_* was prepared through hydrothermal etching, and the capacitance of the electrodes was measured at a current density of 0.3 A g^−1^. The results showed that H_2_SO_4_ is the most suitable electrolyte for the MXene-based electrodes as it exhibited the highest capacitance of 79.14 F g^−1^, corresponding to a volumetric capacitance of 390.7 F cm^−2^. The retention value of 98% after 5000 cycles further emphasizes the suitability of H_2_SO_4_ for Mo_2_CT*_x_*-based supercapacitor applications. The study highlights the importance of selecting the right electrolyte for enhancing the performance of MXene-based supercapacitors.

### 3.6. Mo_1.33_CT_z_ MXenes

El-Ghazaly et al. [80] reported that the electrochemical behavior of Mo_1.33_CT_z_ MXene in sulfate-based aqueous electrolytes with univalent (Li^+^, Na^+^, and K^+^) or divalent (Mg^2+^, Mn^2+^, and Zn^2+^) cations was explored. The results showed that the Mo_1.33_CT_z_ electrodes could operate in a potential window above 1.0 V without degradation in these electrolytes. The Mo_1.33_CT_z_ electrodes had the highest volumetric capacitance of 677 F cm^−3^ in 1 mol L^−1^ MnSO_4_ solution. Asymmetric devices using Mo_1.33_CT_z_ and N-doped activated carbon in 0.5 mol L^−1^ K_2_SO_4_ solution can operate with a cell potential of 1.8 V and retain 97% of their initial capacitance after 5000 cycles. The study suggested that the choice of intercalating cations is a viable strategy to enhance the electrochemical performance of Mo_1.33_CT_z_-based electrodes for energy storage applications.

## 4. Functionalization of MXene for Supercapacitor

### 4.1. Approach to Functionalize MXene

The unmodified MXene material exhibits poor performance due to its poor mechanical stability and low capacitance. To address this challenge, the functionalization of MXene to improve its performance in supercapacitors has been extensively studied in recent years. Functionalization of MXene refers to the modification of the surface or interface of the MXene material with various chemical groups or nanomaterials. This functionalization can enhance the mechanical stability, capacitance, and cycling stability of the MXene material. Several methods have been used, including chemical modification, electrochemical modification, and the incorporation of nanomaterials. Chemical modification is a simple and effective approach to functionalizing MXene. This approach involves the reaction of the MXene material with various chemical species, such as acids, bases, or organic molecules, to introduce new functional groups onto the surface of the MXene. For example, the introduction of oxygen-containing functional groups such as carboxylic acids or hydroxyl groups can improve the mechanical stability of the MXene material and increase the number of active sites for ion adsorption. Additionally, the introduction of nitrogen-containing functional groups such as amines or nitriles can improve the capacitance and stability of the MXene material in aqueous environments.

Electrochemical modification is another approach to functionalizing MXene. This approach involves the use of an electrochemical process to modify the surface of the MXene material with various chemical species. The electrochemical process can either introduce new functional groups onto the surface of the MXene or modify existing functional groups to improve the performance of the MXene material in supercapacitors. For example, the electrochemical modification of MXene with graphene oxide improves the mechanical stability and capacitance of the MXene material.

Finally, the incorporation of nanomaterials is another approach to functionalizing MXene. This approach involves the integration of nanomaterials such as graphene or carbon nanotubes into the MXene material to improve its performance in supercapacitors. The nanomaterials can improve the mechanical stability and electron transport efficiency of the MXene material, leading to higher capacitance and stability. Additionally, the integration of nanomaterials can also enhance the capacitance of the MXene material by increasing the number of active sites for ion adsorption. In conclusion, the functionalization of MXene is a crucial step in the development of high-performance supercapacitors based on MXene materials. The various functionalization methods discussed above, including chemical modification, electrochemical modification, and the incorporation of nanomaterials, have shown promising results in improving the mechanical stability, capacitance, and cycling stability of the MXene material. Further research in this area is needed to fully understand the underlying mechanisms of functionalization and to develop new functionalization methods that can further improve the performance of MXene-based supercapacitors. Deep eutectic solvents (DESs) are a new type of solvent that has recently gained attention for their potential use in the etching of Ti_3_C_2_T*_x_*. DESs are composed of a mixture of two or more components that form a low melting point and thermodynamically stable compound. DESs are known for their chemical stability, inherent safety, excellent compatibility, and low cost, making them attractive alternatives to traditional toxic, volatile, and flammable solvents. In the etching of Ti_3_C_2_T*_x_*, the type of solvent used can have a crucial effect on the type and amount of surface termination. DESs have been shown to be effective in etching Ti_3_C_2_T*_x_* and producing high-quality MXene materials. Particularly, DESs can control the surface termination and reduce the quantity of residual contaminants on the surface of the MXene, leading to improved performance and stability. One example of a DES that has been used in the etching of Ti_3_C_2_T*_x_* is choline chloride/ethylene glycol. This DES has been shown to effectively etch Ti_3_C_2_T*_x_*, producing high-quality MXene materials with improved surface termination and reduced residual contaminants. Additionally, this DES has been found to be safe, compatible, and low-cost, making it an attractive alternative to traditional solvents. In conclusion, the use of DESs in the etching of Ti_3_C_2_T*_x_* has emerged as a promising approach to producing high-quality MXene materials. The chemical stability, inherent safety, excellent compatibility, and low cost of DESs make them attractive alternatives to traditional solvents. Further research is needed to fully understand the impact of DESs on the etching of Ti_3_C_2_T*_x_* and to develop new DESs that can further improve the performance and stability of MXene materials. For example, Gong et al. [81] reported a water-free etching method using deep eutectic solvents, resulting in functionalized Ti_3_C_2_T*_x_* with abundant –O end groups and low oxidation degree, leading to excellent cycle stability. Kim et al. also showed that Ti_3_C_2_T*_x_*, functionalized with deep eutectic solvents, has good capacitive properties [82].

Yun et al. [83] reported a simple method to improve the stability of delaminated Ti_3_C_2_T*_x_* by passivating its vulnerable edges with heterocyclic aromatic amines. The use of pyrrole functionalization was found to provide anti-oxidation in aqueous electrolytes at room temperature and under high temperature and oxidizing conditions. The pyrrole-functionalized Ti_3_C_2_T*_x_* electrode showed a significant improvement in specific capacitance compared to a pyridine-functionalized electrode, with a value of 253.6 F g^−1^ compared to 178 F g^−1^. This improvement is attributed to the strong chemical interaction between pyrrole and Ti_3_C_2_T*_x_* and the intercalation effect that it creates. Li et al. [84] synthesized a 3D metal/Ti_3_C_2_ derivative nanostructure through a simple alkalization and metal ion pre-intercalation process. This process effectively prevented the restacking of Ti_3_C_2_ nanosheets and allowed the use of the Zn/Ti_3_C_2_ nanostructures as anodes in zinc-ion capacitors. The resulting Zn-ion capacitor showed a high capacity of 75.2 mAh g^−1^ and an ED of 60.2 Wh kg^−1^, maintaining 92.5% of its capacity after 10,000 cycles at 3.3 A g^−1^. The study provides an effective strategy for the development of next-generation high-efficiency energy storage systems.

A femtosecond laser ablation method was used to fabricate flexible Ti_3_C_2_T*_x_* ribbon-based electrodes for supercapacitors [85]. These ribbons had a high surface area and porous edges with exposed continuous layered channels, which improved ion accessibility and storage. The resulting Ti_3_C_2_T*_x_* ribbons showed the highest capacitance of 1308.3 mF cm^−3^ at a scan rate of 2 mV s^−1^ with a good rate capability of 95% and a Coulombic efficiency of 92% over 30,000 cycles. The capacitance retention was 81.8% when the scan rate was increased to 200 mV s^−1^. This was attributed to the high surface area increasing the ion-accessible solvated H^+^, the exposed ion transmission channels, the presence of mesopores, and the continuous layered channels promoting redox reactions. This design is considered significant for the development of next-generation flexible supercapacitor devices. Vaghasiya et al. [86] demonstrated a flexible supercapacitor made of fluorinated Ti_3_C_2_T*_x_* using a fluorination strategy. Fluorine was inserted as a heteroatom into the Ti_3_C_2_T*_x_* structure, which improved its structure, wettability, and electrochemical performance. The study also explored the effect of different metal cations such as Ti, Ta, V, Cr, and Mo on the fluorinated MAX phase electrodes and found that fluorinated materials improved the capacitance and PD of the electrodes. The symmetric flexible devices of F-Ti_3_AlC_2_ and F-Mo_2_TiAlC_2_ showed remarkable electrochemical activity. The results indicate that heteroatom doping has a significant impact on the morphology and electrochemical activity of MAX materials, providing a new approach for developing high-performance MAX electrodes for memory devices.

Prabhakar et al. [87] described the optimization of Ti_3_C_2_T*_x_*-based electrodes for supercapacitors by sonochemically anchoring SnO_2_ nanoparticles in a KOH electrolyte. By layering the Ti_3_C_2_T*_x_* with tetramethylammonium hydroxide and introducing SnO_2_ nanoparticles, they were able to achieve the highest capacitance of 669 F g^−1^, with a retention of 90% over 6000 cycles. The layering process was found to be crucial in controlling the phase transition and morphology of the Ti_3_C_2_T*_x_*, leading to enhanced ion migration and electron transport in the storage device. Guan et al. [88] reported the fabrication of porous Ti_3_C_2_T*_x_* nanosheets as electrodes for high-performance supercapacitors. The porous sheets were obtained through partial oxidation and etching with H_2_O_2_ and HCl solutions. The resulting electrodes had a high capacitance of 385 F g^−1^ at 1 A g^−1^, with good retention of 92% over 10,000 cycles at 100 mV s^−1^, demonstrating the potential of porous Ti_3_C_2_T*_x_* nanosheets as promising electrodes for supercapacitor applications. Liu et al. [89] developed an electrode for supercapacitor applications made of Ti_3_C_2_T*_x_*@PANI-coated activated carbon cloth (Ti_3_C_2_T*_x_*@PANI-ACC), which has a 2D/0D/1D hierarchical nanostructure. The 1D carbon fibers were coated with Ti_3_C_2_T*_x_*@PANI nanosheets, solving the restacking problem of MXene leading to good conductivity for fast electron transfer. This nanostructured electrode has a high capacitance of 1347 mF cm^−1^ at 1 mA cm^−2^ and retained 81% of the capacitance after more than 5000 cycles at 20 mA cm^−2^.

**Figure 5:**
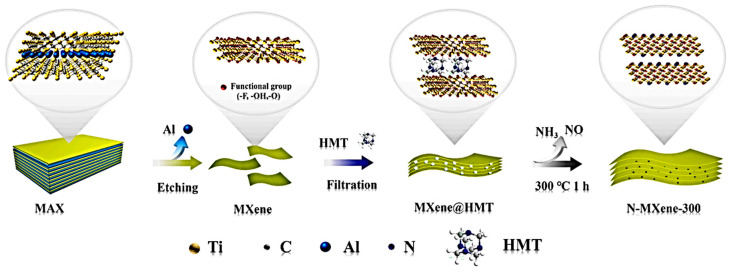
Schematic illustration of the synthesis process of N-MXene films. Reproduced with permission from [92]. Copyright 2022 Elsevier.

The modification of the surface functional groups on the Ti_3_C_2_T*_x_* lattice has been shown to enhance the electrochemical activity of Ti_3_C_2_T*_x_* [90]. Heteroatom doping of the surface functional groups can generate proper surface electron density and create chemical reaction sites. Replacing low electronegativity functional groups with N-functional groups has been shown to increase the electron density of Ti_3_C_2_T*_x_*. Surface replacement also avoids the decrease in conductivity of Ti_3_C_2_T*_x_*, as seen in nitrogen-doped Ti_3_C_2_T*_x_*, where both lattice substitution of carbon atoms and surface substitution of terminal functional groups occur [91]. The substitution of C atoms with N elements in Ti_3_C_2_T*_x_* can increase its capacitance, but it also weakens the electrical conductivity of the Ti_3_C_2_T*_x_* due to the destruction of the Ti-C bonds in the lattice. This is because the substitution process leads to changes in the material’s electronic properties, which can negatively impact its conductivity. Therefore, lattice substitution has a limited impact on the electrical conductivity of Ti_3_C_2_T*_x_*. For example, Shi et al. [92] reported the development of flexible N-doped Ti_3_C_2_T*_x_* films using carbohexamethylenetetramine (HMT) molecules (Figure 5).

**Figure 6:**
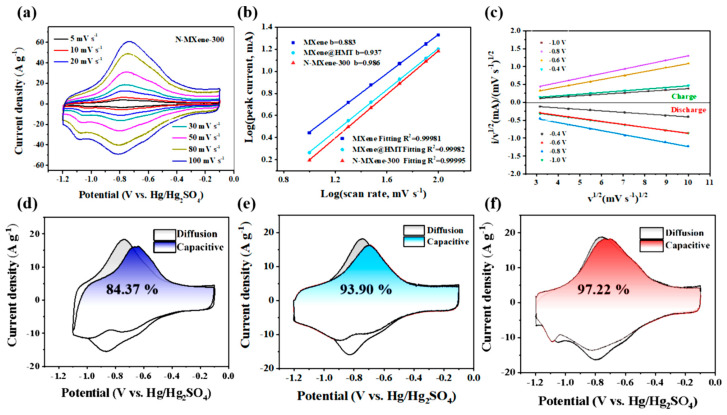
(**a**) CV curves at different scan rates of N-MXene-300, (**b**) the plots log i vs. log ν, (**c**) the curves of i/v^1/2^ vs. ν^1/2^, (**d**–**f**) the capacitive contribution at 30 mV s^−1^ of MXene, MXene@HMT and N-MXene-300. Reproduced with permission from [92]. Copyright 2022 Elsevier.

The HMT molecules were coupled with Ti_3_C_2_T*_x_* through hydrogen and coordination bonds, which expanded the interlayer spacing and ensured high electrical conductivity by preserving the Ti-C bond of Ti_3_C_2_T*_x_*. The N content in HMT was used to replace the surface functional groups on the Ti_3_C_2_T*_x_* surface with N-functional groups during the carbonization process. The N-doped Ti_3_C_2_T*_x_* calcined at 300 °C showed the highest capacitance of 193 F g^−1^ at 2 mV s^−1^ with the help of HMT, and an asymmetric device assembled using N-doped graphene aerogel provided a high ED of 26.22 Wh kg^−1^ with an efficiency of 92.1% over 25,000 cycles, suggesting potential electrochemical applications (Figure 6).

The etching process of MXene can result in abundant surface terminations that not only improve hydrophilicity but also create active sites for surface redox reactions, thus enhancing the electrochemical behavior and pseudocapacitive behavior of MXene-based electrodes. However, some terminations, such as –F, can reduce the electrochemical activity. Regulating the surface termination type is, therefore, crucial for optimizing the electrochemical activity of MXene-based electrodes [93]. For instance, the functionalization of MXenes with iodine terminations (I-Ti_3_C_2_) through a facile Lewis acid melt etching method was reported by Gong et al. [94]. I-Ti_3_C_2_ showed better capacitive performance compared to the hydrofluoric acid etched MXene (HF-Ti_3_C_2_) and showed excellent cycle life with a capacitance loss of only 0.09% per cycle after 100,000 cycles. Wei et al. [95] reported a P-doping of Ti_3_C_2_T*_x_* achieved using sodium hypophosphate, which increased the interlayer spacing of Ti_3_C_2_T*_x_* and formed P-O and P-C bonds in the material. This led to faster pathways for electrolyte ion migration and better pseudocapacitance, resulting in higher capacitance compared to bare Ti_3_C_2_T*_x_*. The flexible electrode with P-doped Ti_3_C_2_T*_x_* showed a capacitance of 476.9 F g^−1^, while a flexible quasi-solid device assembled from P-doped Ti_3_C_2_T*_x_* thin film provided a capacitance of 103 F g^−1^ at 5 mV s^−1^. The improved structure, composition, and electrochemical performance of Ti_3_C_2_T*_x_* by P-atom doping, surface modification, and functionalization of MXene materials contribute to the high ED of 15.8 Wh kg^−1^ and 6.1 Wh kg^−1^ at 250 W kg^−1^ and 10 kW kg^−1^, respectively. Khan et al. [96] reported a method for etching MAX phases (fluorine-free Ti_3_C_2_ with -Cl, -I, and -Br halogen terminations) using a molten salt synthesis strategy and direct redox coupling. The resulting materials (Ti_3_C_2_Cl_2_, Ti_3_C_2_I_2_, and Ti_3_C_2_Br_2_) were used as electrode materials for supercapacitors and showed capacities of 92, 63, and 29 C g^−1^, respectively, in 3 mol L^−1^ H_2_SO_4_, with retentions of 32%, 49.1%, and 85.22% after 10,000 cycles, respectively. The etched powder, MS-Ti_3_C_2_, was prepared by immersing Ti_3_AlC_2_ in a molten salt of CuCl_2_, CuI_2_, or CuBr_2_, followed by wet chemical etching. Single-surface functional groups were obtained as a result of these reactions. Wang et al. [97] developed a free-standing and flexible solid-state supercapacitor using MXene/graphdiyne nanotube composite films (graphdiyne is a new two-dimensional carbon allotrope). The composite films enhanced the ion flux fraction, creating a 3D transport highway for interlayer ions. The result was a high capacitance of 337.4 F g^−1^, with a rate capability of 73% at 100 mV s^−1^ and a capacitance retention of 88.2% after 10,000 cycles. The assembled thin-film asymmetric device showed a high capacitance of 65.3 F g^−1^ at a PD of 750 W kg^−1^ and an ED of 19.7 Wh kg^−1^.

Prenger et al. [98] used metal cations (Na^+^, K^+^, and Mg^2+^) pre-intercalated multi-layer Ti_3_C_2_T*_x_* acting as electrodes for aqueous supercapacitors. The study showed that K-Ti_3_C_2_T*_x_* had the highest capacitance of 300 F g^−1^ and an excellent areal capacitance of 5.7 F cm^−2^, which was 10 times higher than the layered MXene and exceeded the 4 F cm^−2^ of microengineered Ti_3_C_2_T*_x_* electrodes. The variation in Ti oxidation states indicated that the charge storage in the Ti_3_C_2_T*_x_* pre-intercalated with K^+^ or Na^+^ is larger than with Mg^2+^. By using wet spinning of sheared Ti_3_C_2_T*_x_* sediments, He et al. [99] obtained tightly packed Ti_3_C_2_T*_x_* nanosheets, forming ultradense fibers with high electrochemical performance. The fibers had a density of 5.4 g cm^−3^, high conductivity, and a capacitance of 1661 F cm^−3^ in 1 mol L^−1^ H_2_SO_4_ electrolyte. The volume capacitance was 875 F cm^−3^ in a semi-solid electrolyte, and the capacity retention rate after 500 cycles was 93%. The fiber-based device showed an ED of 105.7 mWh cm^−3^ at a PD of 500 mW cm^−3^. Tian et al. [100] showed that oxygen doping of Ti_3_C_2_T*_x_* MXene nanosheets can be achieved through an in situ process where oxygen atoms replace some of the carbon atoms in the Ti octahedra, which enhanced the interlayer space, interfacial charge transport, and electronic conductivity. The oxygen-doped Ti_3_C_2_T*_x_* thin-film electrodes showed a higher capacitance (360 C g^−1^) compared to the bare Ti_3_C_2_T*_x_* (216.8 C g^−1^) due to higher Ti metal active centers and higher adsorption energy, while the quantum capacitance was improved by oxygen heteroatoms. The study provides a general strategy for the oxygen doping of Ti_3_C_2_T*_x_* and suggests that this process is easier than complicated post-doping methods. Yildirim et al. [101] demonstrated the effect of confinement in acidic nanotemplates on the polymerization of pyrrole (PPy) and titanium carbide (Ti_3_C_2_T*_x_*), leading to improved electrical conductivity and electrochemical behavior with good cycling stability. The mechanism of oxidant-free pyrrole polymerization between the Ti_3_C_2_ surface and Ti_3_C_2_T*_x_* interlayer was studied using first-principles calculations. The polymerization was found to be initiated by hydrogen bonding between the pyrrole monomer and the surface oxygen, with the proton transferred from the surface hydroxyl to the β-carbon of pyrrole, increasing its reactivity and initiating polymerization. The efficiency of the reaction was found to be controlled by the density of surface hydroxyl groups, which act as proton sources and control the interlayer distance. Hao et al. [102] developed a strategy to enhance the resistance to oxidation and the structural stability of Ti_3_C_2_T*_x_* MXene films for high-performance flexible supercapacitors by functionalizing the Ti_3_C_2_T*_x_* with tannic acid bridging agents containing O-containing ligands. The resulting bridged Ti_3_C_2_T*_x_* films showed improved interlayer interaction, resistance to oxidation and swelling, improved toughness (7-fold improvement compared to bare Ti_3_C_2_T*_x_*), stable electrical conductivity, good flexibility, and electrochemical stability over 10,000 cycles. The charge transfer from the Ti_3_C_2_T*_x_* to O-rich molecules enhanced the interfacial electronic structure, increasing the work function of the bare MXene and improving resistance to electron loss and oxidation.

Liu et al. [103] have presented a method for constructing Ti_3_C_2_T*_x_*-based electrodes that have improved electrical performance. The method involves a modification of the surface termination and the creation of an extended interlayer spacing using 3D nanostructures. This results in a negative electrode that has an excellent capacitance of 652.3 F g^−1^, which is three times higher than that of pristine Ti_3_C_2_T*_x_*, and a capacitance retention of 81% after 10,000 cycles at 50 A g^−1^. The assembled symmetric supercapacitor showed an ED of 20.3 Wh kg^−1^ at a PD of 500 W kg^−1^. The Ti_3_C_2_T*_x_*/PANI electrode was also developed as a free-standing thin film, showing a bulk capacitance of 2368 F cm^−3^, making it a better anode than many other MXene-based anodes. Lee et al. [104] reported the creation of a multiscale porous Ti_3_C_2_T*_x_* material through a process of partial oxidation with hydrogen peroxide and flocculation in acidic media. The resulting material had a 5-times higher specific surface area compared to pristine Ti_3_C_2_T*_x_* and showed excellent capacitance (307 F g^−1^ at 20 mV s^−1^ and 225 F g^−1^ at 100 mV s^−1^ in 1 mol L^−1^ H_2_SO_4_) and good capacitance retention (87.6% after 6000 cycles) due to the combination of mesopores that facilitate ion diffusion, and wrinkled macropores that reduce the restacking problem of MXene.

Yang et al. [105] studied the effect of various solvents on the functionalization of Ti_3_C_2_ and developed N-doped Ti_3_C_2_ thin films using a solvothermal method. The best result was obtained using the auxiliary solvent C_3_H_8_O, which increased the d-spacing of Ti_3_C_2_, resulting in the highest capacitance of 2846.5 F cm^−3^ after 10,000 cycles. The assembled symmetric device had the highest volumetric ED of 64 Wh L^−1^ at a PD of 118 W L^−1^ at 1 V s^−1^. Ghosh et al. [106] reported a new reaction condition for the preparation of Ti_3_C_2_T*_x_* for supercapacitor applications. They used NaBF_4_ in aqueous HCl as a reaction condition, which is a cost-effective method for synthesizing Ti_3_C_2_T*_x_*. The optimum reaction temperature was found to be 130 °C. The electrode obtained from this method showed a capacitance of 262 F g^−1^ in 1 mol L^−1^ H_2_SO_4_, and the assembled asymmetric device had a capacitance of 60 F g^−1^ and an ED of 10.8 Wh kg^−1^ at a PD of 408 W kg^−1^. Fan et al. [107] described a novel approach to enhance the performance of Ti_3_C_2_ composite films as supercapacitors by functionalizing them with a combination of polypyrrole (PPy) and ionic liquid (IL) double-spacer microemulsion particles. The combination of PPy and IL-based microemulsion particles increased the capacitance and energy density of the functionalized Ti_3_C_2_ nanostructure and showed excellent capacitance (51.85 F g^−1^ at 20 mV s^−1^) with an ED of 31.2 Wh kg^−1^ and a Coulombic efficiency of 91% after 2000 cycles. Sun et al. [108] proposed a method for fabricating solid microsupercapacitors using nitrogen and sulfur co-doped MXene (N,S-Ti_3_C_2_T*_x_*) ink without additives. The N,S-Ti_3_C_2_T*_x_* ink was designed for inkjet printing and showed high oxidation stability and electrochemical performance. The resulting N,S-Ti_3_C_2_T*_x_* material had a gravimetric capacitance of 266 F g^−1^ and a volumetric capacitance of 710 F cm^−3^. It also showed an ED of 8.9 mWh cm^−3^, a cycle stability of 94.6% at a PD of 411 mW cm^−1^, and excellent performance, customization, and connectivity. The N and S doping atoms were treated using N_2_ protection annealing and thiourea solvothermal treatment, which removed MXene surface defects and increased the number of active sites. The enhancement of redox reactivity, H^+^ adsorption, oxidation resistance, and reaction kinetics was attributed to the doping of N and S atoms in the microelectrodes. Hwang et al. [109] presented a hybrid asymmetrical supercapacitor device composed of a cathode material rGO decorated with phosphomolybdic acid (PMo_2_) polyoxometalate (POM) and anode material Ti_3_C_2_T*_x_*. The complementary voltage and redox activity of Ti_3_C_2_T*_x_* and rGO-POM, combined with the 2D characteristics of rGO, enhance the device’s electrochemical activity. The asymmetrical device showed an ED of 50.5 Wh kg^−1^ at a PD of 7 kW kg^−1^, a Coulombic efficiency of 87.12% after 10,000 cycles, and good energy and Coulombic efficiencies at all current densities, suggesting that the rGO-POM cathode can effectively enhance the electrochemical activity of the hybrid supercapacitor by coupling the proton electrolyte with the Ti_3_C_2_T*_x_* anode.

### 4.2. Vacancies/Defects of MXene-Based Supercapacitors

Vacancies and defects play a crucial role in the performance of MXene-based supercapacitors. The presence of vacancies and defects can affect the electrical conductivity, surface area, and ion transport properties of MXene materials, leading to changes in the capacitance and energy storage performance of supercapacitors. Vacancies in the MXene materials can increase electrical conductivity by creating charge carriers and enabling rapid ion transport. On the other hand, excessive vacancies can also lead to decreased capacitance and reduced energy storage performance by reducing the surface area and disrupting the stability of the material. Similarly, defects in the MXene materials can also affect the performance of supercapacitors. Defects can act as charge traps, reducing the electrical conductivity and leading to decreased capacitance and energy storage performance. Additionally, defects can also lead to structural instability and material degradation over time, further reducing the performance and lifespan of the supercapacitor. Therefore, controlling the amount and distribution of vacancies and defects in MXene materials is critical to optimizing the performance of MXene-based supercapacitors. Techniques such as post-synthesis treatment, surface modification, and material design can be used to control the amount and distribution of vacancies and defects, leading to the improved performance and stability of MXene-based supercapacitors. In conclusion, vacancies and defects play a significant role in the performance of MXene-based supercapacitors. A proper balance of vacancies and minimal defects is necessary for optimal performance, and efforts to control and optimize these properties are ongoing in the field. One example of how vacancies can improve the performance of MXene-based supercapacitors is the creation of a pseudocapacitance. Pseudocapacitance refers to the capacitance that arises from chemical reactions occurring at the surface of the material, in addition to the electrical double-layer capacitance that arises from the separation of charges at the surface. Vacancies in MXene materials can provide active sites for pseudocapacitance to occur, leading to improved energy storage performance.

Another example of how defects can affect the performance of MXene-based supercapacitors is their impact on the ion transport properties. Defects in MXene materials can act as ion traps, reducing the ion transport efficiency and leading to decreased capacitance and energy storage performance. Efforts to control the size and distribution of defects by surface modification and material design can improve the ion transport properties and overall performance of MXene-based supercapacitors. In terms of material design, the use of heterostructured MXene materials has been shown to improve the performance of MXene-based supercapacitors. They are composed of two or more different MXene species, with each species having unique electronic and ion transport properties. By combining these species into a heterostructured material, it is possible to create a material with improved electronic and ion transport properties, leading to the improved performance of MXene-based supercapacitors. In conclusion, the role of vacancies and defects in MXene-based supercapacitors is complex and multifaceted. Vacancies can improve the performance of supercapacitors through the creation of pseudocapacitance, while defects can decrease performance through reduced ion transport efficiency. Techniques such as post-synthesis treatment, surface modification, material design, and the use of heterostructured materials are ongoing efforts to control and optimize the properties of MXene-based supercapacitors.

Recent studies have shown that 2D nanosheets of the ordered quaternary material 211 MAX phase composed of (Mʹ_1.33_M″_0.66_)AlC exhibit higher electrochemical behavior when a small amount of the transition metal M” is etched away, leaving in-plane ordered double vacancies [110]. This material, called i-MXene, has a bulk capacitance of up to 1380 F cm^−3^ in H_2_SO_4_ electrolyte. The Mo_1.33_CT*_x_* electrodes rich in Mo vacancies show higher volumetric capacity and energy density in 1 mol L H_2_SO_4_ [111]. Etman et al. [112] demonstrated that the presence of vacancies in Mo_1.33_CT*_x_* MXene materials can improve the electrochemical behavior and performance of supercapacitors. For example, Etman et al. reported a bulk capacitance of 1380 F cm^−3^ in H_2_SO_4_ electrolyte for Mo_1.33_CT*_x_*/Ti_3_C_2_T*_x_* nanostructured films, while Zheng et al. [113] found that Mo_1.33_CT*_x_* electrodes rich in Mo vacancies exhibited higher volumetric capacity and energy density compared to electrodes without vacancies. The symmetric device of Mo_1.33_CT*_x_* with a high concentration of Mo vacancies delivered the highest ED of 25.4 mWh cm^−3^ at a PD of 152.4 mW cm^−3^, with a voltage retention of 65.4% over 10 h in 15 mol L^−1^ LiBr electrolyte. However, both the Mo_1.33_CT*_x_* and Mo_2_CT*_x_* electrodes were found to exhibit high self-discharge behavior, which needs to be further addressed for the improvement of the performance and stability of MXene-based supercapacitors. These findings highlight the importance of controlling and optimizing the properties of MXene materials, such as the concentration of vacancies. Ongoing research in this field will likely lead to new and improved energy storage technologies based on these materials. Liu et al. [114] developed a high-efficiency hybrid supercapacitor made of NiMnZn-LDH/Mo_2_CT*_x_* nanostructures. They used alkali-etched NiMnZn-LDH nanosheets and exfoliated Mo_2_CT*_x_* to produce an electrostatic assembly. The strong interaction of the two components modulates the surface electronic structure of LDH and increases the number of oxygen vacancies. The LDH/Mo_2_CT*_x_* nanostructure showed a capacity of 1577 C g^−1^ and good cycling stability. The asymmetric device of LDH/Mo_2_CT*_x_*//Fe_2_O_3_/CNTs electrodes had an ED of 92.6 Wh kg^−1^ at PD of 2695 W kg^−1^ and good retention. The Mo_2_CT*_x_*-based device has several advantages: (i) the exposure of the surface or edge sites of Mo_2_CT*_x_* is enhanced, which improves its electrochemical performance; (ii) tuning the valence states of Ni/Mn active atoms and generating abundant oxygen vacancies in LDH enhance its electrochemical properties; (iii) the strong coupling between LDH and Mo_2_CT*_x_* leads to an improved surface electronic structure and oxygen vacancy content of LDH, and enhances the performance of Mo_2_CT*_x_*-based energy storage devices. Overall, the results highlight the benefits of functionalizing Mo_2_CT*_x_* with layered double hydroxides (LDH) for energy storage applications.

### 4.3. Heteroatom Doped MXene-Based Supercapacitors

Doping is a widely used strategy to enhance the conductivity and capacitance of MXene heteroatom. Wen et al. reported that N-doping was a cost-effective method for enhancing the conductivity and capacitance of MXene. By annealing Ti_3_C_2_ in NH_4_ at different content levels, N-Ti_3_C_2_ was obtained with a capacitance that was 5.6 times larger than bulk Ti_3_C_2_ in 1 mol L^−1^ H_2_SO_4_ [115]. This shows that doping with heteroatoms, particularly nitrogen, is an effective strategy to improve the performance of MXene. Li et al. [116] prepared a binder-free supercapacitor electrode material by decorating N-doped superhydrophilic carbon cloth with Ti_3_C_2_T*_x_* nanosheets using electrophoretic deposition. The resulting Ti_3_C_2_T*_x_*/ENCC nanostructures exhibited an areal-specific capacitance of 2080 mF cm^−2^ at 1 mA cm^−2^, which combines pseudocapacitive and EDLC behavior. The symmetric device showed a capacitance retention of 91% over 10,000 cycles and demonstrated good performance in storage applications due to the interaction between Ti_3_C_2_T*_x_* and carbon cloth through hydrogen bonding. Liu et al. [117] reported a new method for synthesizing nitrogen-doped Ti_3_C_2_T*_x_* aerogels as high-performance supercapacitor electrodes. The method involves combining nitrogen doping with 3D structure building in a one-step hydrothermal reaction. The unique 3D structure of the aerogel has a surface area of 200.8 m^2^ g^−1^, which is nearly 25 times higher than that of a Ti_3_C_2_T*_x_* film, and the N- doping at the edge of Ti_3_C_2_T*_x_* produces more active sites. The 3D porous structure improves electronic double-layer capacitance, and the presence of N-C_4_ increases the electron mobility of Ti_3_C_2_T*_x_*, reducing charge transfer resistance. The N-doped Ti_3_C_2_T*_x_* aerogel electrode showed a specific capacitance of 531 F g^−1^ in a 3 mol L^−1^ H_2_SO_4_ electrolyte and maintained a capacity retention of 96% after 5000 cycles. The asymmetric device of N-doped Ti_3_C_2_T*_x_*-aerogel//AC demonstrated an ED of 21.7 Wh kg^−1^ and an excellent cycling stability of 85% over 5000 cycles. The authors believe that the N-doped Ti_3_C_2_T*_x_* aerogel has potential for use in storage and conversion devices.

Das et al. [118] utilized a combination of density functional theory (DFT) and a thermodynamic solvation model to examine the electrochemical behavior of doped and substituted Ti_3_C_2_T*_x_* (where T*_x_* represents the mixed functionality of the system). The findings indicate that the presence of -O and -OH groups has increased the redox activity for charge storage in Ti_3_C_2_T*_x_*, which evolves from Ti_3_C_2_O_2_ to Ti_3_C_2_(OH)_2_. The effect of nitrogen doping at three different sites in functionalized Ti_3_C_2_ was also analyzed to determine its impact on the total capacitance. In addition, the authors substituted 50% of the carbon with nitrogen atoms and 66% of the molybdenum in the outer titanium layer to create Ti_3_CNT*_x_* and Mo_2_TiC_2_T*_x_* systems, respectively. The reason behind these specific substitutions was that the ordered structures of these two systems had already been explored. The authors of this study resolved the contribution of the surface electrostatic double layer (EDL) and redox effects to the stored charge and capacitance in H_2_SO_4_ electrolytes through an implicit solvation model combined with density functional theory (DFT). The results reveal that nitrogen doping at different positions in Ti_3_C_2_T*_x_* leads to the largest capacitance gain of 380 F g^−1^, which is two times higher than that of bare Ti_3_C_2_T*_x_* due to the increased surface redox activity. The presence of nitrogen dopants on the surface and the maximum coverage of H^+^ ions in the electrolyte are responsible for the enhanced electrochemical behavior. The study shows that the surface redox activity dominates the electrochemical behavior in doped systems, but the EDL mechanism also contributes and competes with it. As the nitrogen content increases, the capacitance value decreases due to the displacement of carbon from the lattice sites. On the other hand, nitrogen substitution results in EDL evolution becoming the dominant mechanism at higher voltages, but poor charge transfer limits capacitance growth. Overall, this study highlights that nitrogen doping is a more effective strategy for improving the electrochemical activity of Ti_3_C_2_T*_x_* electrodes than nitrogen substitution.

Phosphorus doping was used to enhance the electrochemical performance of Ti_3_C_2_T*_x_* MXene [95,119,120,121,122]. Additionally, Liu et al. [121] showed that the P-doping effectively improved the conductivity and reduced the restacking of the MXene layers, resulting in a more stable and high-performing supercapacitor. The results demonstrated the potential of heteroatom doping, especially P-doping, in enhancing the electrochemical performance of MXene-based supercapacitors. P-doped Ti_3_C_2_T*_x_* was prepared using a facile annealing method and achieved a high capacitance of 31.11 mAh g^−1^ at 1 A g^−1^ in 1 mol L^−1^ KOH, with an excellent ED of 8.2 Wh L^−1^. Zhang et al. coupled red phosphorus nanodots with Ti_3_C_2_T*_x_* MXenes to improve the performance of Li-ion and Na-ion batteries with an initial capacity of 863.8 mAh g^−1^ at 50 mA g^−1^ [120]. Wen and coworkers developed P-doped Ti_3_C_2_T*_x_* nanosheets through annealing with sodium hypophosphate in H_2_SO_4_ electrolyte and showed a capacitance of 320 F g^−1^ at 0.5 A g^−1^ [122]. P-doping is challenging due to the larger covalent radius of P atoms with respect to C atoms in MXene, which can cause structural distortions, leading to more defects and exposing more reactive sites that negatively affect the electrochemical performance. Nevertheless, these studies demonstrate the effectiveness of phosphorus doping in enhancing the capacitance of MXene-based electrodes.

Yin et al. [123] developed a flexible and wearable supercapacitor with excellent mechanical deformation and ultra-low temperature tolerance. They used a combination of MXene/carboxymethyl cellulose (CMC) film as the flexible electrode and PVA/LiCl hydrogel as the electrolyte. The CMC effectively prevents self-weight stacking and forms strong hydrogen bonds with the MXene to combine high mechanical properties and electronic conductivity. The PVA/LiCl hydrogel electrolyte has high ionic conductivity, stretchability, skin-like elasticity, self-adhesion, self-healing, and frost resistance due to the modulation of interactions using the LiCl–OH of the PVA chains and the formation of a Li^+^(H_2_O)_n_ hydration structure with H_2_O. The resulting supercapacitor offered high specific capacitance and impressive capacitance stability even at ultra-low temperatures of −40 °C and under various mechanical deformations. Chen et al. [124] developed a bioinspired, robust composite film made of Ti_3_C_2_T*_x_* and hemicellulose. This film was created through a simple vacuum-assisted self-assembly process. Hemicellulose, composed of xylose units linked by β-1,4 glycosidic bonds, is embedded in the aligned Ti_3_C_2_T*_x_* nanosheets and held together by hydrogen bonds, resulting in a nanostructured film with improved mechanical strength. The embedding of hygroscopic hemicellulose also enhances the film’s humidity-responsive activity. The film offers a high mechanical strength of 125 MPa, high electrical conductivity of 6.43 × 10^3^ S m^−1^, and good flexibility with a gravimetric capacitance of 335 F g^−1^. Unlike other polymers, the short-chained hemicellulose allows for the tethering of Ti_3_C_2_T*_x_* nanosheets into a strong material without a significant amount of insulating phase.

Luo et al. [125] used a cross-section wood (CW) with good mechanical strength and flexibility to obtain a flexible electrode using Ti_3_C_2_T*_x_*. The authors first created abundant pores in the CW to allow it to absorb Ti_3_C_2_T*_x_* and exposed cellulose to form a stable combination with Ti_3_C_2_T*_x_*. They then applied cyclic pressure to form negative pressure, which pumped the Ti_3_C_2_T*_x_* suspension into the CW and triggered the layer-to-layer self-assembly of Ti_3_C_2_T*_x_* sheets onto the wood cell wall by evaporating the water in the suspension. With a large Ti_3_C_2_T*_x_* loading mass ratio, the resulting free-standing electrode was found to have good electrical conductivity and flexibility, with a capacitance of 805 mF cm^−2^ at 0.5 mA cm^−2^ and a capacity retention of 84% at 10 mA cm^−2^. Additionally, the device showed good flexibility, with a life of 90.5% after 10,000 cycles at a constant bending angle of 90°.

Chen et al. [126] deposited a large amount of Ti_3_C_2_ on natural wood silicon wafers using a modified drop-casting method to improve the electrical conductivity and electrochemical performance of the wood. The metal ions helped form a Ti_3_C_2_ airgel with abundant pores in the wood container, reconstructing the porous structure of the Ti_3_C_2_-coated wood (TW). This 3D conductive network across the entire wood surface increased the capacitance of the TWs by promoting more active sites and enhancing ion accessibility through hierarchical ion channels with the wood container. The resulting Ti_3_C_2_ air gel-deposited wood (ATW) showed the highest capacitance of 930 mF cm^−2^ at 0.5 mA cm^−2^, with a capacitance retention of 88.2% at 10 mA cm^−2^. A symmetric device assembled with lignosilica as a separator delivered 23 Wh cm^−2^ at 577 W cm^−2^ with 87% retention over 5000 cycles. At 0.5 mA cm^−2^, the electrode prepared through Ti_3_C_2_ airgel deposition on wood (ATW) improved the capacitance by 63% compared to wood (TW). The improvement is due to the following factors: (i) a 3D conductive network that accelerated electron transport, (ii) abundant active sites that enhanced capacitance, and (iii) layered channels with large surface area that enhance ion accessibility. This work presents a novel strategy for converting biomass into high-value-added stand-alone electrodes with low carbon emissions and energy consumption. Moreover, the efficient pore reconstruction method used in this work does not increase the overall volume of the porous biomass. Chen et al. [127] reported a new supercapacitor electrode made from non-carbonized wood, which offers good mechanical strength and high electrical conductivity. They used a self-assembly method to rapidly evaporate water in Ti_3_C_2_ suspensions, causing positively charged polydopamine microspheres to stick to the negatively charged Ti_3_C_2_ nanosheets. This resulted in a high loading capacity of Ti_3_C_2_ on the wood without causing self-recombination or volume expansion of the wood. The Ti_3_C_2_-rich wood electrode had a surface area of 124.1 m^2^ g^−1^, good mechanical strength, and high electrical conductivity of 6.1 MPa and was dimensionally customizable. The symmetric device assembled with the wood as the separator had a capacitance of 870 mF cm^−2^ at 1 mA cm^−2^ and an ED of 10.5 μWh cm^−2^ at a PD of 390 μW cm^−2^, with excellent rate performance and 93% retention rate over 10,000 cycles. This study provides a new method for utilizing non-conductive and electrochemically inactive biomass without carbonization.

Recently, the capacitance enhancement in functionalized MXene supercapacitors M_n+1_C_n_O_2_, M = Ti, V, Nb, Mo was theoretically explored [128]. The studies revealed three sources of capacitance and found that quantum capacitance plays a crucial role in total capacitance estimation. The authors concluded that bare and O-functionalized compounds have plausible capacitance minima, but surface passivation may limit the total capacitance value. The study also found that the transition metal components, selected from the 3d and 4d series, did not show a clear difference or trend in terms of capacitance. Nb_n+1_C_n_ was identified as a potential anode for supercapacitors due to its capacitance value close to that of other compounds.

### 4.4. Theoretical Calculations

Density functional theory (DFT) is used to investigate the structural, electrical, and optical properties of the pure and functionalized Ti_3_C_2_ monolayer [129]. The results illustrated that the pristine Ti_3_C_2_ MXene and terminated ones with halogen atoms are dynamically stable metals with no energy band gap. The calculation of the phonon band dispersion depicts that the surface terminated Ti_3_C_2_ by halides is the dynamically stable novel functionalized monolayer material. The electronic band structure and density of states investigations demonstrate that all terminated monolayer structures preserve the metallic nature of Ti_3_C_2_. Theoretical calculations deduced from the XPS analysis of Nb_2_CT*_x_* MXene powders [48] showed that three optimal configurations of the most stable functionalized Nb_2_CT*_x_* were determined for the adsorption of a single Li atom.

DFT was used to investigate the quantum capacitance (C_Q_) and surface storage charge (Q) of F-functionalized M_2_C (M = Sc, Ti, V, Cr, Zr, Nb, Mo, Hf, Ta, W) MXenes in aqueous and ionic/organic systems. Three possible configurations for F termination are explored, and the M-top configuration with F on top of the M atom is the most stable [130]. The same research group reported the theoretical investigation of the quantum capacitance (C_Q_) for transitional-metal (TM) doped MXenes as electrode materials in supercapacitors. C_Q_ and surface storage charge (Q) of 13 kinds of 3d, 4d, and 5d TM atoms and vacancy-doped Sc_2_CF_2_, named TM@PS and VS, were evaluated. The doping of 3d TM atoms can effectively modulate the magnetism of pristine Sc_2_CF_2_. C_Q_ and Q of Sc_2_CF_2_-based electrode materials are effectively improved, and the type of electrode materials is changed because of TM doping. For aqueous and ionic/organic systems, Mn@PS is an excellent anode material, while PS, VS, and Y@PS are more suitable for cathode materials of asymmetric supercapacitors. V@PS, Zr@PS, Nb@PS, Hf@PS, and Ta@PS are more suitable for anode materials in ionic/organic systems [131]. Lin et al. [132] reviewed the theoretical studies on the quantum capacitance of two-dimensional electrode materials for supercapacitors. The larger the specific surface area, the better the energy storage performance of the electrode material demonstrated.

Zhan et al. [133] summarized the progress of the computational work regarding the theoretical design of new MXene structures and predictions for energy applications, including their fundamental, energy storage, and catalytic properties. Ashton et al. [134] applied DFT to study the dependence of the thermodynamics stability of MXene with different terminal groups on their chemical composition and hydrogen chemical potential, which indicates that a majority of the MXene candidates are theoretically synthesizable. Chen et al. [135] studied the lithium storage on Ti_3_CN via first principles simulation and found that Li adsorption prefers the nitrogen site on the bare surface and the carbon site on the functionalized Ti_3_CN MXene surface (T=O, OH, F), initiating the investigation of the nitrogen-enriched MXene for battery applications. Recently, Bharti et al. [136] determined the quantum capacitance (QC) of pristine and functionalized molybdenum carbide and vanadium carbide MXenes using density functional theory to investigate their suitability as supercapacitor electrodes. The calculations are performed using Synopsys ATK package with PBE functional under generalized gradient approximation, keeping the energy cut off at 540 eV and k-point sampling at 12 × 12 × 1. The calculated QC at the Fermi level in the case of V_2_C and Mo_2_C exhibit extremely high values of 3465 and 3243 μF cm^−2^, respectively. Using DFT calculations, we show that, unlike insulating polymer binders, the surface groups of Ti_3_C_2_T*_x_* MXene bond to PANI with a significantly high binding energy (up to −2.11 eV) via a charge transfer mechanism. This is one of the key mechanisms to achieve a high electrochemical performance of the conducting polymer-based electrodes when MXene is used as a binder [137].

## 5. Different Types of Devices with MXenes

MXene-based electrodes have entered the fabrication of electrodes in the three different types of supercapacitors and have become increasingly popular in symmetric, asymmetric, and battery-type hybrid supercapacitors in the broad context of 2D materials-based flexible supercapacitors [138].

### 5.1. Symmetric Supercapacitor Devices

**Figure 7:**
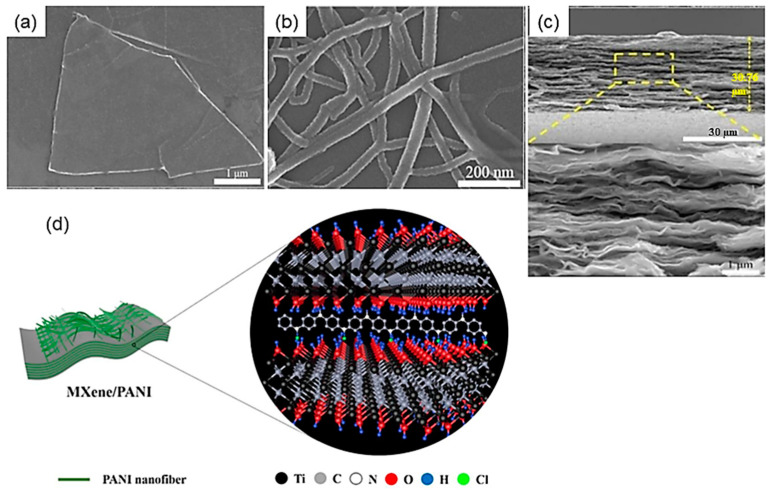
SEM images of MXene nanosheets (**a**), PANI nanofibers (**b**), and MXene/PANI films. Schematic diagram of binding mechanism between MXene nanosheets (**c**) and PANI nanofibers (**d**). Reproduced with permission from [139]. Copyright 2022 Elsevier.

Conductive polymers like polypyrrole (PPy), polyaniline (PANI), and polythiophene (PTh) are widely utilized as electrochemical energy materials. Among them, PANI is popular due to its high conductivity, large theoretical capacitance, easy synthesis, and low cost. Its charge storage mechanism is through pseudocapacitive doping and the redox reaction of PANI, where anions are transferred in and out of the electrode. PANI nanofibers hold great potential as they can be rapidly synthesized in water, resulting in porous structures and large specific surface area. Luo et al. [139] studied the thickness tuning of flexible Ti_3_C_2_T*_x_*/PANI hybrid materials used as high-performance supercapacitor electrodes. They achieved this by varying the concentration of PANI in the composite films. The synthetic route involved the minimum-strength hierarchical approach for the synthesis of MXene and a simple redox method for the preparation of PANI nanofibers. The MXene/PANI films were made by physical mixing and suction filtration. The symmetrical device showed a capacitance of 272.5 F g^−1^ at 1 A g^−1^ with a capacitance retention of 71.4% after 4000 cycles at 2 A g^−1^. The device had an ED of 31.18 Wh kg^−1^ at a PD of 1079.3 W kg^−1^, demonstrating its remarkable energy storage performance. Figure 7 represents the binding mechanism of MXene and doped PANI nanofibers. The MXene/PANI composite is a flexible electrode material with remarkable cycling stability and high specific capacitance, making it a promising material for the future fabrication of flexible all-solid-state supercapacitors. The main benefits of MXene/PANI hybrids are that (1) the interlayer spacing of MXene nanosheets can be enlarged by the addition of PANI nanofibers and (2) the electrical conductivity of the films increases, allowing for both electrolyte ion permeation and charge transfer. The increase in the PANI content results in an increase in the specific capacitance of the MXene/PANI hybrid for two reasons. First, the conductive PANI nanofibers facilitate the charge carrier path. Second, they increase the MXene layer spacing, which is beneficial for electrolyte ion transfer. Additionally, excess polyaniline leads to an increased film thickness, enhancing electron transport pathways.

Wu et al. [140] reported the creation of high-performance symmetric supercapacitors using a facile strategy that involves a one-step in situ polymerization and surface decoration of Ti_3_C_2_ nanosheets with polyaniline nanotubes (PANI-NTs). The hierarchical structure of the Ti_3_C_2_/PANI-NTs nanostructured electrode improved interlayer spacing and enlarged the ion contact area, allowing for fast electrolyte ion diffusion. The electrode showed a capacitance of 597 F g^−1^ at 0.1 A g^−1^ with a retention capacitance of 95% after 5000 cycles. The symmetric device had an ED of 25.6 Wh g^−1^ at PD of 153.2 W kg^−1^ and maintained 81.1% of its capacitance after 4000 cycles.

In recent years, planar supercapacitors with interdigitated electrodes have gained interest due to their flexibility, safety, and portability. Zhang et al. [141] developed planar supercapacitors with Ti_3_C_2_/polypyrrole interdigitated electrodes using electrophoretic deposition and electrochemical polymerization. The devices achieved areal capacitances of 109.4 mF cm^−2^ and 86.7 mF cm^−2^ in 2 mol L^−1^ H_2_SO_4_ and PVA/H_2_SO_4_ electrolytes, respectively. The planar supercapacitor showed good cycle stability with 96% capacitance retention after 10,000 cycles and an ED of 3.34 μWh cm^−2^ at a PD of 0.0884 mW cm^−2^. Polyphosphazenes, which consist of alternating phosphorus and nitrogen atoms in their structure, are a type of molecule that has shown potential for use in energy devices. Studies demonstrated their potential as flame retardants and catalysts due to their electron-rich system and stable covalent backbone [142,143]. In another study, Li et al. [144] reported the development of ring-crosslinked polyphosphazene-modified MXene for use in aqueous supercapacitors. The modification was achieved through nucleophilic addition and sequential condensation, resulting in Ti-O-P covalent bonds. This modification improved ion transport, accessibility, and oxidation resistance, leading to 380 F g^−1^ pseudocapacitance and excellent rate capability. The fabricated flexible symmetric supercapacitor achieved an ED of 12.26 Wh kg^−1^ at a PD of 125 W kg^−1^, demonstrating its potential for use in flexible and integrable energy devices. This study highlights the impact of surface modifiers on MXene properties and the potential of ring-crosslinked polyphosphazenes for modifying MXenes.

Currently, coplanar supercapacitors on textiles receive a lot of attention with the development of flexible and wearable electrochemical storage devices. Their configuration can significantly improve mechanical deformation and facilitate integration with other devices as compared with traditional stacked configurations. However, the low ED of such devices limits their applications. Zhang et al. [145] developed an in-plane hydric supercapacitor on textile, which enhances the energy density. They achieved this result by incorporating a battery-type electrode that combines the high capacity of metallic layer double hydroxide (NiCoAl-LDH) with Ti_3_C_2_T*_x_* and Ag nanowires. This combination showed the highest capacity of 592 C g^−1^ at 1 A g^−1^ with a good long cycle life of over 10,000 cycles. The device, when assembled on textile, showed a high areal ED of 22.18 Wh cm^−2^ at a PD of 3 mW cm^−2^ with remarkable bending capability, outperforming traditional carbon-based in-plane supercapacitors on textile. Chen et al. [146] developed flexible and mechanically stable in-plane flexible microsupercapacitors using a polymer-mediated Ti_3_C_2_T*_x_*/graphene framework. The poly(diallyldimethylammonium chloride)/Ti_3_C_2_T*_x_*/graphene nanostructured electrode showed a capacitance of 241 mF cm^−2^ and an ED of 12.05 Wh cm^−2^ with excellent cycling stability due to the presence of Ti_3_C_2_T*_x_* and the electrical conductivity of graphene. This technology allows the construction of large-area microscale electrochemical energy storage devices that are flexible and integrated. The authors demonstrated the ability to obtain arrays of strip-shaped micro-devices by simple dicing, which can power various commercial electronics like LEDs, Christmas trees, and electronic watches.

A nanostructure consisting of α-Ni(OH)_2_ and Ti_3_C_2_T*_x_* was assembled into flexible, all-solid-state hybrid supercapacitor devices [147]. The devices were composed of α-Ni(OH)_2_/Ti_3_C_2_T*_x_* and porous carbon electrodes, providing a high ED (29.3 Wh kg^−1^) at a PD of 800 W kg^−1^. The devices showed good mechanical flexibility and had a capacitance retention of over 90% after 5000 cycles with a gel electrolyte. In the study by Samal et al. [148], a bimetallic NiCoSe_2_/Ti_3_C_2_T*_x_* nanostructure was created using a hydrothermal method. This device showed an ED of 32.05 Wh kg^−1^ at a PD of 200 W kg^−1^ and maintained an ED of 15.39 Wh kg^−1^ at a PD of 920 W kg^−1^ with good flexibility. This remarkable performance was attributed to the charge transfer from MXene Ti_3_C_2_T*_x_* to NiCoSe_2_, leading to enhanced electronic states near the Fermi level, confirmed by DFT calculations. Xiang et al. [149] developed a ternary nanostructure of CNT/MXene/graphene (CMG) as a graphene-based composite fiber electrode for supercapacitors. The resulting CMG fiber showed high toughness, electrical conductivity, and electrochemical performance, thanks to the 3D cross-linked conducting network within graphene sheets achieved through covalent bonding and π-π interaction between acidified CNTs, graphene sheets, and MXenes. The optimized CMG fiber showed a high toughness of 1.7 MJ m^−3^ and electrical conductivity of 420 S cm^−1^, which was 4 times higher than the rGO fiber. The assembled fiber device delivered an aerial capacitance of 237 mF cm^−2^ at a current density of 0.1 mA cm^−2^, with 85% retention at 1 mA cm^−2^.

### 5.2. Asymmetric Supercapacitor Devices

**Figure 8:**
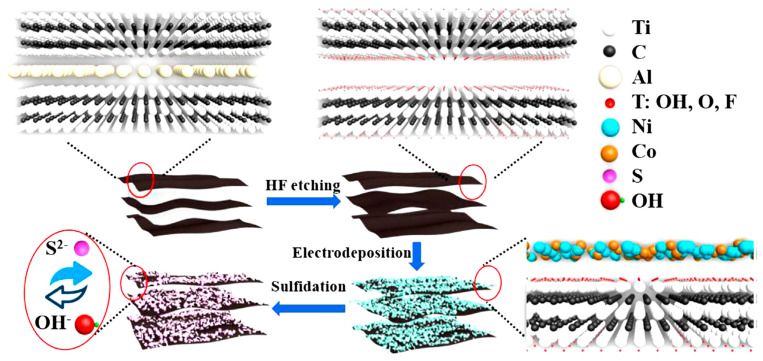
Schematic of the fabrication process for the MXene-NiCo_2_S_4_ electrode. Reproduced with permission from [150]. Copyright 2020 Elsevier.

Owing to high electrical conductivity, large surface area, excellent electrochemical performance, and good cycle stability, MXenes have been proposed as promising two-dimensional materials for supercapacitor electrodes. For example, in the work by Li et al. [150], a 3D hierarchical nanostructure was developed as a binder-free electrode for asymmetric solid-state supercapacitors by in situ depositing NiCo_2_S_4_ transition metal sulfide nanosheets on a conductive nickel foam surface of MXene (Figure 8). The resulting device exhibited high ED (27.24 Wh kg^−1^) and high PD (480 W kg^−1^) due to the combination of the favorable properties of both NiCo_2_S_4_ and MXene materials. The 3D nanostructure of MXene enhanced the electrochemical storage capacity and cycle stability due to its large surface area and ability to endure volumetric strain during charge and redox reactions.

Pathak et al. [151] developed a binder-free supercapacitor electrode using a single-step controlled electrodeposition method of spinel NiCo_2_S_4_ on various substrates like copper foil, nickel foam, and vertical graphene sheets grown on carbon ribbons (VG). The asymmetric device composed of Ti_3_C_2_T*_x_* as the anode and NiCo_2_S_4_ on Cu foil as the cathode showed the highest areal capacitance of 48.6 mF cm^−2^ and an ED of 14.86 Wh kg^−1^ at a PD of 8197 W kg^−1^ with 79% retention capability over 5000 cycles. The NiCo_2_S_4_ on the VG//Ti_3_C_2_T*_x_* device had better cycling stability of 85% over 5000 cycles due to the highly porous structure and multiple conductive networks of the VG substrate. This study highlights the potential of Ti_3_C_2_T*_x_* as a capacitive electrode to replace carbon-based electrodes in asymmetric memory devices and the hierarchical NiCo2S4 structure on various current collectors combined with Ti_3_C_2_T*_x_* as a promising candidate for electrochemical storage systems.

Hussein et al. [152] fabricated a porous WS_2_-embedded MXene/GO hybrid nanostructure as an electrode for asymmetric supercapacitor devices. The WS_2_/MXene/GO nanostructured electrode showed a specific capacitance of 1111 F g^−1^ at 2 A g^−1^, and the assembled asymmetric device had an ED of 114 Wh kg^−1^ at a PD of 1010 W kg^−1^. The good cycling stability was attributed to the porous MXene/GO structure acting as a spacer or charge collector and the layered gap-filling WS_2_ structure suppressing restacking/mixing and oxidation. The weak interaction between GO and MXene facilitated fast electron transfer, resulting in improved performance.

Venkateshalu et al. [153] investigated the use of Ti_3_C_2_T*_x_* MXene as the negative electrode and vanadium nitride/porous carbon as the positive electrode in an asymmetric supercapacitor device. The results showed that the asymmetric cell had a specific capacitance of 105 F g^−1^ at 1A g^−1^ in 6 mol L^−1^ KOH with a 73% capacitance retention after 10,000 cycles. The asymmetric device delivered an ED of 12.81 Wh kg^−1^ at a PD of 985.8 W kg^−1^ at 1 A g^−1^. This asymmetric cell exhibited a high potential window, about 3 times higher than the symmetric cell.

MXene, in conjunction with PPy, demonstrated exceptional performance as a pseudocapacitive electrode because MXene’s interlayered stacking prevents PPy agglomeration around it, while PPy’s intercalation prevents MXene’s dense stacking, resulting in high ion/electron transfer efficiency in MXene/PPy. MXene/PPy can be made via oxidative and oxidant-free in situ polymerization, electrochemical polymerization, and the solution mixing techniques reviewed in [154]. In particular, the conductive path structure of PPy supports supercapacitive properties, allowing it to store energy through electronic interactions [155]. During oxidation, electrons escape from PPy, and the matrix undergoes geometric deformation, making it ideal for electrochemical energy storage applications [156]. Its easy oxidation and high electrical conductivity make PPy a good candidate for use in energy storage applications. In the study by Vigneshwaran et al. [157], flexible quasi-solid-state supercapacitor electrodes were synthesized using a combination of MXene and PPy through an electrodeposition strategy. PPy was chosen as a material due to its high electrical conductivity, intrinsic redox activity, and stability in the doped state. The nanotubular flower-like morphology of the resulting MXene/PPy electrode effectively reduced the restacking of MXene layers and improved interlayer spacing. The asymmetric supercapacitor device MXene/PPy//activated carbon showed a maximum capacitance of 243 F g^−1^, cycling stability of 98% over 10,000 cycles, and an ED of 54.4 Wh kg^−1^ with a PD of 181.5 W kg^−1^ and a voltage window of 2 V. Liang et al. [158] constructed asymmetric supercapacitor devices using MXene-polypyrrole (PPy) as the negative electrode and PPy-multi-walled carbon tubes as the positive electrode. The MXene-PPy electrode was developed using an in situ polymerization method, which showed improved capacitance and lower resistance compared to previous methods. The improved performance was attributed to the in situ synthesis of PPy using PCV as a dispersant for MXene and an anionic dopant for PPy polymerization, which deposited conductive PCVs on the MXene surface and improved charge transfer. The asymmetric device exhibited comparable capacitances across complementary and overlapping potential ranges up to 1.7 V.

Padhy et al. [159] proposed a two-step strategy for the synthesis of boron-doped carbon/cobalt pyrophosphate nanostructures and optimized the concentration of boron during the synthesis reaction. The resulting material showed the highest capacitance of 395.1 F g^−1^ at 1.5 A g^−1^. When used as the cathode in an asymmetric device with Ti_3_C_2_T*_x_* as the anode, the device had a capacitance of 125 F g^−1^ and ED of 45 Wh kg^−1^ at a PD of 1735 W kg^−1^, with a capacitance retention rate of 96% and a Coulombic efficiency of 98.5% after 10,000 cycles. The authors attribute the improved electrochemical activity of the nanostructures to the presence of boron, which has fewer electrons than carbon.

### 5.3. Battery-like Supercapacitors

By definition, a battery-like supercapacitor uses a battery electrode and a supercapacitor electrode. These devices are attracting huge interest because of their superior energy density due to the intercalation of the metal ions within the electrode nanostructure for charge storage. The main difficulty, however, is the charge balancing of the two electrodes. MXenes-based electrodes have been used in these devices with success.

Nb_2_C, a 2D material belonging to the MXene family, has excellent metallic conductivity, surface chemical properties, and hydrophilicity [160], making it a promising candidate for electrode material. The interlayer Van der Waals forces in Nb_2_C result in prolonged ion transport channels, greatly affecting the overall electrochemical performance. Coupling nanoparticles or carbon-based materials between MXene layers efficiently avoids the self-accumulation of MXene layers. This strategy also increases the conductivity of the MXene surface and generates a huge quantity of uniformly distributed active sites. For example, Shen et al. [161] found that Nb_2_CT*_x_* MXene coated with Co_3_O_4_ forms a 2D cross-linked structure, and the self-assembly of Co_3_O_4_ between Nb_2_C MXene layers improves the conductivity and the number of active sites. The maximum capacitance of the Co_3_O_4_/Nb_2_C electrode was 1061 F g^−1^, which is higher than that of bulk Co_3_O_4_ and Nb_2_C electrodes. An asymmetric device of Co_3_O_4_/Nb_2_C//AC showed an ED of 60.3 Wh kg^−1^ at a PD of 670 W kg^−1^, with a retention of 93% over 1000 cycles at 5 A g^−1^.

MXene (Ti_3_C_2_T*_x_*) modified *α*-Co(OH)_2_ battery-type cathode and highly capacitive binder-free Ti_3_C_2_T*_x_* anode were assembled for the fabrication of high-performance electrochemical hybrid capacitor (EHC) [162]. Using a simple drop-casting method, the *α*-Co(OH)_2_ surface modified with 0.05 mg cm^−2^ Ti_3_C_2_T*_x_* MXene (CM0.05) shows the maximum specific capacity of 403 C g^−1^ at the current density of 3 A g^−1^. The aqueous EHC fabricated with CM0.05 as a positive electrode and two-dimensional (2D) Ti_3_C_2_T*_x_* MXene nanosheets as a negative electrode showed the maximum ED of 44.5 Wh kg^−1^ at the PD of 2762 W kg^−1^. It also showed an appreciable stability of 72% even after 5000 cycles.

### 5.4. Selection of Electrolytes

The electrolyte used in supercapacitors plays a critical role in determining their performance, including the energy and power density, charge-discharge rate, and cycle stability. The selection of the electrolytes is particularly important in battery-type devices because the electrolyte must be suited to both the supercapacitor electrode and the battery-type electrode. Otherwise, it leads to failure of the device. In general, the electrolytes used in supercapacitors can be divided into two categories: aqueous and non-aqueous. Aqueous electrolytes, such as H_2_SO_4_ and KOH, are relatively low-cost, environmentally friendly, and provide high conductivity. However, they also have low oxidative stability, which can result in the degradation of the electrodes and a reduction in the capacitance. To mitigate these issues, researchers have used ionic liquids or solid-state electrolytes as alternatives to aqueous electrolytes. Non-aqueous electrolytes, such as organic solvents (e.g., acetonitrile) or ionic liquids, have better oxidative stability than aqueous electrolytes, providing a longer cycle life. However, they are often more expensive and have lower conductivity compared to aqueous electrolytes. The choice of electrolyte can also affect the type of electrodes used in a supercapacitor. For example, aqueous electrolytes can lead to corrosion of metal electrodes, whereas non-aqueous electrolytes are often compatible with metal electrodes.

In a supercapacitor, the electrolyte ions diffuse into the electrodes and participate in the formation of an electric double layer, which is responsible for the storage of the electrical charge. This process can be described as follows: (i) when a voltage is applied across the electrodes, positive ions in the electrolyte are attracted to the negative electrode, and negative ions are attracted to the positive electrode, forming a layer of ions at each electrode surface. (ii) The charged electrodes create an electric field that repels additional ions from entering the electrodes. This electric field acts as a barrier, preventing further ion diffusion into the electrodes. (iii) The electric double layer formed at the electrode-electrolyte interface is responsible for the storage of electrical energy in a supercapacitor. Therefore, the electrolyte used in a supercapacitor plays a critical role in determining its performance, and further research is needed to optimize its choice.

Yu et al. [163] introduced a smart electrolyte based on a thermosensitive copolymer poly(N-isopropylacrylamide-glycidyl methacrylate) (PNGM) for high-performance Ti_3_C_2_T*_x_* symmetric supercapacitors. This PNGM-based electrolyte has a hydrophilic nature at room temperature, allowing for free movement of ions and electrons. However, at high temperatures, the collapse and shrinkage of copolymer chains results in the electrolyte transitioning to a hydrophobic state, reducing ion migration, and shutting down the electrochemical device, providing temperature-dependent protection against thermal runaway. The PNGM-based electrolyte overcomes the disadvantage of capacitance loss at high temperatures in previous PNIPAM-based electrolytes and is also economical. The smart electrolyte is stable in various electrolyte environments and effectively suppresses ion migration at high temperatures. The self-protected Ti_3_C_2_T*_x_* supercapacitor made with poly(N-isopropylacrylamide-co-vinylsulfonic acid) (PNGM) electrolyte has several advantages. Firstly, the PNGM electrolyte synthesized using a simple radical copolymerization method overcomes the disadvantage of low capacitance at high temperatures present in previous poly(N-isopropylacrylamide) (PNIPAM) based electrolytes. Additionally, PNGM is economical as it has a low monomer cost. Secondly, the smart electrolyte is designed to sensitively adjust the electrochemical performance at high temperatures, making it highly stable in electrochemical environments, even at temperatures up to 85 °C. Finally, the synthesized copolymer can be used in acidic, alkaline, and neutral electrolytes, effectively suppressing more than 90% of ion migration at high temperatures, which is a challenging feat to achieve with previously reported electrolyte systems. Indeed, the use of ionic liquid electrolytes with wider electrochemical windows has the potential to significantly improve the capacitance of supercapacitors. Ionic liquids are characterized by their high ionic conductivity, wide electrochemical stability windows, and non-volatility, making them attractive candidates as electrolytes in supercapacitors. The combination of Ti_3_C_2_T*_x_* and ionic liquids is particularly promising, as Ti_3_C_2_T*_x_* is a highly conductive 2D material with a high surface area, making it an excellent candidate for use as an electrode material in supercapacitors. The high ionic conductivity of the ionic liquid electrolyte and the high surface area and conductivity of Ti_3_C_2_T*_x_* provide the necessary conditions for high-capacitance supercapacitors. The use of ionic liquids in combination with Ti_3_C_2_T*_x_* has the potential to result in supercapacitors with improved capacitance, stability, and safety compared to conventional supercapacitors. Wang et al. [164] found that the combination of Ti_3_C_2_T*_x_* and ionic liquid electrolytes can have a positive impact on the capacitance of supercapacitors through molecular dynamics simulations. The study showed that the orientation of cations on the surface of the electrodes affects the electric double layer (EDL) structure and capacitance, with the Ti_3_C_2_(OH)_2_ electrode having 2 times higher capacitance than the Ti_3_C_2_O_2_ electrode. These authors also found that hydroxyl-functionalized ionic liquid electrolytes and Ti_3_C_2_T*_x_* electrodes with hydroxyl end groups have the potential to achieve high energy/power densities. Molecular dynamics simulations were used to investigate the effect of the termination and functionalization of Ti_3_C_2_T*_x_* electrodes and ionic liquids on the EDL structure. It is shown that the orientation of cations on the two Ti_3_C_2_T*_x_* electrodes was different, leading to different EDLs with varying capacitance. The analysis showed that the Ti_3_C_2_(OH)_2_ electrode had a higher capacitance compared to the Ti_3_C_2_O_2_ electrode, which was 2 times higher at 0–2 V. The use of hydroxyl-functionalized ionic liquid electrolytes and Ti_3_C_2_T*_x_* electrodes with OH^−^ end groups resulted in high energy/power densities, as the hydroxyl groups from the cationic tail of hydrogen hydride bond changed the ionic arrangement in the EDL.

To address the aforementioned issues, researchers are developing new hydrogel electrolytes with improved mechanical stability and exploring different strategies to enhance their mechanical robustness. These include incorporating nanofillers, such as graphene and clay, into the hydrogel matrix to improve its mechanical strength and incorporating crosslinking agents to increase the hydrogel’s resistance to mechanical stress. Additionally, new polymer architectures, such as interpenetrating polymer networks (IPN), are being explored as the way to increase the mechanical stability of hydrogel electrolytes. Despite these efforts, the development of mechanically stable hydrogel electrolytes for supercapacitors remains a challenge, and further research is needed in this area. Peng et al. [165] aimed to develop mechanically reliable, electrochemically active, and freeze-resistant supercapacitors. They used a double-network hydrogel electrolyte, which showed good ionic conductivity at room temperature and low temperatures (−20 °C), and combined it with Ti_3_C_2_T*_x_* thin-film electrodes and CNT thin-film current collectors. The resulting device showed a high capacitance of 297.1 mF cm^−3^ and ED of 14.76 Wh cm^−2^ with good cycling stability. It also showed good capacitance stability even under continuous mechanical stress. The authors suggest that these supercapacitors could be used in self-powered devices integrated with hydrogels and sensors to detect various human motions accurately. In Kamaja et al. [166], the electrochemical behavior of Ti_3_C_2_T*_x_* and δ-MnO_2_ electrodes was investigated in various electrolytes such as H_2_SO_4_, KOH, NaHSO_4_, Na_2_SO_4_, MgCl_2_, and LiCl. The results showed that highly acidic electrolytes like H_2_SO_4_ and NaHSO_4_ were not suitable for the δ-MnO_2_ electrodes as they dissolve in these electrolytes. The 1 mol L^−1^ KOH electrolyte was not suitable for the Ti_3_C_2_T*_x_* electrode due to its instability. The best results were observed with the neutral aqueous electrolyte Na_2_SO_4_, where the Ti_3_C_2_T*_x_*//δ-MnO_2_ device exhibited an ED of 8.2 Wh kg^−1^ at a PD of 400 W kg^−1^ and an operating voltage of 1.6 V, which was higher than that of the symmetrically fabricated Ti_3_C_2_T*_x_* and δ-MnO_2_ materials.

### 5.5. Flexible Pseudocapacitors

**Figure 9:**
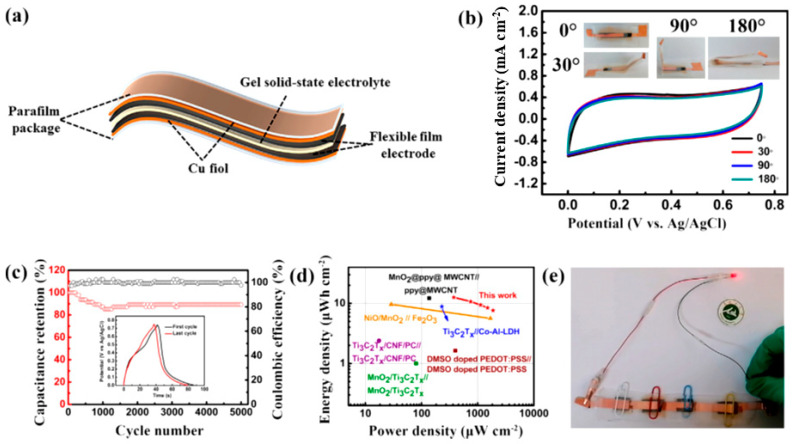
Electrochemical performances of Ti_3_C_2_T*_x_*-Ca-SA//Ti_3_C_2_T*_x_*-Ca-SA flexible all-solid-state symmetric pseudocapacitors. (**a**) Schematic illustration of assembled Ti_3_C_2_T*_x_*-Ca-SA//Ti_3_C_2_T*_x_*-Ca-SA flexible all-solid-state symmetric pseudocapacitors. (**b**) CV curves of the device at 10 mV s^−1^ under different bending angles. (**c**) Cycling stability and Coulombic efficiency of the device at 2 mA cm^−2^. (**d**) Ragone plots of our device in comparison to other flexible supercapacitors. (**e**) Digital photograph of four of our devices connected in series and powered by a red LED. Reproduced with permission from [167]. Copyright 2022 Elsevier.

In recent years, the advancement and development of flexible and free-standing MXene-based electrodes have been promising functional supercapacitors in wearable and portable electronics. However, the severe self-reorganization of MXene nanosheets limits their practical applications. To address this issue, Liu et al. [167] developed a flexible and free-standing pseudocapacitor electrode using 3D cross-linked Ti_3_C_2_T*_x_* and calcium-sodium alginate (Ca-SA) films. The films were made with an enlarged Ti_3_C_2_T*_x_* interlayer spacing, which allowed for more electrolyte ions to intercalate quickly, increasing pseudocapacitance. The 3D cross-linked microstructure facilitated charge transport and provided a continuous conductive network. The Ti_3_C_2_T*_x_*/Ca-SA film showed an excellent areal capacitance of 633 mF cm^−2^ at 5 mV s^−1^. The symmetric device of Ti_3_C_2_T*_x_*/Ca-SA had an energy density (ED) of 12.6 μWh cm^−2^ and power density (PD) of 375 μW cm^−2^ with excellent cycle retention. The role of Ca^2+^ ions in linking SA molecules for intercalation and triggering self-assembly between SA and Ti_3_C_2_T*_x_* nanosheets was also highlighted. This work demonstrates the potential of MXene-based electrodes as high-performance, flexible energy storage cells (Figure 9).

Guan et al. [168] studied the electrochemical performance of Ti_2_C flexible films in electrolytes, including 1 mol L^−1^ H_2_SO_4_, NaOH, and LiCl. They synthesized the Ti_2_C suspension by etching MAX with a mixture of LiF and HCl and then filtering the suspension to obtain a thin film. They found that the specific capacitance of the Ti_2_C thin films was high, reaching 382 F g^−1^ in 1 mol L^−1^ H_2_SO_4_ electrolyte against 100 and 98 F g^−1^ in LiCl and NaOH electrolytes, respectively, at 2 mV s^−1^. They attributed the electrochemical behavior of the Ti_2_C thin film to the coupling between the hydrated cations in the electrolyte and the surface termination of Ti_3_C_2_. Ning et al. [169] developed a flexible supercapacitor electrode consisting of MXene, carbon nanocoils, and poly(3,4-ethylenedioxythiophene): poly(styrenesulfonate) (PEDOT:PSS) using a simple drop-coating method. The carbon nanocoils served as highly conductive bridges that connect MXene nanosheets and 3D helical structures, intercalating the MXene layers to prevent restacking and creating spaces and channels for electrolyte storage and ion transport. The resulting 3D MXene/carbon nanocoils/PEDOT:PSS nanostructured flexible electrode showed an areal capacitance of 232 mF cm^−2^ at 10 mV s^−1^ and excellent cycling stability and flexibility. It also demonstrated a good energy storage capability and capacitive response under various deformations, providing a new strategy for constructing wearable, high-performance, flexible electrodes for energy storage applications. A flexible battery electrode was designed and constructed by covering carbon fibers with MXene before the addition of Fe_2_O_3_ via electrodeposition [170]. The hierarchical structure created by the combination of Ti_3_C_2_T*_x_* and Fe_2_O_3_ improved conductivity, ion diffusion, and electrochemical activity. The 3D MXene/Fe_2_O_3_ anode showed high capacity of 38.2 mAh cm^−3^. The Ni/Fe battery assembled with the NiCoO cathode had the highest volumetric capacity of 35.1 mAh cm^−3^ and good cycle stability of 93% after 12,500 cycles with good mechanical durability.

Cao et al. [171] developed a PANI@Ti_3_C_2_T*_x_*/PVA hydrogel composite as a flexible supercapacitor electrode using a sol-gel method followed by a freeze-drying process. The resulting electrode showed good electrochemical performance, with a capacitance of 103.8 mF cm^−2^ at 2 A cm^−2^, an ED of 9.2 μWh cm^−2^, and a power density (PD) of 800 μW cm^−2^. The flexibility of the electrode was attributed to the synergistic effect between PANI and Ti_3_C_2_T*_x_*/PVA, and it showed a good capacitance retention rate of 99% after 10,000 cycles. The presence of abundant active sites and effective open channels for ion diffusion and transport contributed to the good electrochemical performance of the PANI@Ti_3_C_2_T*_x_*/PVA hydrogel electrode. Qu et al. [172] fabricated a flexible asymmetric supercapacitor with a high energy density by designing a MXene (V_2_C) derived unique accordion-like layered vanadium nitride (VN) coated with an ultrathin amorphous carbon layer (VN@AC) as the negative electrode material. The VN@AC//α-MnO_2_ asymmetric supercapacitor showed a capacitance of 631.4 mF cm^−2^, excellent rate performance, and a high ED of 597.5 μWh cm^−2^ at PD of 2.4 mW cm^−2^. The asymmetric device showed good stability after being bent hundreds of times and was able to drive an electronic alarm clock for more than 10 min. The study provides insights into the field of energy storage from the perspective of electrode material design and device assembly.

Ka et al. [173] fabricated VSe_2_/Ti_3_C_2_T*_x_* nanostructures using a hydrothermal reaction. The VSe_2_ was decorated on the surface of Ti_3_C_2_T*_x_* nanosheets, and the resulting nanostructure showed a capacitance of 144 F g^−1^ at 1 A g^−1^. The improvement of the charge storage and capacitive behavior was attributed to the synergistic interaction between VSe_2_ and MXene. The study used DFT results to better understand the improved electronic properties and bonding of VSe_2_ and VSe_2_/Ti_3_C_2_T*_x_* nanostructures. The asymmetric device consisting of VSe_2_/Ti_3_C_2_T*_x_*//MoS_2_/MWCNT showed an ED of 42 Wh kg^−1^ at a PD of 4137 W kg^−1^ and maintained 90% of these values over 5000 cycles. Qi et al. [174] developed a stretchable supercapacitor using MXene electrodes with a thickness of 45 μm, gold foil with a thin anti-corrosion film as the current collector, and Ti_3_C_2_T*_x_* as the active material in a PVA-H_3_PO_4_ gel electrolyte. The electrodes showed a capacitance of 10.2 mF cm^−2^ at 5 mV s^−1^ with excellent mechanical flexibility. The stretchable supercapacitor was created by adhering ultrathin supercapacitors to pre-stretched latex substrates, which resulted in periodic wrinkles and led to stretchability up to 100% over 1000 cycles. The selective bonding of ultrathin supercapacitors to the substrate generated device-scaled wrinkles after the pre-strain was released. A flexible supercapacitor electrode was developed using Ti_3_C_2_T*_x_* and calcium alginate (CAC) [175]. The gelation of calcium alginate in the MXene nanosheets and the subsequent annealing process resulted in the creation of carbon dots embedded in the Ti_3_C_2_T*_x_* nanosheets, which increased the interlayer spacing and ion-accessible active surface area. The resulting Ti_3_C_2_T*_x_*/CAC film had a packing density of 3.3 g cm^−3^ and interlayer spacing of 1.37 nm, which contributed to its excellent bulk capacitive activity. The electrode had high gravimetric and volumetric capacitances (373 F g^−1^ and 1245 F cm^−3^) and maintained high capacitance even at high current densities (198.3 and 663 F cm^−3^ at 1000 A g^−1^). The Ti_3_C_2_T*_x_*/CAC film had a capacitance retention of 93.5% after 30,000 cycles, and the assembled device had a high gravimetric capacitance (912.1 F cm^−3^) and areal capacitance (2.73 F cm^−2^) at a mass loading of 10 mg cm^−2^. The peak capacitance was 27.2 Wh L^−1^ at a PD of 13,100 W L^−1^, which was attributed to its unique structure and the quantum effects of carbon dots. Luo et al. [176] addressed a method to create a micro-structured and flexible rGO/Ti_3_C_2_T*_x_* composite film electrode for supercapacitors by alternating filtration and reduction at low temperatures (up to 250 °C). The rGO and Ti_3_C_2_T*_x_* nanomaterials were cross-filtered to form a sandwich structure, and the reduction process increased the electrochemical performance of the film. The flexible film showed a capacitance of 322 F g^−1^ at 1 A g^−1^ in 3 mol L^−1^ H_2_SO_4_ and had good cycle stability after 32,000 cycles, with a capacitance retention of over 90%. The optimal weight percentage of GO was 20%. The thin-film electrode also showed good mechanical flexibility and performed well in neutral and alkaline electrolytes.

**Figure 10:**
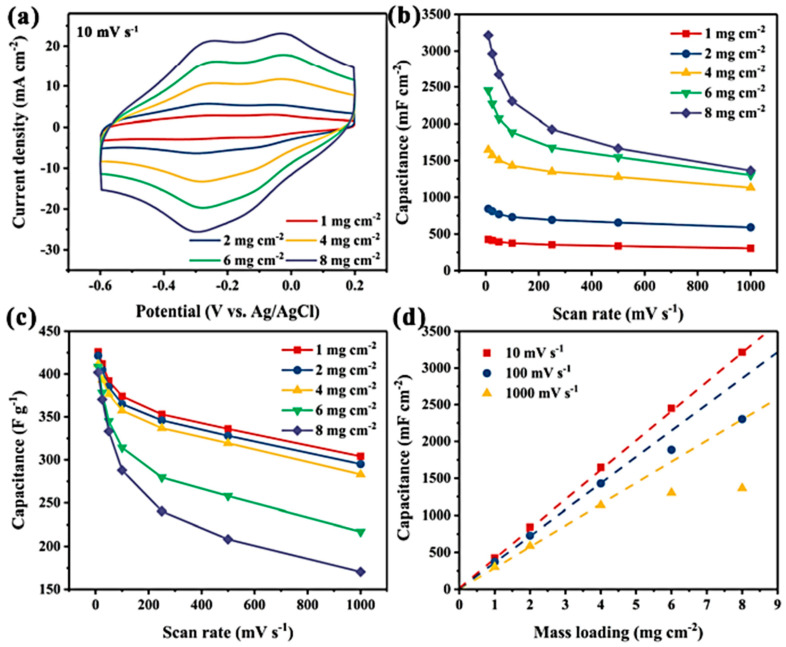
Electrochemical performance of Ti_3_C_2_T*_x_*@chitosan electrodes with mass loading varying from 1 to 8 mg cm^−2^ in 3 mol L^−1^ H_2_SO_4_ electrolyte. (**a**) CV curves at a scan rate of 10 mV s^−1^. (**b**) Areal capacitance and (**c**) gravimetric capacitance at scan rates varying from 10 to 1000 mV s^−1^. (**d**) A linear relationship was obtained between the areal capacitance and the electrode mass loadings. Reproduced with permission from [178]. Copyright 2022 Elsevier.

Zhang et al. [177] made a significant contribution to the field of energy storage by developing 2D/2D Co-doped NiMn-Layered double hydroxide (LDH)/V_2_CT*_x_* (CNMV) as advanced electrodes for aqueous energy storage devices. Their work demonstrates the benefits of hetero ion doping, leading to a capacitance of 1005 F g^−1^ at 1 A g^−1^ and an ED of 30.16 Wh kg^−1^ at a PD of 700 W kg^−1^ for the assembled CNMV//V_2_C asymmetric device. In the case of Zn-ion batteries, the Co-doped NiMn-LDH/V_2_CT*_x_* nanostructured electrode was used as the cathode and showed a reversible capacity of 323 mAh g^−1^ over 100 cycles at 0.2 A g^−1^, with an ED of 368.7 Wh kg^−1^ at a PD of 246 W kg^−1^. Through kinetic analysis, the authors demonstrated the transformation of the battery-type pseudocapacitive NiMn-LDH/V_2_CT*_x_* nanostructured electrode into a supercapacitor as a function of the scan rate. The phase transition and Zn^2+^ insertion/extraction mechanism of Zn ion storage are further explored through ex situ XRD and XPS analysis. This study provides a simple and effective strategy for designing efficient MXene-based electrodes for electrochemical storage applications, advancing the state of the art in this field. Ding et al. [178] presented a novel and innovative approach to constructing energy storage electrodes by utilizing a chitosan-supported self-assembly strategy to create flexible Ti_3_C_2_T*_x_*@chitosan films. The 3D ordered porous nanostructures in these films allow rapid transport channels for electrons and electrolyte ions, leading to an improved electrochemical performance at low mass loadings. The Ti_3_C_2_T*_x_*@chitosan thin film electrode showed a high capacitance of 245.2 F g^−1^ at a 2 V s^−1^ scan rate and a mass loading of 4 mg cm^−2^, with 57.1% capacitance retention at a 400-fold increase in scan rate. The interconnecting and interwoven cross-linked chitosan enhanced the mechanical strength and flexibility of the film. By assembling this thin film into a device, an ED of 143.2 Wh cm^−2^ and a PD of 6.44 mW cm^−2^ were achieved with good stability. This work presents a simple and effective method for assembling 2D Ti_3_C_2_T*_x_* into 3D flexible and porous films for use as advanced energy storage electrodes. Figure 10 displays the electrochemical performance of Ti_3_C_2_T*_x_*@chitosan electrodes with mass loading varying from 1 to 8 mg cm^−2^ in 3 mol L^−1^ H_2_SO_4_ electrolyte. This study demonstrates the potential of this approach to play a major role in the development of efficient energy storage devices.

Wu et al. [179] created hexagonal Ti_3_C_2_ thin films through a novel method combining Lewis acid molten salt and microwave-supported etching techniques. This approach improved the interplanar spacing of Ti_3_C_2_ and reduced layer stacking, resulting in a high capacitance retention of 97.37% after 10,000 cycles. The enhanced pseudocapacitance is attributed to an increase in the surface group −O of the Ti atom in hexagonal Ti_3_C_2_. The study provides new insights into developing long-cycle stable and flexible electrodes for energy storage applications. Zhao et al. [180] developed a flexible asymmetric supercapacitor device using NiAlP/NiAl-LDHs@Ti_3_C_2_T*_x_* nanostructures integrating layered double hydroxides (LDHs) and Ti_3_C_2_T*_x_* via hydrothermal method. The highest capacitance of the nanostructured electrode was 2589.3 F g^−1^, and it had a capacity retention of 91.1% after 8000 cycles. The flexible device had good structural integrity, stability of 96.9% over 10,000 cycles, and good flexibility, with an ED of 81.1 Wh kg^−1^ and a PD of 1.8 kW kg^−1^. The authors showed that the combination of NiAlP/NiAl-LDHs and Ti_3_C_2_T*_x_* materials creates numerous hierarchical channels and active sites, which contributes to the device’s good performance. The study provides a new idea for the optimal selection of electrode materials for flexible wearable electronics. Li et al. [21] investigated a hierarchical architecture of MXene/PANI hybrid electrodes for advanced asymmetric supercapacitors. The nanostructure was obtained through polyaniline (PANI) nanofibers and Ti_3_C_2_T*_x_* layers, which were integrated via a hydrothermal method. The optimal ratio of Ti_3_C_2_T*_x_*/PANI was found to be 1:3, which resulted in the highest capacity of 563 F g^−1^ at 0.5 A g^−1^, with a capacity retention rate of 95.15% after 10,000 cycles. The asymmetric supercapacitor based on Ti_3_C_2_T*_x_*/PANI delivered an ED of 22.67 Wh kg^−1^ and a PD of 217 W kg^−1^. The study showed that the hybrid electrode could improve the electrochemical characteristics of the supercapacitor due to the increased surface area and pseudocapacitive behavior of PANI.

**Figure 11:**
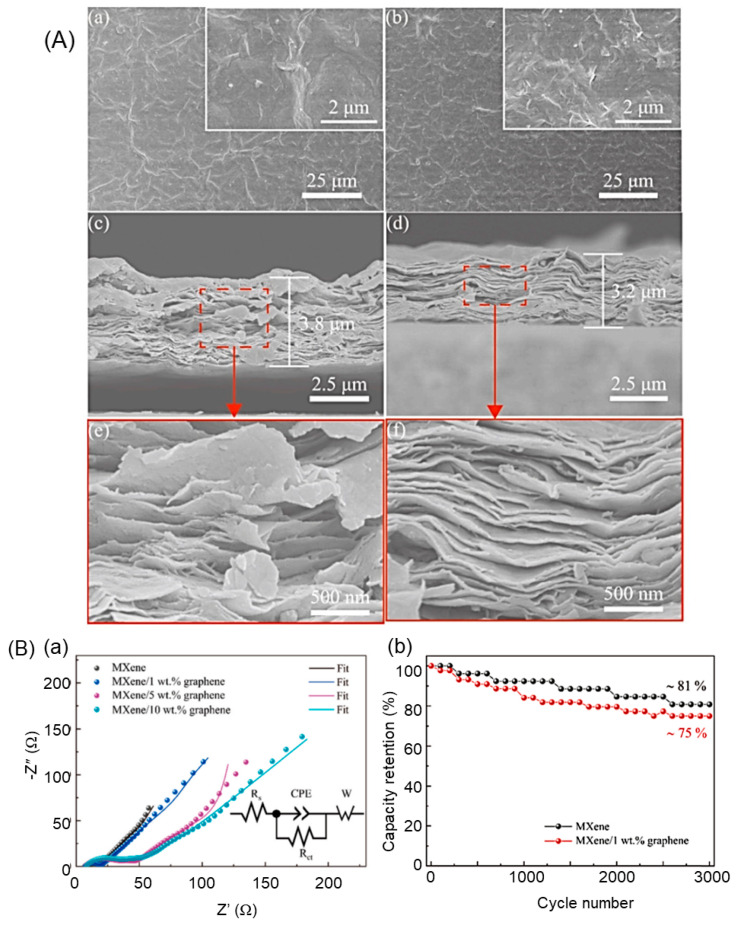
(**A**) The surface images of the printed (**a**) MXene and (**b**) MXene/10 wt.% graphene electrodes. The insets show the morphology at high magnification. The cross-sectional images of printed (**c**) MXene and (**d**) MXene/10 wt.% graphene electrodes, with (**e**) and (**f**) high-magnification SEM images, respectively. (**B**) Electrochemical performances. (**a**) Nyquist plots of different electrodes. (**b**) The cyclic stability of the printed electrodes. (Reproduced with permission from [181].

A flexible supercapacitor electrode was developed by combining Ti_3_C_2_T*_x_* and graphene through an inkjet printing process [181]. The interlayer spacing of the Ti_3_C_2_T*_x_*-based nanostructured films was improved by intercalating graphene sheets, which reduced the self-stacking problem of Ti_3_C_2_T*_x_*. The printed Ti_3_C_2_T*_x_*/graphene electrode exhibited high volumetric capacitance of 183.5 F cm^−2^ and good stability of 75% after 3000 cycles. The assembled symmetric flexible device had an ED of 0.53 Wh cm^−2^ and a PD of 10 W cm^−2^. Figure 11 presents the surface images and electrochemical performances of the printed MXene and MXene/10 wt.% graphene electrodes. This work provides a basis for the design and fabrication of flexible electronic energy storage devices.

A self-contained electrode composed of Ti_3_C_2_T*_x_*/rGO/Carbon (MGC-500) was prepared using a template method using melamine foam [182]. The MGC-500 nanostructure exhibited a gravimetric capacitance of 276 F g^−1^ at 0.5 A g^−1^. The symmetrical MGC-500 device assembled in PVA/H_2_SO_4_ nanostructured foam could be compressed into a flexible film and maintained its electrochemical stability under various bending and twisting conditions. It is considered a potential material for future wearable electronic applications. Wang et al. [183] developed a material that combines energy storage and actuation functions in a bilayer thin film made of MXene and biaxially oriented polypropylene (BOPP) films. The resulting device showed good flexibility and strong adhesion between the layers and could be actuated by electricity or near-infrared light. It has a bending curvature of 1.16 cm^−1^ when irradiated with 600 mW cm^−2^ near-infrared light for 5 s and can reach 1.51 cm^−1^ when driven by a voltage of 4 V. The areal capacitance of this electrode is 358.2 mF cm^−2^ at 0.1 mA cm^−2^. The authors demonstrated smart grippers, circuits, energy cells, and switches, all fabricated through the MXene/BOPP films. This material provides a material-for-all strategy and opens opportunities for further research into other materials. Chang et al. [184] developed a novel structured photothermal supercapacitor using 3D printed twisted Kelvin cell lattice arrays with a coating of graphene quantum dots (GQDs) and MXene nanohybrids. The nature-inspired spiral grass structure had a hierarchical design, ranging from macro to micro scale, that led to a strong electrochemical performance and exceptional photothermal-driven pseudo-capacitance improvement. The supercapacitor had a large electrochemical capacitance of 10.47 F cm^−2^, good light absorption and photothermal performance up to 67.6 °C, and a 3.04-fold increase in pseudocapacitance under one sun illumination. This novel structure exhibited excellent cycling stability and a record 1.18 mWh cm^−2^ ED at a PD of 223.58 mW cm^−2^ and cycling stability (10,000 cycles).

## 6. Metal-Ion Supercapacitors

### 6.1. Lithium-Ion Supercapacitors

Lithium-ion capacitors are a new type of energy storage device that combines the benefits of both lithium-ion batteries and supercapacitors. The cathode of a Li-ion capacitor is a high-surface-area capacitive material that can rapidly adsorb and desorb lithium ions, while the anode is a battery-type material with high lithium storage capacity based on a reversible Faradaic reaction. This combination of a high-capacity Faradaic anode and fast adsorption/desorption cathode allows for high power without sacrificing energy. Improving the Faradaic reaction kinetics at the anode to match the fast lithium-ion adsorption/desorption in the cathode is the key to obtaining high-power Li-ion capacitors. In recent years, these capacitors have received great success due to their high energy and power density. However, the main limiting factor in the performance of Li-ion capacitors is the mismatch of reaction kinetics caused by the differing energy storage mechanisms of the electrode materials. Yu et al. [185] found that SnO_2_/SnS_2_@MXene anode material shows excellent rate performance and increased lithium storage capacity, which is well-matched with the N-doped AC cathode. They reported that the use of a SnO_2_/SnS_2_@MXene hybrid anode material improved the performance of lithium-ion capacitors by regulating the charge transport at the interface between the cathode and anode and by enhancing electron conduction kinetics and ion diffusion. The asymmetric device consisting of this anode and a nitrogen-doped activated carbon cathode showed high ED and PD, long cycle life, and high-capacity retention over 2000 cycles. This work highlights the potential of SnO_2_/SnS_2_@MXene as a promising material for lithium-ion capacitors and how the structure and compositional design can influence their performance. The conductive MXenes help shield the bulk charge of SnO_2_/SnS_2_ hybrids and enhance the electrochemical behavior. The SnO_2_/SnS_2_@MXene electrode showed a capacity of 619 mAh g^−1^ after 200 cycles at 0.5 A g^−1^, indicating that it reduces the kinetic gap with capacitor-type cathodes. The asymmetric device with SnO_2_/SnS_2_@MXene anode and N-doped activated carbon cathode showed high energy and power density of 145.2 Wh kg^−1^ and 11,250 W kg^−1^, respectively, long cycle life and high-capacity retention (93.6% over 2000 cycles), making it a promising avenue for fast kinetics of Li-ion capacitor anode materials.

Lu et al. [186] synthesized SnO_2_@N-doped porous carbon (N-PC) as an anode material for lithium-ion capacitors using an electrostatic self-assembly strategy. The combination of SnO_2_@N-PC and MXene nanosheets resulted in a nanostructure with high capacity, reduced the restacking problem of MXene, and improved the Li+ transport kinetics. The SnO_2_@N-PC//MXene device showed a peak capacity of 465 mAh g^−1^ at 2 A g^−1^, a capacity retention of 90.2% after 500 cycles, and high PD and ED of 6097 W kg^−1^ and 56 Wh kg^−1^, respectively, with good cycling stability. The benefits of the electrode material were attributed to the embedded SnO_2_ nanoparticles, the N-PC matrix, and the flexible MXene nanosheets with high electrical conductivity and good mechanical strength. The SnO_2_@N-porous carbon has several advantages as an anode for lithium-ion capacitors. First, the embedded SnO_2_ nanoparticles contribute to high capacity. Second, the N-PC matrix confines the ultrafine SnO_2_ nanoparticles and can handle large volume expansion while avoiding the restacking problem of MXene, leading to improved capacity. Third, the flexible MXene nanosheets with good electrical conductivity and mechanical strength enhance the overall electrical conductivity and facilitate Li^+^ transport kinetics, reducing the negative effects of volume changes. Yang et al. [187] fabricated 3D Ti_3_C_2_T*_x_*-CNT structured electrodes to be used as anodes for Li-ion batteries. The electrodes are created through a simple and rapid gelation process by introducing Fe^2+^ ions and gas-phase carbon source acetylene (C_2_H_2_), followed by a CVD process to develop the 3D structure. The 3D feature provides a porous and multidimensional network structure, allowing for easier access to electrolyte ions and improving the ion/electron transport between interfaces. The 3D Ti_3_C_2_T*_x_*-CNT electrode has a capacity of 590 mAh g^−1^ and a good rate performance with a capacity of 191 mAh g^−1^ at 5 A g^−1^. When paired with a 3D N-doped framework-activated carbon as a cathode, the device exhibits an ED of 201 Wh kg^−1^ at a PD of 210 W kg^−1^ with 84.7% retention over 3500 cycles at 2A g^−1^. A free-standing VN/Ti_3_C_2_T*_x_* composite anode was developed for use in Li-ion hybrid capacitors by Guo et al. [188]. The anode showed a high capacity of 501.7 mAh g^−1^ at 0.1 A g^−1^, with a good rate capability of 191.8 mAh g^−1^ even at a high current density of 5 A g^−1^. The device assembled with the VN/Ti_3_C_2_T*_x_* anode and egg white-derived activated carbon cathode delivered a high ED of 129.3 Wh kg^−1^ at a PD of 450 W kg^−1^. The device showed excellent cycling stability, maintaining 98% capacity at 1 A g^−1^ after 5000 cycles, due to the improved electronic conductivity and Li^+^ diffusion network structure of the VN/Ti_3_C_2_T*_x_* electrodes and the capacitive behavior of the egg white-derived carbon, which accelerated the ion adsorption/desorption and enhanced both the capacity and the stability. Cho et al. [189] developed a flexible all-solid-state supercapacitor that outperforms most supercapacitors using novel structured electrodes. The electrodes were made by electrochemically polymerizing PEDOT and electro-spraying Ti_3_C_2_T*_x_* onto the PEDOT layer. The combination of the porous structure of electropolymerized PEDOT and crumpled Ti_3_C_2_T*_x_* flakes creates a highly porous and open structure that allows for easy filling of gel electrolytes, which results in large electrochemically active regions and fast ion diffusion paths. The symmetric device produced an ED of 20.65 Wh kg^−1^ and a PD of 45.45 kW kg^−1^. In addition, this preparation process of PEDOT/Ti_3_C_2_T*_x_* electrodes is simple, low-cost, and scalable. Jin et al. [190] developed a Li-ion capacitor consisting of MoS_2_/C@Ti_3_C_2_T*_x_* nanostructured anodes. This was achieved through growing uniform MoS_2_/C nanosheets on Ti_3_C_2_T*_x_* flakes using a hydrothermal process. The MoS_2_/C nanosheets enhanced the conductivity of MoS_2_ and alleviated the oxidation problem of Ti_3_C_2_T*_x_*, and the amorphous carbon matrix from diethylenediamine enhanced the conductivity. The MoS_2_/C@Ti_3_C_2_T*_x_* anode delivered a Li storage capacity of 600 mAh g^−1^ at 1 A g^−1^ with more than 700 cycles. The Li-ion capacitor device assembled with MoS_2_/C@Ti_3_C_2_T*_x_*//3D porous carbon as the cathode delivered an ED of 164.5 Wh kg^−1^ at 225 W kg^−1^ PD with 77.2% retention over 5000 cycles at 1 A g^−1^. The results suggest that optimizing the mass ratio of the two electrode active materials is a key factor for improving the electrochemical storage based on Li-ion capacitors.

**Figure 12:**
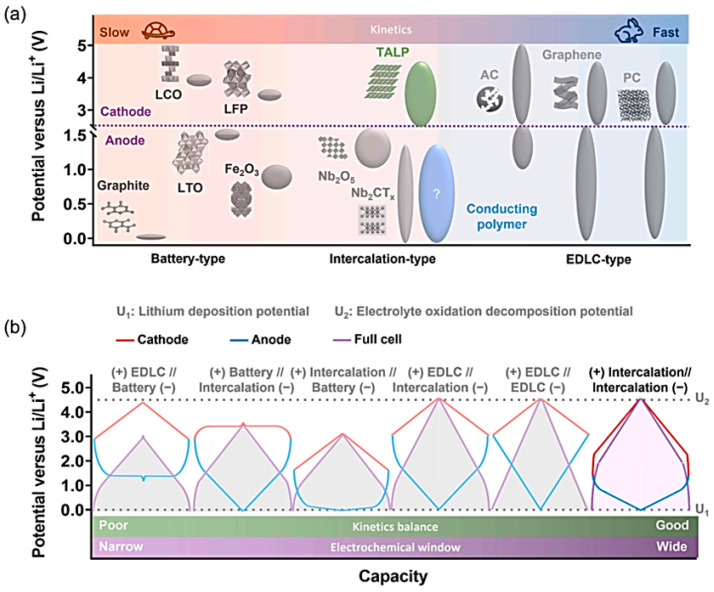
Illustration of the LISCs design. (**a**) Comparison of typical active materials in LISCs with different kinetics and electrode potential ranges, including tungstate anion linked polyaniline (TALP), Fe_2_O_3_, Li_4_Ti_5_O_12_ (LTO), Nb_2_O_5_, LiFePO_4_ (LFP), porous carbon (PC), Nb_2_CT*_x_* (MXene), graphene, graphite, activated carbon (AC) and LiCoO_2_ (LCO). Note that although carbon materials usually demonstrate EDLC behavior as cathode, they indeed undergo both EDLC and lattice insertion reactions when used on the anode. (**b**) Charge/discharge profile of LISCs with different configurations: (+) EDLC//battery (−) (AC//LTO); (+) battery//intercalation (−) (LFP//Nb_2_CT*_x_* MXene); (+) intercalation//battery (−) (Nb_2_CT*_x_* MXene//graphite); (+) EDLC//intercalation (−) (AC//Nb_2_CT*_x_* MXene); (+) EDLC//EDLC (−) (PC//PC); and (+) intercalation//intercalation (−). The intercalation here refers to only pseudocapacitive intercalation. U_1_ and U_2_ represent the lithium deposition potential and electrolyte oxidation decomposition potential, respectively. The kinetics balance between the cathode/anode and EW of the full cell are compared. Reproduced with permission from [192]. Copyright 2023 Elsevier.

Yu et al. [191] developed N-doped Ti_3_C_2_T*_x_*/TiO_2_ nanostructures using a one-pot hydrothermal method. The resulting structure integrated multiple physical and chemical benefits in a complementary and simple manner. The thin-film electrode showed a high capacitance of 918.7 F g^−1^ and excellent stability and flexibility, retaining 74.39% of its capacitance after 10,000 cycles at a current density of 2.0 mA cm^−2^ and remaining unchanged in capacitive performance when subjected to mechanical deformation. The proposed synthetic strategy provides a simple solution for the controlled hydrothermal modification of MXenes aiming to enhance electrochemical storage capacity as flexible electrodes. Liang et al. [192] developed a Li-ion capacitor using an anode made of electrochemically tuned layered polyaniline and solidified solid electrolyte interfacial nanochannels. The anode had a high volumetric capacity of 196 mAh cm^−3^ at 25 mA g^−1^ and a low potential of 0.01 V vs. Li^+^/Li. The operating voltage was 4.5 V with an ED of 100.5 Wh L^−1^ and a PD of 28 Wh L^−1^, and it maintained a stability of 90.1% over 10,000 cycles. The results showed that Li^+^ intercalated into the layered polyaniline through the solid electrolyte interface, forming a solid-solid ion adsorption interface between the interlayer and PANI nanosheets. The success of using low-potential conductive polymer anodes in this work could be extended to other batteries or capacitors by optimizing the active material morphology and integrating redox-active species in the polymer material (Figure 12).

It may be of interest to compare the performance of these MXene-based Li-ion capacitors with the more conventional Li-ion capacitors based on carbon. Yang et al. [193] developed Li-ion capacitors using hierarchical carbon nanotube arrays as the anode and activated carbon nanoparticle cathodes. The unique hierarchical structure of the carbon nanotube arrays enhances ion transport and Li^+^ storage, resulting in a high Li-ion storage capacity of 531 mAh g^−1^ at a current density of 0.5 A g^−1^ and a long cycle life of over 1000 cycles. The activated carbon nanoparticle cathode, due to its high surface area and fast ion diffusion kinetics, enhances the overall performance of the Li-ion capacitor, delivering an ED of 92.8 Wh kg^−1^ at a PD of 295 W kg^−1^. This study highlights the importance of optimized materials design and interface engineering to bridge the gap in capacity and electrochemical reaction kinetics between the anode and cathode in Li-ion capacitors.

### 6.2. Zinc-Ion Hybrid Supercapacitors

Lithium is a scarce and expensive element, leading to increased research in alternative energy storage devices. One alternative that has gained attention is the use of zinc in energy storage devices. Zinc is abundant, low-cost, and has a high theoretical capacity of 800 mAh g^−1^. Zinc-ion batteries have been shown to be highly reversible and have long life cycles, making them a promising alternative to traditional lithium-ion batteries. Zn is compatible with aqueous electrolytes, which are not flammable, but the potential window is lower than organic electrolytes and has a large theoretical capacity of 800 mAh g^−1^, −0.76V vs. SHE [194]. While Zn is widely used in primary batteries, commercial rechargeable zinc devices have been difficult to achieve because of dendrite formation with some electrolytes [195]. However, some neutral and moderately acidic aqueous electrolytes can support the reversible cycling of zinc for long periods of time. There have been limited studies on Zn-ion hybrid supercapacitors using Ti_3_C_2_ as the anode current collector and Zn nanosheets as the active cathode material, but commercially available Ti_3_C_2_ with high conductivity can store Zn ions reversibly. Zinc can be cycled for long periods of time without fading its capacity in some neutral and moderately acidic aqueous electrolytes with a pH between 4 and 6 in 1 mol L^−1^ ZnSO_4_. There have been a few reports of Zn-ion hybrid supercapacitors using Ti_3_C_2_ as the anode current collector and Zn nanosheets as the active cathode material [196]. A Ti_3_C_2_-based Zn-ion hybrid supercapacitor was developed by Yang et al. [197], who used the electrostatic adsorption of SO_4_^2−^ ions. Commercially available Ti_3_C_2_ can also reversibly store Zn ions. Etman et al. [198] developed a zinc-ion hybrid supercapacitor consisting of Mo_1.33_CT_z_-Ti_3_C_2_T_z_ hybrid MXene and Zn electrodes with aqueous and nonaqueous (acetonitrile-based) electrolytes. The study showed that the storage performance of the Zn-ion hybrid supercapacitor is affected by the characteristics of the halide supports, such as Cl^−^ and I^−^. The Mo_1.33_CT_z_-Ti_3_C_2_T_z_ hybrid MXene//Zn battery showed a capacity of 200 mAh g^−1^ and a capacity retention of 90% after 8000 cycles. The assembled hybrid MXene has energy densities of approximately 103 and 38 Wh kg^−1^ at power densities of 0.143 kW kg^−1^ and 10.6 kW kg^−1^, respectively. The use of Ti_3_C_2_T_z_ deposition on Zn foil surfaces was found to prevent passivation in ZnCl_2_ solutions and improve the Coulombic efficiency without affecting the accessible capacity or rate performance. In this Zn-ion hybrid supercapacitor, the Mo_1.33_CT_z_-Ti_3_C_2_T_z_ hybrid MXene and Zn were used as the cathode and anode, respectively. The use of aqueous electrolytes showed better performance compared to the acetonitrile-based electrolytes that have a low ionic conductivity. Engineering the Zn foil surface with Ti_3_C_2_T*_x_* deposition prevented passivation and improved the Coulombic efficiency of the Zn(CF_3_SO_3_)_2_ electrolyte. The Ti_3_C_2_T_z_ surface deposition layer also did not affect the accessible capacity or rate performance, making it a sustainable, low-cost option for Zn-ion hybrid supercapacitors.

Cui et al. [199] constructed a durable MXene-based zinc-ion hybrid supercapacitor using a N-doped Ti_3_C_2_T*_x_*-based structure wrapped with N-doped amorphous carbon. This structure was constructed through a template-guided approach and showed improved conductivity and electrochemical stability compared to conventional cathodes. Actually, the addition of nitrogen as a dopant to the Ti_3_C_2_T*_x_* matrix improves the conductivity of the material, making it easier for electrons to flow through it. The N-doped carbon layer also increases the stability of the material at anodic potentials, meaning it is less likely to react or break down chemically at high electrical potentials. This leads to a more efficient and stable material for use in energy storage devices like hybrid supercapacitors. A supercapacitor was made using the N-doped Ti_3_C_2_T*_x_* cathode and a soft conductive polymer hydrogel/electrolyte. The device had a high ED of 55 Wh kg^−1^ and a high PD of 3314.4 W kg^−1^ and showed a capacity retention of 96.4% after 10,000 cycles. Wang et al. [200] developed a Zn-ion capacitor made of Zn and V_3_CrC_3_T*_x_* material. The partial substitution of V atoms with Cr atoms improved the energy storage capacity by expanding the MXene interlayer spacing and increasing Zn ion adsorption and diffusion. The resulting micro-supercapacitor on paper showed a high capacitance of 1680.2 mF cm^−2^, an ED of 51.12 Wh cm^−2^, and a long cycle life of 20,000 cycles with 84.5% capacitance retention. The flexible properties and well-established synthesis process make it a promising candidate for flexible electronics. DFT studies confirmed that the introduction of Cr improved both the adsorption sites of Zn ions and the cycle stability and reduced the diffusion energy barrier.

A strategy was proposed by Li et al. [201] to improve the areal energy density for Zn-ion capacitors using polypyrrole-coated electrospun polyvinyl alcohol (PVA@PPy) nanofibers as intercalation spacers for Ti_3_C_2_T*_x_* electrodes. The PVA core provides optimal mechanical properties for interlayer space expansion, and the conductive PPy shell enhances the conductivity of the Ti_3_C_2_T*_x_* layers. By increasing the diameter of the PVA@PPy nanofibers instead of the loading of spacers, the Ti_3_C_2_T*_x_*/PVA@PPy electrode can enlarge the interlayer space and accelerate the Zn^2+^ charge diffusion transfer kinetics, leading to a 15-fold improvement in performance. The resulting electrode showed a maximum areal capacitance of 195 mF cm^−2^ and an ED of 38.4 Wh cm^−2^, with a capacitance retention of 80% and a Coulombic efficiency of 99% after 1000 cycles. Maughan et al. [202] used Ti_3_C_2_ as a reversible zinc-ion host in a hybrid supercapacitor. The addition of cetyltrimethylammonium bromide (CTAB) surfactant as a pillaring agent increased the zinc-ion uptake in the aqueous zinc sulfate electrolyte, resulting in a delivered capacity of 189 mAh g^−1^ and 96% cycling stability over 1000 cycles at 0.2 A g^−1^. This study showed that Zn^2+^ de-intercalation occurs through a combination of pseudocapacitive and battery-like behavior facilitated by the binding sites provided by the –O functional groups. This in situ pillaring approach can be applied to other 2D materials and has the potential for sustainable improvement in performance through the control of flake size, surface groups, and electrode structures, as well as the exploration of other multivalent energy storage systems. Peng et al. [203] aimed to improve the stability and zinc-ion storage capability of MXene (Ti_3_C_2_T*_x_*) electrodes. For this purpose, they manipulated the interlayer spacing of Ti_3_C_2_T*_x_* through the use of four diamine molecules of different sizes (ethylenediamine, 1,3-propylenediamine, 1,4-butylenediamine, and p-phenylenediamine) as pillars to cross-link Ti_3_C_2_T*_x_* and form a hydrogel. The enlarged interlayer spacing and 3D channel structure increased the electrolyte-accessible surface area and improved charge-transport properties, suppressing stacking and oxidation. The study shows that p-phenylenediamine-intercalated Ti_3_C_2_T*_x_* exhibits the highest capacitance (124.4 F g^−1^) at 0.2 A g^−1^, with 85% capacity retention after 10,000 cycles at 1 A g^−1^ and 100% Coulombic efficiency.

Yang et al. [204] developed a 3D macroporous Ti_3_C_2_T*_x_*/rGO/CNT nanostructured hydrogel with antioxidant properties using a mild gel method with L-cysteine as a cross-linker and L-ascorbic acid as a reducing agent. The cross-linking of the rGO and Ti_3_C_2_T*_x_* nanosheets formed a 3D macroporous nanostructured hydrogel composed of rGO and Ti_3_C_2_T*_x_* nanosheets, while *L*-ascorbic acid prevented the oxidation of Ti_3_C_2_T*_x_*. CNTs were added to improve conductivity. The cross-linking formed strong bonds between the –OH groups on the Ti_3_C_2_T*_x_* or RGO surface and the –NH_2_, –SH groups of *L*-cysteine. As a result, restacking of the nanosheets was effectively suppressed, and the utilization of active sites was increased. In addition, it exhibited good antioxidant activity with no significant change in electrical conductivity after storage. The electrode had a high capacitance of 349 F g^−1^, a rate capability of 52% at 3000 mV s^−1^, and a capacitance retention of 97.1% after 100,000 cycles at 200 mV s^−1^. The device had an ED of 28.8 Wh kg^−1^ with good cycling stability.

Shi et al. [205] developed a flexible electrode for zinc-ion capacitors, made of a battery-type cathode consisting of δ-MnO_2_ on carbon cloth and a capacitor-type anode made of Ti_3_C_2_T*_x_* on cotton cloth. The device achieved a high ED of 90 Wh kg^−1^ at a PD of 239 W kg^−1^, with 81% capacitance retention and 93.6% Coulombic efficiency after 16,000 cycles. The device showed excellent electrochemical activity and flexibility when assembled with an aqueous gel electrolyte and a maximum voltage of 1.9 V. This study gives evidence that zinc-ion capacitors have the potential to develop flexible energy storage devices with good cycling stability.

Zhang et al. [206] developed a new electrode material for aqueous Zn-ion hybrid supercapacitors composed of Ti_3_C_2_T*_x_* integrated with nitrogen and carbon-doped Bi_2_S_3_. The material was created through an in situ growth process and showed a capacitance of 653 F g^−1^. The Ti_3_C_2_T*_x_*/Bi_2_S_3_@@N-C device showed a high ED of 47 Wh kg^−1^ and PD of 750 W kg^−1^, as well as a high retention rate of 85.71% after 2000 cycles. The success of the material was attributed to the unique structural features of the multicomponent materials and the synergistic effect of multiple components, such as the highly conductive Ti_3_C_2_T*_x_* and the in situ growth of Bi_2_S_3_ on the Ti_3_C_2_T*_x_* nanosheets. The highly conductive Ti_3_C_2_T*_x_* shortens the electron transport path, the in situ growth of Bi_2_S_3_ on Ti_3_C_2_T*_x_* nanosheets takes advantage of Bi_2_S_3_ active sites, and the introduction of dopamine leads to uniform dispersion and improved close contact between the components. The results show that in situ growth for multicomponent nanostructures provides a promising avenue for developing efficient electrode materials.

**Figure 13:**
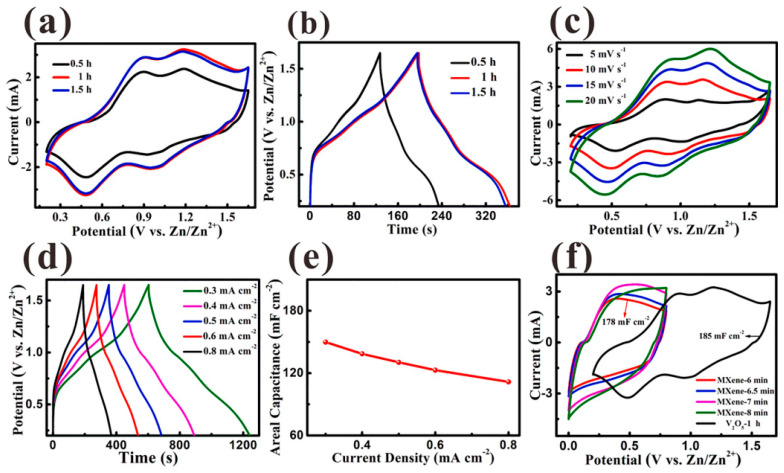
Electrochemical characteristics of the V_2_O_5_ cathode and Ti_3_C_2_T*_x_* anode in aqueous solution electrolyte. (**a**) The CV profiles of the V_2_O_5_ cathode with various electrochemical deposition times. (**b**) The GCD profiles of the V_2_O_5_ cathode with various electrochemical deposition times. (**c**) The CV profiles of optimized V_2_O_5_ cathode with a deposition time of 1 h at 5–20 mV s^−1^ scan rates. (**d**) The GCD profiles of optimized V_2_O_5_ cathode with a deposition time of 1 h under 0.3–0.8 mA cm^−2^ current densities. (**e**) The areal capacitance of the optimized V_2_O_5_ cathode. (**f**) The CV profiles of the Ti_3_C_2_T*_x_* MXene anode (various electrophoresis deposition times with 6–8 min) and optimized V_2_O_5_ cathode at 10 mV s^−1^ with a 1 h deposition time. Reproduced with permission from [208]. Copyright 2022 Elsevier.

In general, metal-ion supercapacitors, including Na-ion and Li-ion SCs, have the hidden danger of using organic electrolytes, which limits their practical applications [207,208,209,210,211,212,213]. Zn-ion capacitors avoiding this drawback have received the attention of many researchers. Wang et al. [207] developed a novel flexible zinc-ion capacitor based on free-standing thin films consisting of reduced graphene oxide (rGO)-V_2_O_5_ battery-type cathode and rGO-Ti_3_C_2_T*_x_* capacitor-type anode in an aqueous electrolyte. The configuration of this electrode system includes a specific layered structure, short ion diffusion paths, structural stability, and good electrical conductivity. The assembled rGO-V_2_O_5_//rGO-Ti_3_C_2_T*_x_* device delivered a high capacitance of 175 F g^−1^ at 0.5 mV s^−1^, an ED of 107.2 Wh kg^−1^ at a PD of 321.6 W kg^−1^, and maintained its capacitance up to 81% after 10,000 cycles. This capacitor has several advantages over other types of capacitors. (i) The use of aqueous liquid or gel electrolytes eliminates the safety hazards associated with toxic, flammable, or corrosive electrolytes. (ii) Reduced graphene oxide (rGO) has good electrical conductivity, which improves electrical storage; and (iii) the rGO-Ti_3_C_2_T*_x_* anode possesses both double-layer and ion intercalation/de-intercalation storage mechanisms, resulting in a high storage capacity. This novel electrode configuration results in Zn-ion capacitors with high storage capacity. Li et al. [208] developed a flexible Zn-ion hybrid micro-supercapacitor consisting of Ti_3_C_2_T*_x_* as a capacitive material and V_2_O_5_ as a battery-type cathode, integrated in a polyacrylamide hydrogel immersed in ZnSO_4_ electrolyte. This flexible micro-supercapacitor exhibited a 129 mF cm^−2^ capacitance at 0.34 mA cm^−2^, an ED of 48.9 Wh cm^−2^ at a PD of 673 W cm^−2^, and a capacitance retention rate of 77% (Figure 13). This hybrid micro-supercapacitor has potential applications in the field of microdevice storage, such as driving thermometers and LEDs. Unlike other hybrid supercapacitors in organic electrolytes, this Zn-ion hybrid micro-supercapacitor avoids safety risks.

Chen et al. [209] elaborated a hierarchical Ti_3_C_2_T*_x_* flake composite film that was functionalized through alkalization and post-annealing treatment to reduce the amount of −F and −OH functional groups. The resulting modified Ti_3_C_2_T*_x_* was combined with soybean straw-derived nanofiber cellulose to improve its mechanical properties, prevent density packing of Ti_3_C_2_T*_x_*, and provide efficient ion transport. This functionalized nanostructured film showed a high strength of 54 MPa and a high conductivity of 24,930 S m^−1^. The film had a high capacitance of 303 F g^−1^ at 1 mA cm^−2^ and 211.4 F g^−1^ at 10 mA cm^−2^, with a capacitance retention rate of 93% after 10,000 cycles. The film also demonstrated good resistance to bending deformation. Li et al. [210] developed a rechargeable aqueous zinc-ion hybrid capacitor with a 3D porous H-MXene film as the cathode. This cathode was created through cryo-casting, where H^+^ was introduced to weaken the MXene interlayer electrostatic repulsion. This quasi-solid-state Zn-ion capacitor delivered a capacitance of 106 mAh g^−1^ at 0.2 A g^−1^ and a good rate capability of 61 mAh g^−1^ at 5 A g^−1^ with excellent cycling stability over 20,000 cycles and 90% retention. It also exhibited good resistance to self-discharge, with a self-discharge rate of 1.87 mV h^−1^ and good flexibility.

Compared to Ti_3_C_2_T*_x_*, V_2_CT*_x_* MXenes have a higher energy storage capacity due to their small number of atomic layers and the different valence states of vanadium. Zhao et al. [211] investigated a flexible zinc-ion hybrid capacitor system developed using V_2_CT*_x_* MXene electrodes with a different number of layers. The electrophoretic deposition method was used to deposit single-layer, multi-layer, and few-layer V_2_CT*_x_* electrodes. The few-layer V_2_CT*_x_* electrode exhibited an areal capacitance of 54.12 mF cm^−2^ at 0.1 mA cm^−2^, which is higher than that of other V_2_CT*_x_*-based Zn-ion capacitors due to its reactive sites and shorter ion transport paths. The V_2_CT*_x_*-based Zn-ion capacitor also had good cycle stability, retaining 81.48% of its capacitance after 8000 cycles at 1 mA cm^−2^ and a low self-discharge rate of 6.4 mV h^−1^. The Ti_3_C_2_T*_x_*-based pressure sensor was integrated with the V_2_CT*_x_*-based flexible Zn-ion capacitors into a system that can continuously power the pressure sensor for 10 h and monitor various human activities. Table 1 lists the characteristics of MXene electrode materials for zinc-ion supercapacitors.

Ping et al. [212] developed a method of using Al^3+^ as a dynamic acid-tuned column to improve the capacity of Ti_3_C_2_T*_x_* Zn-ion capacitors. The method combines expanding interlayer spacing, tunable electrolyte acid, and pseudocapacitance. The resulting capacitors had a capacity of 278 F g^−1^, an energy density of 37.7 Wh kg^−1^, and good cycle stability with 94% capacitance retention after 10,000 cycles at 8 A g^−1^. The improved performance was explained using DFT calculations that confirmed the advantage of Al^3+^ as a dynamic pillar that can effectively expand the layer spacing of MXene. This study provides a way to control ion selectivity in MXene energy storage and can be applied to other electrolyte systems and aqueous Zn-ion capacitors. A new type of high-entropy MXene material (Ti_1.1_V_0.7_Cr_x_Nb_1.0_Ta_0.6_C_3_T_z_) was developed by Etman et al. [213] and used as electrodes in Zn-ion capacitors and lithium batteries. The free-standing films of the high-entropy MXene demonstrated good performance as an electrode, with a capacity of 77 mAh g^−1^ at 0.5 A g^−1^ and 43 mAh g^−1^ at 10 A g^−1^ and a capacity retention of 87% after 10,000 cycles in Zn-ion capacitors. When used as a lithium battery anode, the high-entropy MXene delivered capacities of 126 mAh g^−1^ at 0.01 A g^−1^ and 48 mAh g^−1^ at 2 A g^−1^ and showed a cycling stability of up to 10,000 cycles at 1 A g^−1^.

### 6.3. Sodium-Ion Supercapacitors

**Figure 14:**
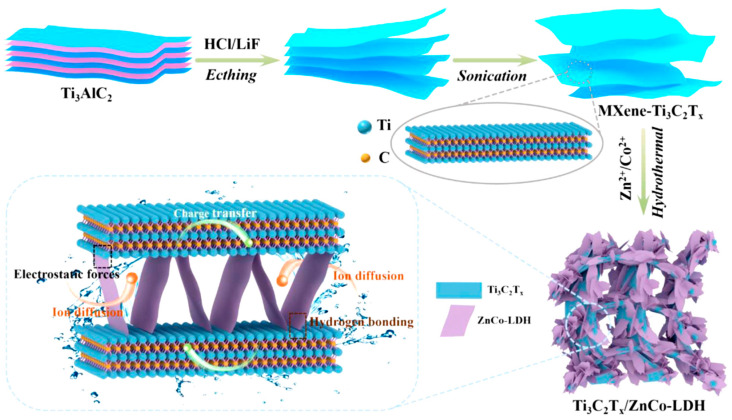
Schematic illustration of the synthesis and mechanism of energy storage for Ti_3_C_2_T*_x_*/ZnCo-LDH. Reproduced with permission from [215]. Copyright 2022 Elsevier.

Na-ion capacitors have been explored as a potential alternative to conventional batteries and capacitors due to the abundance of sodium compared to lithium [214]. However, finding electrode materials with a high performance and long lifetime for Na-ion capacitors remains a challenge due to the complexity of the synthesis process and the unpredictable nature of their structures. There is a need for further research to address these challenges and improve the performance of Na-ion capacitors. Layered double hydroxides (LDHs) have been considered promising electrode materials for Na-ion capacitors due to their high surface area and fast redox reactions. To enhance the electrochemical performance, researchers have coupled LDHs with MXene materials. Wang et al. [215] developed a 3D porous hybrid structure of Ti_3_C_2_T*_x_*/ZnCo-LDH for use as electrodes in sodium-ion capacitors (Figure 14). The hybrid structure improved the active sites for adsorbing charges, reduced agglomeration of Ti_3_C_2_T*_x_*, and facilitated electron transport kinetics, resulting in an excellent specific capacitance of 645 F g^−1^, pseudocapacitance, low resistance, and robustness (95.5% of initial capacitance after 10,000 cycles). The hybrid structure delivered an ED of 27.2 Wh kg^−1^ and a PD of 7987.5 W kg^−1^ with a stability of 95.8% after 10,000 cycles.

Lee et al. [216] developed a Na-ion capacitor using a NaTi_2_(PO_4_)_3_/Ti_3_C_2_T*_x_* nanostructure as a battery-type anode material that was synthesized in situ. This structure showed the highest capacity of 216 mAh g^−1^ at 0.1 A g^−1^, 73% of which was maintained at 2 A g^−1^. The combination of pseudocapacitive properties of Ti_3_C_2_T*_x_* and battery properties of NaTi_2_(PO_4_)_3_ led to better rate performance and cycle stability compared to the pristine nanostructure. The NaTi_2_(PO_4_)_3_/Ti_3_C_2_T*_x_*//AC assembly provided the highest ED of 112.1 Wh kg^−1^ and a PD of 9500 W kg^−1^. The in-situ growth of NaTi_2_(PO_4_)_3_ on the surface of Ti_3_C_2_T*_x_*, along with the conductive network of both materials, contributed to enhanced electrochemical capacitance. The pseudocapacitive properties of Ti_3_C_2_T*_x_* also allowed for a higher capacity and longer cycle life due to the adsorption/desorption of Na^+^ ions. Zhang et al. [217] developed a stretchable sodium-ion capacitor with higher scalability and a 3 V operating voltage. The elastomeric nanostructured electrode consisted of a 3D electrospun polyurethane fiber mat and a conductive 1D Ag nanowire network, with 2D MoSe_2_/Ti_3_C_2_T*_x_* and 3D AC/Ti_3_C_2_T*_x_* as the anode and cathode materials, respectively, embedded in the mat. The pre-straining of the fiber mat increased the relative stretching distance before losing the conductive connection between the layered components. The device delivered a capacity of 420 mAh g^−1^ at 5 A g^−1^, had good flexibility and stretchability, retained 95% after 1000 cycles at 2 A g^−1^, and the assembled asymmetric device delivered an ED of 5.44 mWh cm^−3^ at 493.55 mW cm^−3^. Chen et al. [218] developed 2D CuSe nanosheet electrodes for Na-ion capacitors through a simple hydrothermal reaction. The CuSe electrode had an initial Coulombic efficiency of 96.7% at 0.1 A g^−1^ and a capacity of 330 mAh g^−1^ after 100 cycles and maintained a capacity of 236 mAh g^−1^ after 3300 cycles at 5 A g^−1^ with a retention rate of 91.2%. The assembled Na-ion capacitor with CuSe as the anode and Ti_3_C_2_T*_x_* as the cathode showed an ED of 63.4 Wh kg^−1^ at a PD of 459.1 W kg^−1^, an operating voltage of 0–3.3 V and a capacitance retention rate of 77.7% after 2000 cycles at 2 A g^−1^.

### 6.4. Aluminium-Ion Capacitors (AISCs)

Aluminum ion capacitors, also known as aluminum capacitors or Al-ion capacitors, are a type of energy storage device that uses aluminum ions as the active material. They are similar to conventional capacitors in that they store energy in an electrical field but offer several advantages over traditional capacitors.

One of the key advantages of aluminum ion capacitors is their high energy density. They have been shown to have energy densities of up to 10 times higher than conventional capacitors, making them ideal for applications where space is limited and high energy density is required. This high energy density is achieved due to the high capacitance of aluminum ions and the efficient ion transport through the electrolyte. In addition to high energy density, aluminum ion capacitors are known for their long cycle life. Unlike batteries, which degrade over time and eventually need to be replaced, aluminum ion capacitors are designed to last for many thousands of charge/discharge cycles, making them ideal for applications where reliability is critical. This long cycle life is due to the stability of the aluminum ions and the electrolyte, which allows for repeated charge/discharge cycles without degradation. Another important advantage of aluminum ion capacitors is their fast charge/discharge rates. They can store and release energy very quickly, making them ideal for applications that require high-power output, such as in power-assisted bicycles or electric vehicles. This fast charge/discharge rate is due to the high ionic conductivity of the electrolyte and the efficient transport of aluminum ions between the electrodes.

Another advantage of aluminum ion capacitors is their long cycle life. Unlike batteries, which degrade over time and eventually need to be replaced, aluminum ion capacitors are designed to last for many thousands of charge/discharge cycles. This makes them ideal for applications where reliability is critical, such as in backup power systems for critical infrastructure. In addition to their high energy density and long cycle life, aluminum ion capacitors are also known for their rapid charge/discharge rates. This means that they can store and release energy very quickly, making them ideal for applications that require high-power output, such as in power-assisted bicycles or electric vehicles.

Despite these advantages, there are still several challenges that need to be overcome before aluminum ion capacitors can become a commercial reality. One of the main challenges is the development of materials that can be used as electrodes and electrolytes in aluminum ion capacitors. The materials must be stable, conductive, and able to withstand the high voltage and current densities that are required for high-performance energy storage. Another challenge is the development of manufacturing processes that can be used to produce aluminum ion capacitors on a large scale. Currently, Al-ion supercapacitors (AISCs) are made using batch processes, which are time-consuming and labor-intensive. To be commercially viable, aluminum ion capacitors need to be produced using continuous, scalable processes that can be automated and easily integrated into existing manufacturing processes. Despite these challenges, there has been significant progress in the development of aluminum ion capacitors in recent years. Researchers have developed new materials and processes that have improved the performance and stability of aluminum ion capacitors, and several companies are now working to commercialize this technology.

Some researchers have developed new electrolyte formulations that have improved the stability and ionic conductivity of the electrolyte, leading to increased performance and longer cycle life. Other researchers have developed new electrodes that have increased the capacitance of the device, allowing for even higher energy densities. A specific area of progress has been the development of hybrid aluminum ion capacitors, which combine the benefits of traditional capacitors and batteries. Hybrid aluminum ion capacitors use a combination of a conventional capacitor and an aluminum ion capacitor to create a device that has both high energy density and high-power output. These hybrid devices have been shown to have excellent performance and stability, making them well-suited for a wide range of applications. Advances have also been made in the integration of aluminum ion capacitors into larger energy storage systems. Particularly, researchers have developed energy storage systems that use Al-ion capacitors in combination with other energy storage technologies, such as batteries and supercapacitors, to create hybrid energy storage systems that are more efficient and cost-effective than traditional systems. These hybrid systems are being developed for a variety of applications, including backup power systems, renewable energy integration, and electric vehicles. As a matter of fact, aluminum ion capacitors are a promising technology that has the potential to revolutionize energy storage.

The mechanism of an Al-ion SC can be understood by examining the reactions that occur at the electrodes and the electrolyte. In an Al-ion capacitor, the positive electrode is made of a porous material that allows aluminum ions to be stored in the electrical field, and the negative electrode is typically made of a conductive material such as carbon. The electrolyte is a liquid or gel that contains positively charged aluminum ions and provides a pathway for ion transport between the electrodes. When a voltage is applied to the electrodes, aluminum ions are attracted to the positive electrode, where they are stored as part of the electrical field. The reaction at the positive electrode can be represented by the following equation:

Al^3+^ + e^−^ => Al(s). (4)

At the negative electrode, electrons are injected into the electrode and combined with the Al ions in the electrolyte to form aluminum metal. This reaction can be represented by the following equation:

Al(s) + e^−^ => Al^3+^ + e^−^. (5)

These reactions are reversible, and the energy stored in the capacitor can be released by reversing the voltage. This causes the aluminum ions to move back to the negative electrode and the electrons to move back to the positive electrode, allowing the device to be charged and discharged repeatedly. It is important to note that the reactions in an Al-ion capacitor are different from those in a traditional battery. In a battery, the reactions involve the transfer of ions between the electrodes, which results in the formation of metal hydroxides or other species that can degrade the electrodes and electrolytes over time. In contrast, the reactions in an Al-ion capacitor are stable and do not result in the formation of degradation products, allowing for long cycle life and high reliability.

### 6.5. Li-Ion vs. Al-Ion Supercapacitors

Li-ion SCs and Al-ion SCs are both types of hybrid energy storage devices that combine the advantages of traditional capacitors and batteries. One of the key differences between Li-ion capacitors and Al-ion capacitors is the type of ions used for energy storage. Li-ion capacitors use lithium ions as the charge carriers, while Al-ion capacitors use aluminum ions. The choice of ion has a significant impact on the performance and stability of the device. For example, Al^3+^ ions are larger and more abundant than Li^+^ ions, making them more readily available and potentially less expensive. Additionally, Al ions have a higher oxidation state than Li ions, which allows them to store more energy in the electrical field. Another difference between Li-ion capacitors and Al-ion capacitors is the composition of the electrodes and electrolytes. Li-ion capacitors typically use a porous carbon material as the positive electrode and a lithium metal oxide material as the negative electrode. In contrast, Al-ion capacitors typically use a porous Al-based material as the positive electrode and a conductive carbon material as the negative electrode. Li-ion capacitors and Al-ion capacitors can also differ in terms of their performance characteristics. For example, Li-ion capacitors tend to have higher energy densities than Al-ion capacitors, which makes them well-suited for applications that require high energy storage in a compact form factor. However, Al-ion capacitors tend to have longer cycle lives and higher power outputs than Li-ion capacitors, which makes them well-suited for applications that require high power and long-term stability.

**Figure 15:**
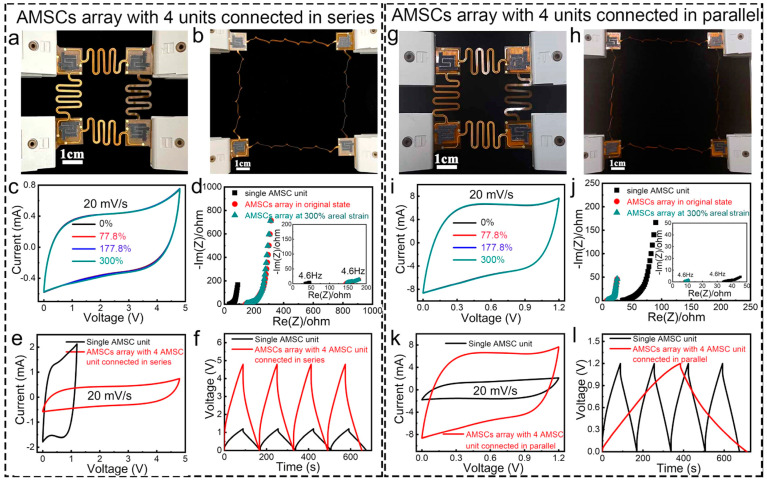
Real-time images of the fabricated stretchable AMSC array consisting of four MSC units interconnecting in (**a**) series or (**g**) parallel being stretched from the initial state to (**b**,**h**) 300% biaxial areal strain; (**c**,**i**) corresponding CV trails and (**d**,**j**) Nyquist impedance profiles; comparison of CV and GCD trails of single AMSC unit and AMSC array consisting of four MSC units interconnected in (**e**,**f**) series or (**k**,**l**) parallel. Reproduced with permission from [219]. Copyright 2022 American Chemical Society.

Presently, Li-ion capacitors and Al-ion capacitors are both promising technologies that have the potential to revolutionize energy storage. The choice of technology will depend on the specific application and the desired performance characteristics. Both technologies offer unique advantages and disadvantages, and it is likely that they will continue to evolve and improve in the coming years. In this context, it is desirable to investigate the role that MXenes can play in AISCs. Wu et al. [219] aimed to improve the performance of a symmetric Al^3+^ ionic micro-supercapacitor (AMSC) by expanding the interlayer space of the Ti_3_C_2_T*_x_* electrode using polypyrrole-coated bacterial cellulose nanospacers. The microsupercapacitor was made with Ti_3_C_2_T*_x_*/BC@PPy nanostructured thin-film electrodes and a polyacrylamide/AlCl_3_·6H_2_O hydrogel electrolyte. The use of nanospacers led to a significant increase in the area capacitance of the microcapacitor compared to a cell with a bare Ti_3_C_2_T*_x_* electrode. The authors also proposed a design for a biaxially stretchable micro-supercapacitor array with high energy efficiency, which utilizes island-bridge interconnect architecture. This design uses an editable heptapolyimide film embedded with conductive copper foil as a stretchable bridge. As shown in Figure 15, the combination of biaxial stretchability and high energy density makes the proposed microcapacitor array a promising solution for flexible energy storage.

## 7. Binary MXene-Based Supercapacitors

Porous carbon microspheres derived from pure biomass are interesting for supercapacitor applications due to low cost, high yield, and ease of preparation. Wei et al. [220] developed a composite material for supercapacitor applications made from chitosan-based porous carbon microspheres and Ti_3_C_2_T*_x_* MXene through electrostatic interaction. The resulting CPCM/MXene nanostructure showed a high specific capacitance of 362 F g^−1^ and good rate performance with capacitance retention of 94% after 10,000 cycles at 10 A g^−1^. The assembled CPCM/MXene supercapacitor delivered an ED of 28 Wh kg^−1^ at a PD of 500 W kg^−1^.

Another approach is to use high-quality carbon materials that have a well-defined porous structure and high surface area. For example, materials such as mesoporous carbon, hierarchical porous carbon, and carbon aerogels have shown promising results in hybrid capacitors due to their high surface area and well-defined porous structure. These materials can effectively improve the capacitance and stability of the hybrid capacitors. Finally, the use of advanced fabrication techniques such as template-assisted synthesis, electrospinning, and self-assembly can also help to improve the mechanical stability and electron transport efficiency of porous carbon materials in hybrid capacitors. These techniques allow for the controlled synthesis of carbon materials with well-defined porous structures and can help mitigate the self-assembly problem of MXenes. In fact, there are several approaches that can be used to improve the mechanical stability and electron transport efficiency of porous carbon materials in hybrid capacitors. The development of high-quality carbon materials, the functionalization of carbon materials with appropriate chemical groups, and the use of advanced fabrication techniques are all promising avenues for further research in this area.

Sharma et al. [221] developed a simple method to combine the benefits of incorporating black phosphorus (BP) nanoparticles into Ti_3_C_2_T*_x_* MXene sheets and the protective effect of L-ascorbic acid encapsulation of Ti_3_C_2_T*_x_*-BP nanostructures against oxidation, even under high-temperature conditions, during hydrothermal reactions. The intercalation of BP and the subsequent passivation with antioxidants helped prevent oxidation at high temperatures above 100 °C during hydrothermal processes. The authors optimized the concentration of BP to improve the electrochemical performance of Ti_3_C_2_T*_x_*-BP nanostructures. The thermal stability of the Ti_3_C_2_T*_x_*-BP hybrid was studied using temperature-dependent Raman spectroscopy. A stable behavior was demonstrated, even at high temperatures, due to the encapsulation of the Ti_3_C_2_T*_x_*-BP nanostructures with *L*-ascorbic acid. The presence of surface termination between Ti_3_C_2_T*_x_* and BP led to the formation of Ti-O-P bonds at the interface, which enhanced electron transfer by improving the Coulombic reactions between the core and valence states of the Ti-O-P interfacial bonds. The resulting symmetric Ti_3_C_2_T*_x_*-BP electrode had a capacitance of 120 mF cm^−2^ at 0.4 mA cm^−2^, with excellent retention of 95% and Coulombic efficiency of 92% after 10,000 cycles due to the unique intercalation and encapsulation strategies. The analysis of the Raman spectra demonstrated the suitable temperature of up to 150 °C for in situ growth of black phosphorous at MXenes without compromising its properties. This research provides new insights into how nanostructures can be tuned for better electrochemical performance at high temperatures while resisting oxidation. Zheng et al. [222] developed a simple and efficient method for synthesizing TMA^+^-MnO_2_ birnessite nanosheet films in a concentrated LiTFSI electrolyte for use in supercapacitors. The assembled device exhibited a high operating voltage range of 0–1.6 V and delivered a high ED of 86.5 Wh L^−1^ and PD of 268 W L^−1^ at a 2 mV s^−1^ scan rate. The high performance was attributed to the combination of Mo_1.33_CT_z_ as the positive electrode and TMA^+^-MnO_2_ as the negative electrode. This research provides a path to produce high-performance asymmetric supercapacitors with large voltage windows, high energy and power densities, and low self-discharge rates.

**Figure 16:**
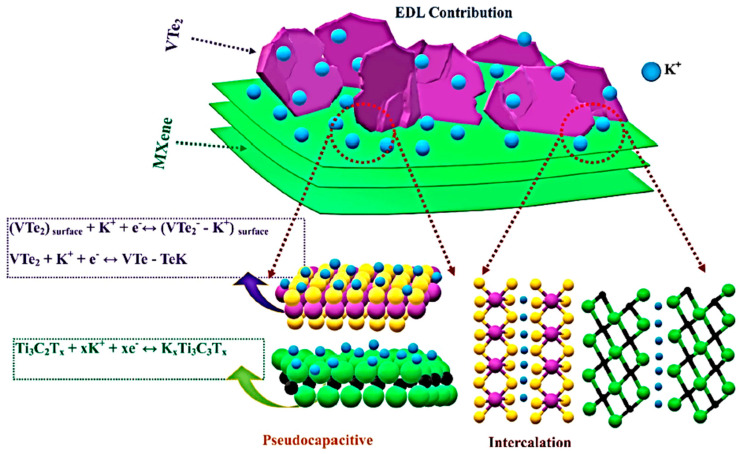
Schematic diagram representing the plausible charge storage mechanism in the VTe_2_/MXene heterostructure during the electrochemical reactions. Reproduced with permission from [223]. Copyright 2022 Royal Society of Chemistry.

Sree Raj et al. [223] developed a simple hydrothermal synthesis method to produce bare VTe_2_ and VTe_2_/MXene nanostructured electrodes for supercapacitors (see Figure 16). The synergistic interaction effect increases the nanostructure’s specific capacitance to 250 F g^−1^ with good cycling stability. The assembled asymmetric device (VTe_2_/MXene as positive electrode, MoS_2_/MXene as negative electrode) delivered an ED of 46.3 Wh kg^−1^ and a PD of 6400 W kg^−1^. DFT studies indicate that increasing Te 5p states near the Fermi level due to MXene enhances the energy storage performance of the VTe_2_/Ti_3_C_2_ nanostructures. The charge storage mechanism in these nanostructures involves a combination of electric double-layer formation, surface-bound redox reactions, and K^+^ ion intercalation into van der Waals channels.

Mahmood et al. [224] fabricated a hybrid electrode for supercapacitor applications consisting of MoO_3_ nanowires deposited on Ti_3_C_2_T*_x_*/carbon cloth (CC). This hybrid showed a higher capacitance compared to bare MoO_3_ on CC, with a capacitance of 783.4 F g^−1^ at a 5 mV s^−1^ scan rate and 775 F g^−1^ at 1 A g^−1^ current density and a capacitance retention of 96.4% after 6000 cycles. Wang et al. [225] designed and synthesized a novel nanosheet-on-nanosheet structure consisting of vertically aligned ZnCo_2_O_4_ nanosheets (ZCO) deposited on layered Ti_3_C_2_ nanosheets (d-TC) for supercapacitor applications. The robust nanosheets provide excellent structural stability, fast electron and ion transport, and a large electroactive surface area, resulting in a good electrochemical storage capacity of 196 C g^−1^. The asymmetric device composed of ZCO/d-TC//activated carbon (AC) showed an ED of 15.6 Wh kg^−1^ and a PD of 551.1 W kg^−1^ with high long-term stability of 89.5% after 4000 cycles. Arsen et al. [226] developed a ruthenium cobalt oxide (RuCo_2_O_4_)/Ti_3_C_2_T*_x_* nanostructure as a binder-free bifunctional electrode for overall water splitting and supercapacitors using electrophoretic deposition of Ti_3_C_2_T*_x_* on nickel foam followed by the growth of RuCo_2_O_4_ nanostructures. The RuCo_2_O_4_/Ti_3_C_2_T*_x_* symmetric device delivered a high capacitance of 229 F g^−1^, an ED of 20.4 Wh kg^−1^ at a PD of 2400 W kg^−1^ and a capacitance retention of over 90% after 5000 cycles. The improved performance of the RuCo_2_O_4_/Ti_3_C_2_T*_x_* supercapacitor is attributed to the interaction between RuCo_2_O_4_ and Ti_3_C_2_T*_x_* for efficient charge transfer to the porous structure of RuCo_2_O_4_/Ti_3_C_2_T*_x_* nanostructures for improved electrical active sites and ion transport, and to the synergistic effect from a large amount of charge transfer on the RuCo_2_O_4_/Ti_3_C_2_T*_x_* interface.

A nanostructure was fabricated by combining Ti_3_C_2_T*_x_* with Co_3_V_2_O_8_ to achieve higher capacity and long-term stability for supercapacitor applications [227]. The hollow Co_3_V_2_O_8_@Ti_3_C_2_T*_x_* nanospheres were made by a one-step hydrothermal method using Ti_3_C_2_T*_x_* flakes as precursors. The optimized Co_3_V_2_O_8_@ Ti_3_C_2_T*_x_* nanostructure showed high specific capacity (3 F cm^−2^ at 8 mA cm^−2^) and good cycling stability (94.5% over 10,000 cycles). The asymmetric device Co_3_V_2_O_8_@Ti_3_C_2_T*_x_*//AC achieved an ED of 70.2 μWh cm^−2^ at a PD of 3.3 kW cm^−2^ and maintained 87.1% of its performance over 10,000 cycles. Additionally, the device demonstrated high mechanical flexibility with minimal capacitance decay under different bending deformations. He et al. [228] reported a simple filtration method to create 2D/1D nanostructures composed of Ti_3_C_2_T*_x_* and MnO_2_ nanoribbon stack structures for electrochemical storage. The Ti_3_C_2_T*_x_*/MnO_2_ nanostructured electrode showed a gravimetric capacitance of 315 F g^−1^ at 10 mV s^−1^ and a good rate capability of 166 F g^−1^ at 100 mV s^−1^. This is due to the efficient charge transfer between MnO_2_ and Ti_3_C_2_T*_x_*. An asymmetric device with Ti_3_C_2_T*_x_*/MnO_2_ as the cathode and alkalized Ti_3_C_2_T*_x_* as the anode showed an ED of 16.1 Wh kg^−1^ (43.4 Wh cm^−2^) at a PD of 351 W kg^−1^ (0.95 mW cm^−2^) due to the synergistic effect that increases capacitance. Xu et al. [229] described the creation of a supercapacitor electrode material by combining NiMoS_4_ with dopamine (DA)-doped Ti_3_C_2_. The dopamine was easily adsorbed by the Ti_3_C_2_ surface to form a negatively charged layer, which was beneficial to the metal ion contact and enrichment of NiMoS_4_. The resulting Ti_3_C_2_-DA/NiMoS_4_ provided a high capacitance of 1288 F g^−1^ at a current density of 1 A g^−1^, and the assembled device delivered an ED of 40.5 Wh kg^−1^ at a PD of 810 W kg^−1^ with an efficiency of 89% over 9000 cycles. Zhu et al. [230] reported the creation of a flexible yet robust composite paper made of Ti_3_C_2_T*_x_* MXene and bamboo microfibrils. The paper was made through vacuum filtration and showed higher tensile strength (49.5 MPa) and conductivity (4.8 × 10^3^ S m^−1^) compared to bare nanostructures. The paper was also able to withstand 1000 cycles of tension, bending, and compression without degradation. The composite paper was used to create wearable electronics and showed excellent energy storage stability and linear sensing performance in the pressure range of 0–2.5 kPa. Electric heaters were also made with a minimum input voltage of 2.5 V.

Niu et al. [231] reported the use of a hydrothermal reaction to introduce metal ions (CoFe or CoMn) onto Ti_3_C_2_T*_x_* nanosheets to form 2D/2D hierarchical structures. The addition of Fe ions improved the specific capacity, while the addition of Mn ions improved the rate performance. The highest capacitance was seen in the CoFe/Ti_3_C_2_T*_x_* electrode, which showed a capacitance of 808 F g^−1^ at 0.5 A g^−1^, higher than that of CoMn/Ti_3_C_2_T*_x_* (562 F g^−1^) and Co(OH)_2_/Ti_3_C_2_T*_x_* (270 F g^−1^). This was attributed to the synergistic effect between CoFe-LDH and Ti_3_C_2_T*_x_* and the improvement in conductivity and cycle stability due to the introduction of metal ions. Wang et al. [232] demonstrated a high-performance supercapacitor electrode material by creating a 3D polyaniline (PANI) architecture on hydroxyl-terminated Ti_3_C_2_T*_x_* (H-Ti_3_C_2_T*_x_*). The hydroxyl groups were created from the stepwise removal of Al from the MAX phase, allowing for chemical exfoliation of Ti_3_C_2_T*_x_*. The negatively charged H-Ti_3_C_2_T*_x_* attracted emeraldimine-based PANI nanosheets, breaking the hydrogen bond between N-methylpyrrolidone and PANI and leading to a uniform coating of PANI on the layered structure of H-Ti_3_C_2_T*_x_*. The resulting PANI/H-Ti_3_C_2_T*_x_* nanostructure exhibited a capacitance of 464 F g^−1^ at 1 A g^−1^ with a capacitance retention of 87.5%. This surface functionalization of Ti_3_C_2_T*_x_* is thus an effective way to improve its electrochemical activity.

Khumujam et al. [233] designed and tested a fibrous asymmetric supercapacitor with a wet-spun Ti_3_C_2_T*_x_*/PAN fiber-based negative electrode and a NiCo_2_S_4_ electrodeposited positive electrode. The resulting device delivered an ED of 40.7 mWh cm^−3^ and a PD of 301.51 mW cm^−3^ using a PVA/KOH solid electrolyte. The design utilized a simple wet-spinning method to prepare the carbon fibers with a hierarchical porous structure, resulting in high porosity and a specific surface area of 550 m^2^ g^−1^. This approach solved the problem of low capacitive performance and conductivity of PAN-derived carbon fibers by incorporating highly conductive MXene nanosheets into PAN to form wet-spun MX/PAN carbon fibers. Wang et al. [234] demonstrated that the integration of polyaniline (PANI) nanoparticles with MXene in the interlayer structure of the electrode can result in high-energy-density asymmetric supercapacitors. The study found that a compact PANI/MXene thin film electrode was created by adding a small amount of 10 nm-sized PANI nanoparticles into the MXene interlayer. This resulted in an ED of 65.6 Wh L^−1^ at a PD of 1687 W L^−1^ due to the unique structure of the electrode, which showed excellent synergistic effects and electrochemical activity. The high loading of positive electrochemically active PANI nanoparticles in the interlayer of the nanostructured film also led to a high working voltage of 0.8 V. The scalable fabrication procedures and rational configuration engineering make asymmetric flexible memory devices attractive and promising.

Pan et al. [235] fabricated a flexible asymmetric supercapacitor device using a self-assembled Ti_3_C_2_T*_x_*/MoO_3_ nanostructure as the cathode and a mutually exclusive a-CNTs/K*_x_*MnO_2_ nanowire network as the anode. The Ti_3_C_2_T*_x_*/MoO_3_ nanostructured electrode showed a capacity of 371 C g^−1^ at 1 A g^−1^ and maintained 89.5% after 6000 cycles due to the synergistic effect of the highly conductive Ti_3_C_2_T*_x_* and high pseudocapacitive MoO_3_ nanoribbons. The a-CNTs/K*_x_*MnO_2_ electrode showed improved rate performance and capacitance through K^+^ ion pre-intercalation, which expanded and stabilized the diffusion channels of electrolyte cations. The assembled a-CNTs/K*_x_*MnO_2_//Ti_3_C_2_T*_x_*/MoO_3_ device achieved a high capacitance of 65.5 F/g^−1^, an ED of 36.4 Wh kg^−1^, and a PD of 863.5 W kg^−1^ at an operating voltage of 2 V, with a cycling stability of 91.7% after 6000 cycles at 5 A g^−1^ in an aqueous electrolyte. It is important to find ways to improve the mechanical stability and electron transport efficiency of porous carbon materials in hybrid capacitors. One approach is to functionalize the carbon materials with appropriate chemical groups that can improve their mechanical stability and electron transport efficiency. For example, introducing oxygen-containing functional groups such as carboxylic acids or hydroxyl groups can improve the mechanical stability of the carbon materials and increase the number of active sites for ion adsorption. In addition, the incorporation of conductive nanomaterials such as graphene or carbon nanotubes into the carbon materials can also improve the electron transport efficiency and overall performance of the hybrid capacitors.

A highly sTable 1T-MoS_2_ nanosheet was synthesized using W doping and in situ growth on a 2D Ti_3_C_2_T*_x_* template [236]. By controlling the phase transition of 1T-MoS_2_ through W-doping, the Ti_3_C_2_T*_x_* template provided a uniform space for the growth of 1T-MoS_2_, leading to a well-matched nanostructure of 1T-Mo_0.71_W_0.29_S_2_/Ti_3_C_2_T*_x_*. This structure showed a capacitance of 284 F g^−1^ at 1 A g^−1^ with a capacitance retention of 99.2% after 8000 cycles, and a flexible symmetric device delivered an ED of 9.3 Wh cm^−2^ at a PD of 7.1 kW cm^−2^ with excellent stability and flexibility. Wan et al. [237] developed a nanostructure consisting of metallic 1T-MoS_2_ nanosheets and Ti_3_C_2_T*_x_* through one-pot hydrothermal synthesis. The resulting structure showed a high capacitance of 206.3 F g^−1^ at 1 A g^−1^, with fast ion transport capability due to Ti_3_C_2_T*_x_*, leading to a capacitance of 150 F g^−1^ at 20 A g^−1^ with a 73% holding capacitance, much higher than that of 1T-MoS_2_ alone. The flexible asymmetric device made of 1T-MoS_2_/Ti_3_C_2_T*_x_* as the negative electrode and δ-MnO_2_ as the positive electrode exhibited a wide voltage window of 1.8 V and high areal ED of 69 Wh cm^−2^ at a PD of 4500 W cm^−2^, demonstrating its good rate capabilities.

### 7.1. MXene/Noble Metal Nanostructures

Incorporating or coupling noble metals with MXene-based electrodes can significantly increase their electrical conductivity, which is a crucial factor for improving their rate capacitance and stability. Other methods for enhancing the conductivity of MXene-based electrodes include introducing metal nanoparticles or noble metals, N-doping, and coupling or intercalation with conducting polymers. These strategies can result in improved electrochemical performance and overall energy storage capacity of MXene-based supercapacitors. Zheng et al. [238] developed a simple two-step in situ synthesis of free-standing Ag nanostructures deposited on 3D Ti_3_C_2_T*_x_* films. During this process, the Ti_3_C_2_T*_x_* served as a reducing agent to anchor the hybrid structure, while Ag nanoparticles were directly formed from AgNO_3_ reduction through −OH termination, improving the ion transfer rate of the 3D Ti_3_C_2_T*_x_*/Ag nanostructure. The synthesis process utilized ice particles as self-sacrificing templates to build the 3D framework. The 3D Ti_3_C_2_T*_x_*/Ag hybrid electrode produced a capacitance of 356 F g^−1^ at 2 mV s^−1^ and maintained a capacitance retention of 94.7% after 40,000 cycles at 10 A g^−1^. Another study by Li et al. [239] showed that the introduction of Ag nanoparticles on the surface of Ti_3_C_2_T*_x_* MXene increased the available active sites for electrochemical reactions and improved the conductivity, leading to higher energy storage capacity and longer cycle stability in supercapacitors. The results indicated that the Ag-modified Ti_3_C_2_T*_x_* electrodes had a high specific surface area of 107 m^2^ g^−1^, high areal capacitance of 332 mF cm^−2^ at 2 mV s^−1^ and long-term cycling stability of 87% after 10,000 cycles, demonstrating its potential for practical applications in supercapacitor devices. Zheng et al. [240] demonstrated that the capacitance of MXene can be improved by coupling with Au nanoparticles. In their study, a specific capacitance of 278 F g^−1^ was achieved at a scan rate of 5 mV s^−1^, with 95% capacitance retention observed after 10,000 cycles of charge-discharge cycling. However, the use of Au nanoparticles in this study is limited by their higher cost compared to Ag nanoparticles, difficulties in scalability, and instability in the physical mixing of Au nanoparticles with MXene. As a result, developing a flexible and self-contained 3D MXene-based hybrid with improved capacitance and stability remains a significant challenge in the field.

Tang et al. [241] developed a nanostructured paper that is highly conductive and has a high specific surface area. The paper was created using a vacuum-assisted filtration strategy with functional additives. The cellulose filaments, which were made by mechanical grinding, have a large aspect ratio and abundant hydroxyl groups, which contribute to stronger hydrogen bonding and better entanglement. This unique structural feature makes the nanostructured paper strong and allows for better accessibility of electrolytes and storage of charges generated in the system. The addition of silver nanowires interspersed between the cellulose filaments further improves the mechanical strength and electrical conductivity of the paper, leading to an excellent electrochemical storage activity and high capacitance of 505 F g^−1^ at 10 mV s^−1^ even with just 25 wt.% Ti_3_C_2_T*_x_*. This highly conductive paper electrode has great potential for large-scale fabrication using mature papermaking techniques. Munir et al. [242] reported the enhancement of the electrochemical properties of vanadium pentoxide through the synergistic effects of noble metal doping and composite formation. The authors achieved this improvement using a simple sonication method to intercalate V_2_O_5_ nanowires and Ag-doped V_2_O_5_ onto the Ti_3_C_2_T*_x_* surface. This strategy resulted in improved conductivity and electrochemical behavior, as evidenced by an 875 F g^−1^ increase in specific capacitance of the Ag-doped V_2_O_5_/Ti_3_C_2_T*_x_*@ITO electrode (at a current density of 1 A g^−1^), with 93.9% capacitance retention after 3000 cycles. The enhanced electrochemical capacitance was attributed to the highly active sites created by the Ag doping and the ternary nanostructured V_2_O_5_ nanowires, which reduced the restacking of the Ti_3_C_2_T*_x_* layer. Patil et al. [243] developed an asymmetric flexible supercapacitor with a sTable 2D/2D core-shell structure (Co_3_(PO_4_)_2_@Co_2_Mo_3_O_8_) as the positive electrode and 2D CNT–Ti_3_C_2_T*_x_* as the negative electrode. The positive electrode had a capacity of 185 mAh g^−1^ and 95.6% capacity retention, while the negative electrode had an areal capacitance of 187.5 mF cm^−2^ and 93.1% cycle stability. The assembled device had a volumetric capacitance of 7.9 F cm^−3^, an ED of 74.06 Wh kg^−1^, and a PD of 1130 W kg^−1^, with an efficiency of 93.2% after 5000 cycles. The unique structural properties were attributed to these performance characteristics. This flexible device can operate at up to 4.5 V and has excellent flexibility, making it suitable for wearable and portable memory device technologies.

### 7.2. MXene/Nanofibers

Fiber-MXene-based capacitors are a relatively recent innovation in the energy storage field. They are created by combining MXene nanosheets with a fiber substrate, which offers a unique blend of high surface area and robustness. This combination is highly desirable for use in wearable technology, flexible electronics, and energy harvesting systems, where a flexible and lightweight power source is required [244]. It creates hybrid structures that exhibit improved conductivity and show reduced restacking. For example, Xue et al. [245] reported a Ti_3_C_2_T*_x_*/aramid nanofiber nanostructure film that was prepared using a simple freezing method. The resulting structure showed a capacitance of 174.3 F g^−1^ at 0.05 A g^−1^ and a capacitance retention of 84.4% after 10,000 cycles. This improved performance was attributed to the smaller size of the 2D nanosheets, which effectively shortened the ion diffusion path and increased the storage capacity of electrolyte ions in the gaps between the nanosheets. Additionally, the integration of aramid nanofibers, which were obtained from Kevlar yarns, enhanced the mechanical strength of the composite structure.

Fiber-MXene capacitors can be manufactured through various methods such as electrospinning, drawing, and coating. Electrospinning uses an electric field to spin a polymer solution into fibers, while drawing involves the mechanical stretching of fibers. Both techniques result in fibers with a high surface area and strength. Coating, on the other hand, involves depositing MXene nanosheets onto a fiber substrate using techniques like chemical vapor deposition (CVD). Fiber-MXene capacitors have several advantages over traditional supercapacitors. First, their high surface area enhances ion adsorption, leading to high capacitance values. Second, the fiber substrate’s robustness enables the creation of flexible and bendable devices, which is crucial for wearable technology and flexible electronics. Lastly, the high surface area and robustness also make fiber-MXene capacitors suitable for integration into energy harvesting systems where energy is collected from sources such as light, heat, and motion.

The combination of MXene nanosheets and polymer fibers results in a highly flexible and scalable supercapacitor with improved performance. The high surface area of the MXene nanosheets enhances ion adsorption and provides high electrostatic capacity, while the polymer fibers maintain a constant capacitance under extreme conditions like high temperatures and mechanical strain. Additionally, the flexibility and scalability of the polymer fibers make fiber-MXene capacitors suitable for integration into various devices and systems, including wearable electronics and flexible energy storage devices. Studies have confirmed the feasibility of fiber-MXene capacitors. For instance, researchers at the National University of Singapore demonstrated that fiber-MXene capacitors can be easily integrated into wearable electronics and exhibit excellent mechanical stability and capacitance retention under high temperatures and strain [246]. Researchers at Zhejiang Sci-Tech University showed that fiber-MXene capacitors have high energy density and good rate performance, making them suitable for high-power applications [247]. Due to the improved porous generation, ordered porous pathways, large exposed surface, and in situ interfacial electron transfer, the ZIF-67@Ti_3_C_2_T*_x_* fiber displays excellent volumetric capacitance (972 F cm^−3^) and long-term cycling stability (90.8% capacitive retention after 20,000 cycles) in 1 mol L^−1^ KOH electrolytes.

The progress in fiber-MXene capacitors has been impressive, and their use in energy storage applications shows great potential. With ongoing research and development, it is expected that fiber-MXene capacitors will play an increasingly important role in the energy storage landscape in the future. They offer several advantages over traditional supercapacitors, including high capacitance, scalability, and flexibility, which makes them suitable for various energy storage applications, such as wearable electronics, flexible energy storage devices, and high-power applications. Guo et al. [248] developed Ti_3_C_2_T*_x_*-based wet-spun fibers using a two-step strategy. The gaps between the MXene laminate structure and the penetrating mesoporous Ti_3_C_2_T*_x_* nanosheets provide better electron and ion transport, leading to enhanced energy storage. The prepared porous fiber electrode showed higher volumetric capacitance compared to the non-porous fiber electrode. The symmetric fiber-based device showed high capacitance, energy density, and power density, making it suitable for use in flexible and wearable energy storage devices. Kumari et al. [249] reported on the modification of the synthesis procedure for Ti_3_C_2_T*_x_*-A2 MXene to produce a supercapacitor with high capacitance and energy density. The modification involved the use of the LiF-HCl etching method to preserve Al traces within the Ti_3_C_2_T*_x_* layer, which acts as an extra electron carrier between the nanosheets. The resulting Ti_3_C_2_T*_x_*-Al electrode showed a gravimetric capacitance of 513 F g^−1^ at 2 A g^−1^ with remarkable stability over 10,000 cycles. The flexible symmetric device made of this electrode material delivered an ED of 49.2 mWh cm^−2^, PD of 969 mW cm^−2^, and a capacitance retention of 83.3% over 10,000 cycles.

### 7.3. MXene/Carbon Supercapacitors

Yang et al. [250] developed a method for depositing Ti_3_C_2_T*_x_* nanosheets onto porous carbon nanotube sponges to form Ti_3_C_2_T*_x_*@CNT nanostructures. The CNT sponge helps to anchor the Ti_3_C_2_T*_x_* nanoflakes, suppressing restacking and forming a continuous 3D scaffold for fast charge transport. The resulting Ti_3_C_2_T*_x_*@CNT nanostructured sponge showed a high gravimetric capacitance of 468 F g^−1^ and a good retention rate of 80% at 100 mV s^−1^, and the assembled symmetric device showed an ED of 12.23 Wh cm^−2^ with 93% retention after 10,000 cycles. By controlling the deposition potential, the authors were able to partially oxidize the dispersed MXene nanoflakes in situ and enhance surface-controlled pseudocapacitance.

Xu et al. [251] reported the preparation of a flexible electrode using a Ti_3_C_2_T*_x_*/CMC-PANI nanostructure. This was achieved through in situ polymerization of aniline on the surface of carboxymethylcellulose (CMC), which extended the interlayer spacing of the Ti_3_C_2_T*_x_* nanosheets. The resulting nanostructured electrode had a capacitance of 1161.4 mF cm^−2^ and a remarkable mechanical strength of 35.6 MPa, making it suitable for use in flexible devices. The introduction of CMC-PANI improved the interfacial interaction between Ti_3_C_2_T*_x_* and the electrolyte ions and promoted micro-meso-porosity through its interweaving structure, thus contributing to fast ion diffusion and improved charge storage. The study shows that this design balances flexibility and capacity and has the potential to provide a solution to the drawbacks of MXenes and pave the way for the development of more efficient energy storage devices.

Bai et al. [252] reported that a vacuum filtration strategy was used to create self-supporting carbon@Ti_3_C_2_T*_x_* electrodes with 3D mesoporous structures using polystyrene spheres of different sizes and ratios as templates. This mesoporous structure in the carbon@Ti_3_C_2_T*_x_* electrode was found to improve the energy storage capacity and sensing activity of the Ti_3_C_2_T*_x_* materials by effectively alleviating the stacking of Ti_3_C_2_T*_x_* nanosheets. The electrode had a high capacitance of 448 F g^−1^ at 1 A g^−1^, and the assembled symmetric device had a high ED of 304 Wh g^−1^ at a PD of 11.92 W g^−1^ due to its unique electrode structure features.

The main challenge in utilizing the benefits of Ti_3_C_2_T*_x_* for electrochemical applications is its tendency to self-accumulate and restack during preparation and use, which can result in reduced electrochemical performance due to decreased active sites and hindered electrolyte ion migration [253]. Various strategies have been proposed to overcome these issues, such as creating 3D structures by incorporating other metals or metal oxides or filling columnar materials or sacrificial templates into the MXene matrix. These approaches aim to enhance the stability and electrochemical performance of Ti_3_C_2_T*_x_* [254]. The incorporation of reduced graphene (rGO) as a cross-linking agent into the gelation process of 2D MXene nanosheets can lead to the formation of hydrogels and aerogels with 3D porous structures, which can expose more active sites and enhance the electrochemical activity of Na-ion capacitors [255]. The close interfacial interactions between the crosslinked Ti_3_C_2_T*_x_* and rGO nanosheets result in a well-assembled reinforced framework network, while the point-to-surface interactions with limited active sites on the MXene surface can limit its electrochemical performance [256]. An overview of the potential of rGO and 3D Ti_3_C_2_T*_x_*/rGO frameworks as materials for designing hybrid supercapacitors. It explains that the 3D Ti_3_C_2_T*_x_*/rGO frameworks have better electrochemical performance and good wettability compared to the 3D MXene frameworks. The use of reduced graphene oxide (rGO) in hybrid supercapacitors has promising properties such as good thermal and electrical conductivity, high mechanical strength, and durability. Recent studies have shown that 3D Ti_3_C_2_T*_x_*/rGO frameworks exhibit better electrochemical performance than 3D MXene frameworks. However, the electronic conductivity and active sites on nanosheets are difficult to improve. A multi-step synthesis method using urea has been developed to create 3D nitrogen-doped Ti_3_C_2_T*_x_*/rGO frameworks with improved conductivity due to nitrogen doping [257]. This improved the specific capacitance of the supercapacitor to 463 F g^−1^ at 5 mV s^−1^ in 1 mol L^−1^ H_2_SO_4_ electrolyte and capacitance retention of 91% after 10,000 cycles, which is attributed to the macroscopic to microporous nature of the 3D framework. Overall, this is a comprehensive and concise summary of the current state of research in this area and the potential of the 3D Ti_3_C_2_T*_x_*/rGO framework as a material for designing hybrid supercapacitors. Liu et al. [258] synthesized a 3D hybrid porous aerogel made of sulfur and nitrogen doped-rGO integrated with Ti_3_C_2_T*_x_* (i.e., S-N-rGO@Ti_3_C_2_T*_x_*) nanostructure. The formation of the 3D mutual cross-linking rGO/Ti_3_C_2_T*_x_* structure was achieved through a self-assembled hydrothermal reaction; the involvement of elemental sulfur and nitrogen facilitated fast electrochemical ion transport. The assembled symmetric all-solid-state electrode of the S-N-rGO@Ti_3_C_2_T*_x_* nanostructured device showed a high capacitance of 85.4 F g^−1^ at 1 A g^−1^ with a capacitance retention of 99.8% over 20,000 cycles. The all-solid-state supercapacitor had a high ED of 24.2 Wh kg^−1^ at a high PD of 1.4 kW kg^−1^, outperforming most MXene-based supercapacitor devices. The authors demonstrated the device’s performance by lighting up LED lamps, highlighting that the self-assembled 3D aerogel is a valuable candidate for the development of efficient symmetric storage devices.

### 7.4. MXene on Carbon Cloth

**Figure 17:**
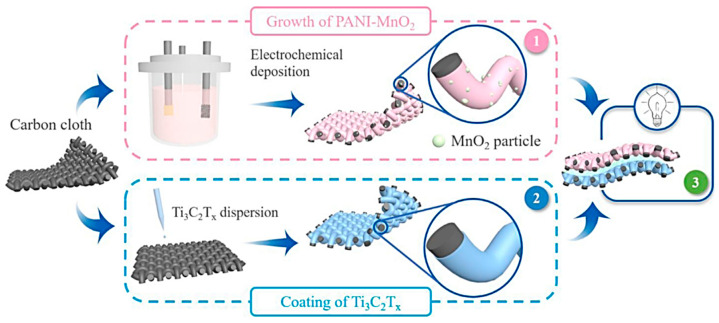
Diagram of the preparation of the asymmetric supercapacitor. Panel 1 and Panel 2 show the loading of PANI-MnO_2_ onto CC by electrochemical method and the coating of MXene onto CC using drop casting, respectively. Panel 3 of the PANI-MnO_2_//MXene asymmetric supercapacitor device. Reproduced with permission from [259]. Copyright 2022 Elsevier.

MnO_2_ and PANI are promising electrode materials due to their high specific capacitance, but the low PD of MnO_2_ and the poor cycle stability of PANI limit their development. In a study by Wei et al. [259], MnO_2_ and PANI were deposited on carbon cloth (CC) through electrochemical polymerization with LiClO_4_ to form the electrode material CC/MnO_2_-PANI. Figure 17 illustrates the assembly process of the two-electrode and asymmetric supercapacitor device. The positive electrode is composed of MnO_2_-PANI electrochemically loaded onto activated carbon cloth (CC), as shown in panel 1. The 3D texture of CC allows for the adhesion of electroactive materials without the use of adhesives, increasing mass loading in a compact area, as depicted in panel 2. In panel 3, CC/MnO_2_-PANI and CC/MXene are stacked in a PVA/H_2_SO_4_ gel electrolyte to form a flexible asymmetric supercapacitor device. The CC/MnO_2_-PANI exhibited a high specific capacitance of 634 F g^−1^ at 1 A g^−1^, which is attributed to the synergistic effect of MnO_2_ and PANI. The asymmetric device had a capacitance of 21.1 F g^−1^ at 0.5 A g^−1^, maintained 83% after 4000 cycles, and showed good durability under bending and stretching.

**Figure 18:**
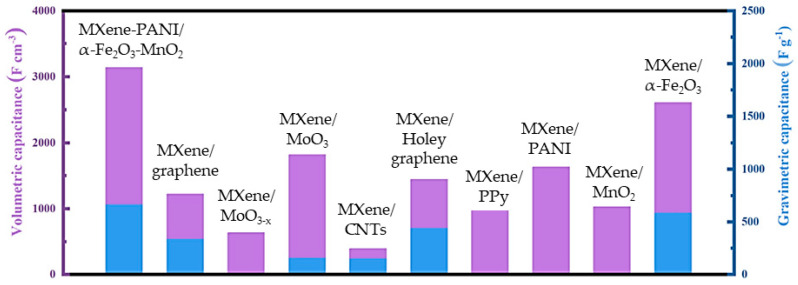
The volume capacitance and weight capacitance of MXene-PANI/α-Fe_2_O_3_-MnO_2_/MXene-PANI (MP/FM/MP-20%) electrodes are compared with other MXene-based electrodes. Reproduced with permission from [262]. Copyright 2022 Elsevier.

Ti_3_C_2_T*_x_*/PANI-based electrodes exhibit low electrochemical activity due to their internal sluggish ionic kinetics, which becomes a fundamental limitation for electrochemical activity even after increasing the Ti_3_C_2_T*_x_*/PANI loading [260]. Note, however, the good results obtained with PANI in the form of nanofibers [139]. Otherwise, to compensate for this shortcoming, Ti_3_C_2_T*_x_*/PANI thin films are usually scaled down to a few micrometers, that is, 2 mg cm^−2^ [261]. Li et al. [262] reported that the introduction of α-Fe_2_O_3_/MnO_2_ into Ti_3_C_2_T*_x_*/PANI on carbon cloth enhanced the electrochemical behavior by forming a sandwich structure. The interaction between α-Fe_2_O_3_/MnO_2_ and Ti_3_C_2_T*_x_*/PANI was found to be tight due to the large number of active sites on the surface of MXene and good hydrophilic behavior. The study also showed that the capacitance was increased to 661 F g^−1^ at a loading of 5 mg cm^−2^ with remarkable mechanical stability and flexibility. The symmetrical arrangement of this electrode material provided an ED of 53.32 Wh L^−1^ (17.45 Wh kg^−1^). The MXene-PANI/α-Fe_2_O_3_-MnO_2_ hybrid electrode compares well with other MXene-based electrodes such as Ti_3_C_2_T*_x_*/carbons [116,263,264,265], Ti_3_C_2_T*_x_*@MnO_2_ [266,267], Ti_3_C_2_T*_x_*/α-Fe_2_O_3_ [268], Ti_3_C_2_T*_x_*/PANI [261,269], or Ti_3_C_2_T*_x_*/PPY [270] (see Figure 18). Table 2 summarizes the capacitive capabilities of various MXene-based electrode materials for supercapacitors. It includes results that had not been mentioned before in this review.

**Table 2:** Capacitive capabilities of various MXene-based electrode materials for supercapacitors.

MXene Material	Electrolyte	Capacitance	Cycle Stability	Ref.
Ti_3_C_2_T*_x_*	1 M H_2_SO_4_	391 F g^−1^@2 mV s^−1^	96.3.3%@10,000	[55]
Ti_3_C_2_T*_x_*	1 M H_2_SO_4_	480 F g^−1^@2 mV s^−1^	97.1@10,000	[45]
Ti_2_C_2_T*_x_*/WS_2_/GO	1 M KOH	1111 F g^−1^@2 A g^−1^	93.1%@15,000	[152]
Ti_3_C_2_T*_x_*/Arg-CQD-Ser	1 M KOH	525 F g^−1^@1 A g^−1^	98.9%@10,000	[49]
Ti_2_CT*_x_*@C	2 M ZnSO_4_	380 mF cm^−2^	90%@10,000	[50]
Ti_3_C_2_T*_x_*/CNT	PVA/KOH gel	0.36 F cm^−2^@0.5 mA cm^−2^	90%@15,000	[263]
Ti_3_C_2_T*_x_*/rGO	1 M H_2_SO_4_	35 mF cm^−2^@1 A g^−1^	85%@10,000	[264]
Ti_3_C_2_T*_x_*/rGO	LiCF_3_SO_3_/PMMA	150 F g^−1^@50 mV s^−1^	75%@5000	[265]
Ti_3_C_2_T*_x_*/rGO foam	3 M H_2_SO_4_	463 F g^−1^@5 mV s^−1^	90.8%@10,000	[257]
Ti_3_C_2_T*_x_*/N-doped CC	-	2 F cm^−2^@1 mA cm^−2^	91%@10,000	[116]
Ti_3_C_2_T*_x_*@MnO_2_	1 M Na_2_SO_4_	390 F g^−1^@10 mV s^−1^	96%@6000	[266]
Ti_3_C_2_T*_x_*@MnO_2_	PVA/H_2_SO_4_	20.5 F g^−1^@1.5 A g^−1^	80%@3000	[267]
Ti_3_C_2_T*_x_*/NiCo_2_S_4_	KOH gel	596 C g^−1^@1A g^−1^	80%@3000	[150]
Ti_3_C_2_T*_x_*@CoNi_2_S_4_	6 M KOH	2398 F g^−1^@1 A g^−1^	80%@40,000	[65]
Ti_3_C_2_T*_x_*@Co_3_O_4_	6 M KOH	1081 F g^−1^@0.5 A g^−1^	83%@8000	[69]
Ti_3_C_2_T*_x_*@VS_2_	PVA/KOH gel	1791 F g^−1^@1 A g^−1^	90.6%@10,000	[72]
Ti_3_C_2_T*_x_*/α-Fe_2_O_3_	1M LiCl	197 F g^−1^@20 A g^−1^	97.7%@2000	[268]
Ti_3_C_2_T*_x_*/PANI	1 M Na_2_SO_4_	164 F g^−1^@2 mV s^−1^	96%@3000	[261]
Ti_3_C_2_T*_x_*/CNF/PANI	1 M H_2_SO_4_/PVA	2.9 F cm^−2^@1 mA cm^−2^	81.5@4000	[269]
Ti_3_C_2_T*_x_*/PPY	0.5 M Na_2_SO_4_	3.22 F cm^−2^@2 mV s^−1^	90%@1000	[270]
Ti_3_C_2_T*_x_*@SnS_2_/SnO_2_	1 M LiPF_6_ EC/DEC	619 mAh g^−1^@0.5 A g^−1^	93.6@2000	[185]
Nb_4_C_3_T*_x_* nanosheets	1 M H_2_SO_4_	1075 F cm^−3^@5 mV s^−1^	-	[52]
V_2_CT*_x_*@C	1 M H_2_SO_4_	551 F g^−1^ at 2 A g^−1^	88.1%@5000	[54]
Nb_2_CT*_x_*/CNT	1 M H_2_SO_4_	200 F g^−1^@1 A g^−1^	80.3%@5000	[60]
Nb_2_C/Ti_3_C_2_	PVA/H_2_SO_4_ gel	584 F g^−1^@2 A g^−1^	98%@50,000	[61]
Nb_4_C_3_T*_x_* film	1 M Li_2_SO_4_	60 F g^−1^@2 A g^−1^	90%@10,000	[63]
Mo_2_CT*_x_*	1 M H_2_SO_4_	79.1 F g^−1^@0.3 A g^−1^	89%@5000	[75]
TiVCT*_x_*/poly-oPD	1 M H_2_SO_4_	463 F g^−1^@5 mV s^−1^	89%@2000	[73]
TiVCT*_x_*@PANI-MnO_2_	1 M H_2_SO_4_	619 mAh g^−1^@0.5 A g^−1^	83%@4000	[259]

### 7.5. MOFs/MXene Supercapacitors

Among pseudocapacitive materials, MOFs, the 2D-MOFs, due to their high electronic conductivity, sufficient redox-active sites, and large ion-accessible surfaces, are considered attractive electrode materials for energy storage applications, particularly in pseudocapacitive systems. To improve MXene-based electrode performance, a facile approach is to combine Ti_3_C_2_ with metal-organic frameworks (MOFs) to form Ti_3_C_2_@MOF nanocomposites. MOFs are porous materials composed of metal ions and organic linkers. By incorporating MOFs into Ti_3_C_2_, the resulting nanocomposite can exhibit improved capacitance, stability, and rate performance. For example, by incorporating Ni-MOFs into Ti_3_C_2_, researchers have reported increased capacitance and cycling stability compared to Ti_3_C_2_ alone. This improved performance is attributed to the high conductivity and large surface area of the MOFs, which enhance the electron and ion transport in the electrode. The 2D structure of these MOFs can enhance their capacitance properties in several ways. Firstly, the in-plane charge dislocations and extended π conjugation in the MOFs increase their electronic conductivity. Secondly, the large aspect ratio of these MOFs allows for sufficient redox-active sites, which improves their pseudocapacitance. Lastly, the two-dimensional structure of these MOFs allows for large ion-accessible surfaces, reducing the diffusion distance for ion transport and improving capacitance. The authors emphasized that the use of 2D-MOFs as electrode materials in energy storage devices is a promising approach, given their attractive properties and benefits.

**Figure 19:**
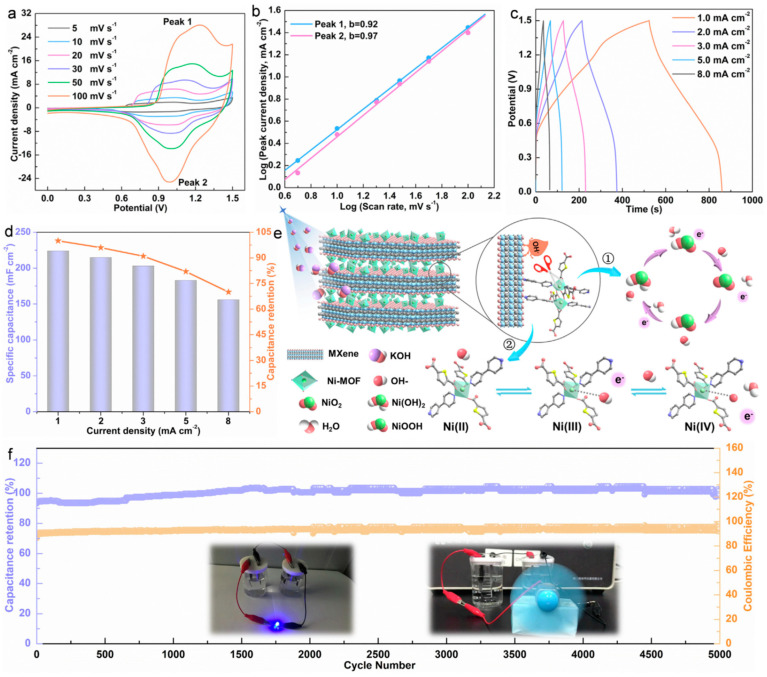
(**a**) CV curves, (**b**) Log(i) versus log(v) plots, (**c**) GCD curves, (**d**) specific capacitance and capacitance retention. (**e**) Mechanism of charge/discharge of MXene@Ni-MOF electrode: the mechanism of charge/discharge of Ni(OH)_2_ is shown in process ①, while the mechanism of charge/discharge of Ni-Bpy linear chain is shown in process ②. (**f**) Cycling ability and Coulombic efficiency at 3 mA cm^−2^ for 5000 cycles of the MXene@Ni-MOF//AC device (Inset of (**f**)), an optical image of the two MXene@Ni-MOF//AC device linked in series to light up a blue LED and power a rotating motor. Reproduced with permission from [272]. Copyright 2022 Elsevier.

Zhang et al. [271] used MXenes with oxygen-containing groups on the surface as structure-directing agents to form Ni-MOF microstrips that exhibited high performance as supercapacitor electrodes. The 2D structure of the Ni-MOFs allowed for improved conductivity and pseudocapacitance behavior. The resulting 2D Ni-MOF microstrips had abundant active sites for Faradaic redox reactions and shortened ion transport paths, leading to a good capacitance of 1124 F g^−1^ at 1 A g^−1^, which remained at 62% even at 20 A g^−1^. Zheng et al. [272] reported on the use of Ti_3_C_2_@pillared-layer Ni-MOFs, made from [Ni(thiophene-2,5-dicarboxylate)(4,4′-bipyridine)]_n_ MOF, for energy storage (Figure 19). The study showed that the Ni-MOF nanosheets anchored on MXene showed high conductivity and cycling stability due to the strong interaction between the ligands of Ni-MOF and the surface functional groups of MXene, which resulted in fast charge transfer and fast ion transport. The resulting MXene@Ni-MOF had a high capacitance of 979 F g^−1^ at 0.5 A g^−1^, with a capacitance retention of 98% after 5000 cycles.

As an anode material for supercapacitors, Ti_3_C_2_ has a low theoretical capacity. Therefore, researchers prepared nanocomposite electrode materials and conducted in-depth studies. Wu et al. [273] reported a novel strategy that combines layered Ti_3_C_2_ nanosheets with double Co/Zn MOFs polyhedral derivatives. The combination leads to improved Li-storage capacity and improved performance of the Li-ion capacitors. The nanostructure showed a capacity of 585.7 mAh g^−1^ at 0.1 A g^−1^ and a capacity retention of 93% over 1000 cycles at 2 A g^−1^ in a Li-ion half-cell. When used with activated carbon as the cathode in Li-ion capacitors, the structure showed an ED of 87.5 Wh kg^−1^ and a PD of 3500 W kg^−1^ and retained 75% after more than 6000 cycles at 2 A g^−1^. Yu et al. [274] reported a novel electrode material, V_2_CT*_x_*@NiCoMn-OH, for use in high-performance asymmetric supercapacitors. The material was created through a two-step synthesis method involving the in situ growth of NiCoMn-OH on 2D V_2_CT*_x_* MXene. The resulting 3D hollow structure provided abundant active sites for Faradaic redox reactions and improved the capacitance, with a capacitance of 827.5 C g^−1^ at 1 A g^−1^ and a capacity retention of 88.44% after 10,000 cycles. The V_2_CT*_x_*/NiCoMn-OH//AC device demonstrated an ED of 88.35 Wh kg^−1^ at a PD of 7500 W kg^−1^. Yang et al. [275] reported a nanostructured electrode combining MOF-derived CoSe_2_/Ni_3_Se_4_ nanosheets with Ti_3_C_2_T*_x_* nanosheets to form a honeycomb-like nanostructure. This structure facilitated a fast charge transfer rate and enhanced durability, leading to a high capacity of 283 mAh g^−1^ with an 80% capacity retention after 5000 cycles. The asymmetric device composed of this nanostructure showed an ED of 41.2 Wh kg^−1^ and a PD of 3.1 kW kg^−1^. This work provides new ideas for developing hierarchical nanostructures with efficient energy storage performance. Wu et al. [276] developed a one-dimensional nanostructure through the self-assembly of MXene and CoNi-bimetal MOF without the use of any templates or rigid framework. By controlling the loading of MOFs on the one-dimensional MXene fibers, the authors achieved an interconnected network of MXene fibers, CoNi, and carbon nanotubes that showed excellent microwave absorption properties. The authors suggest that this material could be used as an electrode in energy-related applications. Jia et al. [277] developed an interfacial pillar of Fe-MOF on the surface of Ti_3_C_2_T*_x_*, which created abundant electrochemically active sites. By chemically bonding MOF nanospheres with Ti_3_C_2_T*_x_* nanosheets into 3D multilayer porous nanostructures, the MOF nanospheres prevented the aggregation of Ti_3_C_2_T*_x_* nanosheets, allowing fast ion transport and maintaining more redox reaction sites. This resulted in an ED of 85.53 Wh kg^−1^ for the Fe-MOF/Ti_3_C_2_T*_x_* nanostructured electrode. This novel design offers promising potential for high-efficiency supercapacitor applications.

Wang et al. [278] developed a novel method to synthesize a hexagonal nickel-cobalt oxide nanosheet (NiCo-MOF) on a nickel foam (NF) substrate using a hydrothermal process and template-assisted electrodeposition. MXene was used as an interlayer to absorb Ni^2+^ and Co^2+^ ions. The resulting MXene-Ni-Co@NiCoMOF/NF electrode showed high specific capacity (855 C g^−1^) and excellent ED (32.6 Wh kg^−1^) when used in a supercapacitor device. This nanostructure improved the transport rate of electrons and ions and exposed numerous active sites, leading to improved performance and cycle life of the electrode. Liu et al. [279] obtained cubic ZIF-67 supported Ti_3_C_2_T*_x_* through in situ gradient etching. The addition of interlayer hierarchical cobalt hydroxide nanostructures improved ion transport and prevented the self-aggregation of Ti_3_C_2_T*_x_*. The resulting Ti_3_C_2_T*_x_*@HO-CH nanostructure showed good electrochemical capacitance, with a value of 348.6 F g^−1^ at a current density of 1 A g^−1^ and 287.3 F g^−1^ at a current density of 10 A g^−1^. Wu et al. [280] developed a hierarchically ordered ZIF-L(Zn)@Ti_3_C_2_T*_x_* nanostructure for high-performance asymmetric supercapacitors. The nanostructure was composed of nanowall array sheaths grown on anisotropic Ti_3_C_2_T*_x_* cores through chemical bonds. The ZIF-L(Zn)@Ti_3_C_2_T*_x_* nanostructure had well-developed micro-mesoporosity, ordered ion channels, fast electron conduction, and efficient intercalation that improves charge transport. The nanostructure-assembled asymmetric device showed a high capacitance of 854 F cm^−3^, an ED of 19 mWh cm^−3^, and a PD of 309 mW cm^−3^, with a stability of 90.2% over 20,000 cycles. The authors also demonstrated the use of the device for powering LED bulbs and electric fans and for self-powering water level/earthquake warning devices using ambient sunlight. Liu et al. [281] reported the preparation of hollow Ti_3_C_2_T*_x_*/ZIF-67 nanostructures for high-performance supercapacitors. The metal ions cross-linked with the surface termination groups of Ti_3_C_2_T*_x_* nanosheets and formed the nucleation site for the in-situ growth of metal-organic framework (MOF) particles on the Ti_3_C_2_T*_x_*. The 3D MOFs improved the transport of the electrolyte and avoided the accumulation of the Ti_3_C_2_T*_x_*. The resulting Ti_3_C_2_T*_x_*/ZIF-67/CoV_2_O_6_ nanostructure showed a specific capacitance of 285.5 F g^−1^. This study provides a new pathway for the design and synthesis of MXene/MOF nanostructures with customizable structures and compositions for applications. Guo et al. [282] reported a study where Ni-doped ZIF-67 was deposited using Ti_3_C_2_T*_x_* nanosheets as a template. The Ni-doped ZIF-67 was anchored onto the negatively charged Ti_3_C_2_T*_x_* surface through chemical bonds to create more active sites for enhanced electrochemical performance. The size of the NiCo-ZDH (obtained from ZIF-67 using base treatment) had a smaller surface area than the Ti_3_C_2_T*_x_*/NiCo-ZIF-67. The Ti_3_C_2_T*_x_*/NiCo-ZDH nanostructured electrode showed the highest capacitance of 877 F g^−1^ and a remarkable retention capacity of 91% over 3000 cycles. The assembled Ti_3_C_2_T*_x_*/NiCo-ZDH/AC device had an ED of 34 Wh kg^−1^ and a PD of 748 W kg^−1^. The Ni-doped ZIF-67 was self-assembled through a thermal curing process and improved the ion transport efficiency of the electrolyte during the electrochemical reaction.

Yadav et al. [54] reported the synthesis of vanadium carbide (V_2_CT*_x_*) MXene-derived metal-organic frameworks (MOFs) using a terephthalic acid linker. The MOFs were then selectively pyrolyzed into nanoporous carbons in an intercalation environment, which resulted in the formation of nanoporous carbon, improved the electronic conductivity, and showed a high capacitance of 551 F g^−1^ at 2 A g^−1^. The assembled V_2_CT*_x_*//V_2_CT*_x_* cells showed high ED (48.6 W kg^−1^ at 552 W kg^−1^) and good cycling stability, retaining 91.3% of its capacity after 5000 cycles at 10 A g^−1^. Bin et al. [283] created a flexible thin-film supercapacitor electrode using V_4_C_3_T*_x_* MXene by embedding tetra-n-butylammonium hydroxide and filtering the layered suspension. The resulting d-V_4_C_3_T*_x_* film had a large interlayer spacing of 2.1 nm that allowed for easy ion diffusion, resulting in high capacitance (293 F g^−1^ at 2 mV s^−1^) and retention (86% at 200 mV/s). The asymmetric device assembled with d-V_4_C_3_T*_x_* and VC delivered an ED of 22.2 Wh L^−1^ and PD of 285.3 W L^−1^. The flexible thin-film electrode showed good stability and flexibility, making it suitable for use in flexible supercapacitors and wearable electronic devices. Yang et al. [284] used a temperature-controlled annealing process to create Ni-MOFs/V_2_CT*_x_* nanostructures on nickel foam, leading to the formation of hierarchically porous nanorod nanostructures. The optimized MOF/V_4_C_3_T*_x_*/NF electrode showed the highest capacity of 1104 C g^−1^ at 1 A g^−1^, and the assembled asymmetric device delivered an ED of 46.3 Wh kg^−1^ with a PD of 747 W kg^−1^ and a capacity retention of 118.1% over 15,000 cycles. The study demonstrated the impact of the Ni-O-V bond interface interaction on the charge density distribution.

Zhang et al. [271] proposed a method to create a superior battery-type electrode by self-assembling nickel metal-organic frameworks (Ni MOF) on Ti_3_C_2_T*_x_* MXene nanosheets. The method involved bottom-up self-assembly of Ni MOF amorphous nanospheres on Ti_3_C_2_T*_x_* nanosheets, followed by a process to convert the MOF nanospheres into porous nickel phosphates. This unique structured electrode offered a high capacity of 639 C g^−1^ at 0.5 A g^−1^ with excellent stability, retaining 85% of its capacity after 10,000 cycles. The assembled asymmetric supercapacitor device using this material as an anode and p-phenylenediamine-functional reduced graphene oxide (PPD-rGO) as the cathode delivered a high ED of 72.6 Wh kg^−1^ at a high PD of 932 W kg^−1^, with a capacity retention of 94% after 10,000 cycles. Kshetri et al. [285] proposed a design for a flexible and wearable quasi-solid-state supercapacitor using Co-MOF structures on highly flexible and conductive MXene-carbon nanofiber mats (Co-MOF@MX-CNF). Using Co-MOF@MX-CNF as a starting material, capacitive Co-PC@MX-CNF and battery-type MnO_2_@Co_3_O_4_-PC@MX-CNF electrodes were created for hybrid supercapacitors. The Co-PC@MX-CNF and MnO_2_@Co_3_O_4_-PC@MX-CNF electrodes showed a high capacitance of 426.7 F g^−1^ at 1 A g^−1^, a capacity of 475.4 mAh g^−1^, and good mechanical flexibility. The assembled Co-PC@MX-CNF//MnO_2_@Co_3_O_4_-PC@MX-CNF hybrid device had an operating voltage of up to 1.5 V, an ED of 72.5 Wh kg^−1^, a PD of 832.4 W kg^−1^, and a capacitance retention of 90.36% after 10,000 cycles. The study also showed that two of these devices connected in series were able to power a digital clock and light a green LED bulb, indicating its potential as a power source for wearable devices. Yue et al. [286] created a hierarchical heterostructure electrode for hybrid supercapacitors by in situ stabilizing highly redox-active Ni/Co MOFs on aminated Ti_3_C_2_T*_x_*. The aminated Ti_3_C_2_T*_x_* provides high electrical conductivity and good electrolyte wettability, allowing for uniform stabilization of the Ni/Co MOF and excellent ion transport from the redox-active centers. The Ni/Co-MOF@TCT-NH_2_ electrode showed a high capacitance of 1924 F g^−1^ at 0.5 A g^−1^ and good cycling stability after 10,000 cycles at 10 A g^−1^. The assembled Ni/Co-MOF@TCT-NH_2_//AC asymmetrical supercapacitance had an ED of 98.1 Wh kg^−1^ at 600 W·kg^−1^ and showed a capacitance of 351.3 F·g^−1^ at 0.5 A·g^−1^ with Coulombic efficiency maintained at approximately 99.3% after 15,600 cycles. Nickel-organic framework (Ni-MOF)/Ti_3_C_2_T*_x_* (MXene) hybrid nanosheets as supercapacitor electrode materials were fabricated via a facile ultrasonic method [287]. Ti_3_C_2_T*_x_* nanosheets were uniformly dispersed on the surface of Ni-MOF, thus enhancing the electronic conductivity and preventing the aggregation of Ni-MOF nanosheets. The as-prepared Ni-MOF/MXene hybrid nanosheets exhibited a high specific capacitance of 867.3 F g^−1^ at 1 A g^−1^ and excellent rate capacity.

### 7.6. COFs/MXene Supercapacitors

Covalent organic frameworks (COFs) are a class of porous materials that have gained significant attention in recent years due to their unique properties and potential applications in various fields. COFs are made up of covalently bonded organic molecules, which form highly ordered and crystalline porous structures. The high degree of order and crystallinity in COFs leads to well-defined channels, which provide large surface areas and highly accessible functional sites. One important application of COFs is in the field of energy storage. The large surface areas of COFs can be utilized for the adsorption of ions or molecules, making them ideal for use as supercapacitor electrodes. For example, COFs have been used as electrodes for supercapacitors to achieve high specific capacitances and excellent stability even after thousands of cycles. In the field of catalysis, COFs have been used as highly efficient catalysts due to their well-defined channels and highly accessible functional sites, which enable the effective and selective conversion of chemical reactions. COFs have been used as catalysts in various reactions, such as the hydrolysis of esters, the hydration of nitriles, and the hydroamination of alkenes. In the field of separation, COFs have been used as highly selective and efficient sorbents due to their high surface areas and well-defined channels. COFs have been used for the separation of small molecules, such as gases and liquids, as well as for the separation of larger molecules, such as proteins and enzymes. In fact, COFs are an important class of porous materials due to their highly ordered channels, high surface areas, and highly accessible functional sites. These properties make COFs ideal for a range of scientific and technological applications, including energy storage, catalysis, and separation. For example, Geng et al. [288] developed a covalent organic framework (AQ-COF) supported by amino-modified Ti_3_C_2_ nanosheets using in situ growth. The covalent interactions between the terminal C=O groups of the AQ-COF and the amino units of Ti_3_C_2_ allowed for the uniform dispersal of the COF nanostructures on the Ti_3_C_2_ surface. The COF/Ti_3_C_2_ nanostructure combines the high conductivity of the two-dimensional structure of Ti_3_C_2_ with the porous structure and redox-active groups of AQ-COF, providing a large surface area and optimal porous structure for charge storage. The COF/Ti_3_C_2_ nanostructure showed a capacitance of 290 F g^−1^ at 0.5 A g^−1^ with good rate capability in a Na_2_SO_4_ electrolyte. This is the first report on the heterostructure of COFs and MXene as the electrode for supercapacitors, and this result will motivate further research on such structures.

## 8. Ternary MXene-Based Composites for Supercapacitors

**Figure 20:**
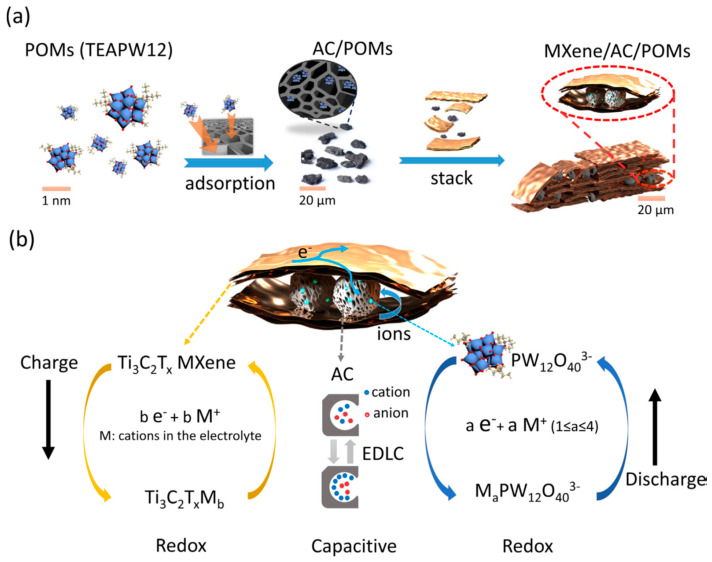
(**a**) Schematic illustration of the combination of TEAPW12, AC, and MXene. (**b**) Schematic illustration of the working mechanism of MXene/AC/TEAPW_12_. Reproduced with permission from [289]. Copyright 2022 Elsevier.

Zhu et al. [289] have reported the development of a ternary electrode material consisting of Ti_3_C_2_T*_x_*/activated carbon/polyoxometalate (POM), which has shown enhanced capacitance for organic-electrolyte supercapacitors (see Figure 20). The study found that among two different types of POMs tested, Ti_3_C_2_T*_x_*/activated carbon/tetraethylammonium phosphotungstate (TEAPW_12_) exhibited the highest gravimetric capacitance of 87 F g^−1^ at 1 mV s^−1^ and stored 91% of the surface capacitance process at 2 mV s^−1^. The capacitance was further increased by 21% after replacing the TEA cation with 1-ethyl-3-methylimidazolium cation (EMIM^+^). The asymmetric device of Ti_3_C_2_T*_x_*/activated carbon/TEAPW_12_ showed 4.6 times higher gravitational energy density and 3.5 times higher volumetric energy density compared to the MXene-based batteries. This study highlights the synergistic effect of ternary MXene-based electrodes and demonstrates that the gravimetric and bulk capacitance can be optimized by selecting the right POM with optimized concentrations.

Wang et al. [290] developed a ternary electrode material composed of MXene, reduced graphene oxide (rGO), and polypyrrole (PPy) nanostructures, which formed flexible thin films. The resulting MXene/rGO-PPy nanostructures showed high electrical conductivity, mechanical durability, and pseudocapacitance. The MXene/rGO-PPy nanostructure achieved the highest capacitance of 408.2 F g^−1^ and a capacitance retention of 67.3% at 10 A g^−1^. The assembled asymmetric device delivered an ED of 11.3 Wh kg^−1^ at a PD of 500 W kg^−1^ and maintained a capacitance retention of 91.2% after 10,000 cycles, demonstrating its potential for use in flexible energy storage devices with high volume-gravimetric energy density. The addition of carbon and MXene to N-doped MnO_2_ was found to improve the performance of supercapacitors [291]. The N-doped MnO_2_/MXene electrode showed a high capacitance of 457 F g^−1^ at 1 A g^−1^ with a capacitance retention of 102.5% after 1000 cycles due to the favorable interaction between MnO_2_ and MXene. Taking advantage of the fact that V has multiple oxidation states that allow V_2_CT*_x_* to participate in more redox reactions, Zhang et al. [292] fully exploited the electrochemical properties of V_2_CT*_x_*, constructing a sandwich-like MXene V_2_CT*_x_*/Ag/rGO/MWCNTs layered nanocomposite. The doping of Ag nanoparticles, rGO, and MWCNTs prevented the collapse and accumulation of V_2_CT*_x_* and improved the interlayer spacing, leading to a capacitance contribution rate of the composite of 86.6%.

Wang et al. [293] developed a 3D Ti_3_C_2_T*_x_*/rGO-Fe nanostructured electrode using an ion-induced gelation strategy. The resulting hydrogel had a high surface area and provided a high capacitance of 3194 mF cm^−2^ at 1 mA cm^−2^. The assembled device showed a high ED (76.3 μWh cm^−2^) and capacitance retention (95.6% after 5000 cycles at 6 mA cm^−2^) due to the 3D porous structure of the Ti_3_C_2_T*_x_*/rGO-Fe hydrogel nanostructures. Guo et al. [294] developed a ternary nanostructure for use as a supercapacitor electrode. The structure consisted of in situ-grown Ni-MOF on porous conductive Ti_3_C_2_T*_x_*. Then, the precursor p-MXene/Ni-MOF was transformed into a newly porous material p-MXene@Ni_3_S_4_/CuS accompanied by the synthesis of CuS. The final nanostructured electrode exhibited a specific capacitance of 1917 F g^−1^ and a good cycle performance of 91.2% over 30,000 cycles. The assembled electrode showed the highest ED of 87.62 Wh kg^−1^ at a PD of 775 W kg^−1^. The improved performance was attributed to the porous Ti_3_C_2_T*_x_* facilitating electron transport and preventing nanoparticle agglomeration, the second phase of CuS enhancing the electronic properties, Ni_3_S_4_ and CuS exposing more active sites, and the ternary structure producing a synergistic effect.

Wang et al. [295] reported the synthesis of Ti_3_C_2_T*_x_*-wrapped V_2_O_5_/Fe_2_O_3_ (Ti_3_C_2_T*_x_*@VFO) nanostructures using a hydrothermal method. The VFO nanostructures on carbon cloth showed a capacitance of 435.2 mF cm^−2^ at 2 mA cm^−2^. The Ti_3_C_2_T*_x_*@VFO nanostructure showed an increased capacitance of 1150.82 mF cm^−2^ at 2 mA cm^−2^ due to the improved conductivity and reduced collapse and shedding of VFO during the electrochemical reaction. The asymmetric device consisting of Ti_3_C_2_T*_x_*@VFO//MnO_2_ had an ED of 0.17 mWh cm^−2^ at a PD of 1260 W cm^−2^ with a capacitance retention of 74.92% over 5000 cycles. Li et al. [296] developed a conductive and free-standing film of Ti_3_C_2_T_z_/CNT@MnO_2_ (TCM) for use in aqueous supercapacitors through the electrostatic ordered assembly. The film showed a strong coupling between high-energy delta-MnO_2_ and Ti_3_C_2_T_z_, providing numerous active sites for electrolyte ions. The TCM electrode had a galvanometric capacitance of 384 F g^−1^ and bulk capacitance of 577 F cm^−3^ at 0.5 A g^−1^, with a 61% retention rate at 50 A g^−1^ and 92.2% capacitance retention. The assembled asymmetric TCM/N-doped rGO delivered an ED of 44 Wh kg^−1^ and a PD of 43,4 kW kg^−1^ in 1 mol L^−1^ Na_2_SO_4_. Chen et al. [297] developed a high-performance supercapacitor using freestanding V_4_C_3_T*_x_*@NiO-reduced graphene oxide (rGO) core-shell hierarchical heterostructured hydrogel electrode. The NiO nanoflowers were grown on the surface of V_4_C_3_T*_x_* to form a core-shell nanostructure, which was then incorporated into a 3D interconnected porous hydrogel. The V_4_C_3_T*_x_*@NiO-rGO electrodes showed a specific capacitance of 1009.5 F g^−1^ at 1 A g^−1^, higher than that of the V_4_C_3_T*_x_*@NiO nanostructure (665.3 F g^−1^) and V_4_C_3_T*_x_* (184 F g^−1^), and a good cycle stability of 97.4% after 10,000 cycles at 10 A g^−1^. The symmetric device made from these electrodes delivered an ED of 61.13 Wh kg^−1^ at a PD of 526.3 W kg^−1^ with a capacitance retention of 97.4% over 10,000 cycles at 10 A g^−1^. The 3D hydrogel structure also provides remarkable mechanical strength, eliminating the need for binders in the electrode materials.

Bai et al. [298] developed Ti_3_C_2_-Cu/Co nanostructures through molten salt etching and found that the presence of metal atoms and their interactions with Ti_3_C_2_ through surface O atoms improved the electrochemical activity of the Ti_3_C_2_-Cu electrode. This electrode showed a pseudocapacitive contribution from Cu and a high specific capacitance of 885 F g^−1^ at 0.5 A g^−1^ in 1 mol L^−1^ H_2_SO_4_. The symmetric supercapacitor made from these electrodes had an operating voltage of 1.6 V, an areal capacitance of 290.5 mF cm^−2^ at 1 mA cm^−2^, and a cycle stability of 89% over 10,000 cycles. The device delivered an ED of 103.3 Wh cm^−2^ at a PD of 800 W cm^−2^, which was attributed to the unique intercalation structure and the synergistic effect between Ti_3_C_2_ and Cu. Wu et al. [299] reported a novel method for the fabrication of a flexible free-standing polyaniline@Ti_3_C_2_T*_x_*-CNTs nanostructured film for use as a supercapacitor electrode. The method involves a solvent-assisted self-assembly process and a vacuum filtration process to produce a ternary structure of polyaniline (0D), Ti_3_C_2_T*_x_* (2D), and CNTs (1D), resulting in a reinforced concrete structure. This film exhibited good mechanical properties, including a tensile strength of 99.4 MPa and a peak capacitance of 463 F g^−1^ at 5 mV s^−1^ with a capacitance retention of 92% after 10,000 cycles. The asymmetric device made from this cathode and a free-standing Ti_3_C_2_T*_x_*-CNT anode delivered an ED of 10 Wh g^−1^ at a PD of 2808 W kg^−1^.

Let us recall the flexible supercapacitor using Ti_3_C_2_T*_x_*/carbon nanotube/porous carbon film fabricated by Yang et al. [263]. The problem of irregular porous carbon leading to low electron transport efficiency and fragile behavior was solved by introducing CNTs to build a highly conductive network structure. This structure anchored the porous carbon to the Ti_3_C_2_T*_x_* flakes, improving the electron transport and increasing the contact area between the Ti_3_C_2_T*_x_* and porous carbon. The CNT/porous carbon/Ti_3_C_2_T*_x_* nanostructured electrode provided a high capacitance of 365 mF cm^−2^, and the assembled flexible symmetric device delivered an ED of 10.5 Wh cm^−2^ at a PD of 30 W cm^−2^, with 75% capacity retention at 50 mA cm^−2^. This approach offers a solution to overcome the self-recombination phenomenon in Ti_3_C_2_T*_x_* thin films while maintaining electrical conductivity and flexibility with a large charge storage capacity and high-rate capability. Li et al. [300] developed a method to improve the capacitive activity and stability of Ti_3_C_2_T*_x_* thin film electrodes for supercapacitors. The method involved preparing Ti_3_C_2_T*_x_* gels through an alkali-induced process and introducing carbon nanotubes during the treatment process. This resulted in a porous structure with an improved surface area and enhanced ion/electron transport channels, leading to a high capacitance of 401.4 F g^−1^ at 1 A g^−1^, a good rate capability of 336.2 F g^−1^ at 1000 A g^−1^, and stable cycling with 99% retention after 20,000 cycles at 100 A g^−1^, making the Ti_3_C_2_T*_x_*/CNT film a promising electrode material for energy storage devices. Damiani et al. [301] developed a method for fabricating electrodes for flexible microsupercapacitors using a combination of Ni(OH)_2_-Ni-Ti_3_C_2_ films on copper wires and a porous cauliflower-like Ni-Ti_3_C_2_ film as a supporting scaffold. The unique nanostructured electrode had a high capacitance of 1725.23 F cm^−3^ and a rate capability of 929.23 F cm^−3^, which was attributed to its cauliflower-like morphology and high conductivity of Ti_3_C_2_. The assembled fiber-like hybrid device had a high capacitance of 72.35 F cm^−3^ and ED of 206 μWh cm^−2^ and showed excellent flexibility with a long-cycle stability of 89.3% after 7000 cycles.

Sree Raj et al. [302] developed CrSe_2_/Ti_3_C_2_ hybrid nanostructured electrodes using a hydrothermal method. The electrode showed an areal capacitance of 133 mF cm^−2^ at 2 mA cm^−2^, with 81.25% retention after 5000 cycles at 10 mA cm^−2^ and 100% Coulombic efficiency. The symmetric CrSe_2_/Ti_3_C_2_ device had an ED of 7.11 μWh cm^−2^ at a PD of 355 μW cm^−2^, with 82% capacitance retention and 100% Coulombic efficiency over 5000 cycles, demonstrating excellent reversibility.

Li et al. [303] developed a flexible supercapacitor electrode using a sandwich-like film composed of Ti_3_C_2_T*_x_*, α-Fe_2_O_3_-C-MoS_2_-PEDOT:PSS (FMP), and a carbon cloth. The FMP was used to improve the areal capacitance of the Ti_3_C_2_T*_x_* material. The flexible supercapacitor showed a high areal capacitance of 2.7 F cm^−2^ (541 F g^−1^) and a high ED and PD of 371 Wh cm^−2^ and 12.36 Wh kg^−1^, respectively. The device also showed excellent flexibility and a high power density of 760.32 W kg^−1^.

## 9. Printed MXene Supercapacitors

Printed MXene-based supercapacitors are a new and promising technology in the field of energy storage [9,20,304,305,306,307,308,309,310,311,312,313,314,315]. They offer numerous advantages over traditional capacitors and batteries, including high energy density, fast charge and discharge rates, and a long cycle life. Additionally, the ability to print these supercapacitors on a large scale makes them a cost-effective solution for a variety of applications, ranging from portable electronics to grid-scale energy storage. The development of printed MXene-based supercapacitors has seen significant progress in recent years. This is largely due to the unique properties of MXenes, which are a new class of 2D transition metal carbides and nitrides. MXenes have high electrical conductivity, high surface area, and excellent chemical stability, making them ideal for use in energy storage devices. One of the key advantages of printed MXene supercapacitors is their high energy density. This allows them to store much more energy per unit volume than traditional capacitors, making them an ideal solution for high-power applications such as backup power for data centers or renewable energy storage. Another advantage of printed MXene supercapacitors is their fast charge and discharge rates. This means that they can store and release large amounts of energy quickly, making them well-suited for applications that require fast bursts of power, such as starting a car or powering an electric vehicle. For example, in 2022, a research team from Tsinghua University in China developed a printed MXene supercapacitor that could be charged and discharged in just a few seconds [311]. Printed MXene supercapacitors also have an excellent cycle life, meaning that they can be charged and discharged many times without degrading the performance of the device. This makes them an ideal solution for applications where the energy storage device needs to last for a long time, such as in grid-scale energy storage systems.

As a second example, Zhang et al. [145] demonstrate the promising potential of in-plane hybrid supercapacitors (IHSC) on textiles as effective and printable power sources used in flexible and wearable electronics. The battery-type electrode is incorporated by combining the high capacity of metallic layer double hydroxide (NiCoAl-LDH), the high conductivity of Ti_3_C_2_T*_x_* MXene and Ag nanowires, with the skeleton function of Ti_3_C_2_T*_x_* MXene, which displays a high capacity of 592 C g^−1^ at 1 A g^−1^, high-rate performance, and long cycle life over 10,000 cycles. Based on this composite material and active carbon (negative electrode), a screen-printed IHSC device on textile presents a high areal ED of 22.18 μWh cm^−2^ and PD of 3.0 mW cm^−2^. In addition to these advantages, printed MXene supercapacitors can also be produced at a low cost, making them an attractive option for a wide range of applications. The ability to print these supercapacitors on large sheets of conductive material using simple, scalable processes, such as screen printing or inkjet printing, reduces the cost of production compared to traditional energy storage devices. In conclusion, printed MXene-based supercapacitors have numerous advantages over traditional capacitors and batteries, making them a promising technology for a wide range of energy storage applications. Their high energy density, fast charge and discharge rates, excellent cycle life, and low cost of production make them an attractive solution for both portable electronics and grid-scale energy storage. With continued progress in research and development, it is likely that printed MXene supercapacitors will play an increasingly important role in meeting the world’s growing energy storage needs in the coming years.

**Figure 21:**
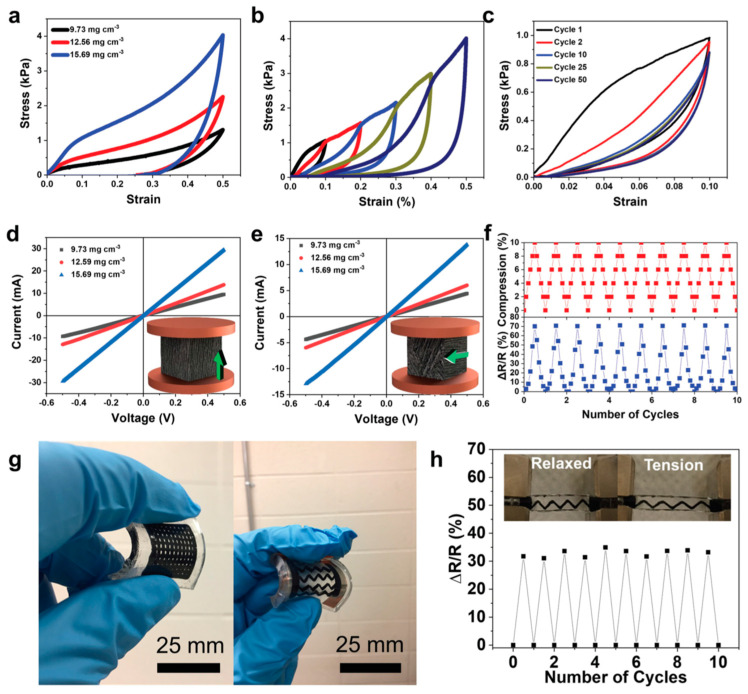
Mechanical and electrical properties of 3DFP MXene aerogels. (**a**) Stress-strain plots of the aerogels having different densities after uniaxial compression tests up to 50% compressive strain. (**b**) Stress-strain curves of multicycle compression by increasing the strain amplitude of printed MXene aerogels (*ρ* = 15.69 mg cm^−3^). (**c**) Stress-strain curves for 50 loading-unloading cycles with up to 10% strain (*ρ* = 15.69 mg cm^−3^). (**d**) I–V curves of Ti_3_C_2_T*_x_* aerogels with different densities parallel to the freezing direction. (**e**) I–V curves of Ti_3_C_2_T*_x_* aerogels with different densities perpendicular to the freezing direction. The arrows in the inset of both (**d**,**e**) indicate the freezing direction. (**f**) Response in the aerogel resistance to compression with 10% strain for 10 consecutive cycles (*ρ* = 15.69 mg cm^−3^). (**g**) 3D freeze-printed MXene aerogels infiltrated in the PDMS elastomer. (**h**) Response in the resistance of the 3D freeze-printed aerogels infiltrated in PDMS after applying 10% tension. Reproduced with permission from [309]. Copyright 2022 Wiley.

Additive-free, 2D Ti_3_C_2_T*_x_* MXene aqueous inks with appropriate rheological properties for scalable screen printing were reported by Abdolhosseinzadeh et al. [305]. The printed resilient microsupercapacitors (MSCs) demonstrated an excellent charge storage performance of 158 mF cm^−2^ at 0.08 mA cm^−2^, which outperforms the areal capacitance and energy density of MSCs based on laser-scribed d-Ti_3_C_2_T*_x_* (24–27 mF cm^−2^) [312,313]. Tetik et al. [309] fabricated ultra-light and true 3D Ti_3_C_2_T*_x_* aerogel structures using 3D cryo-printing technology (Figure 21). This process combines unidirectional cryocasting and inkjet printing to tailor the micro and macrostructure of the aerogel. The technique does not require viscoelastic shear-thinning inks, and ice is used as a support material to develop true 3D structures. The aerogel exhibits good electromechanical properties and retains its conductivity through successive cycles of compression. The interdigitated electrode provides an ED of 2 μWh cm^−2^ at a PD of 6 kW cm^−2^. Zhou et al. [293] developed a 3D-printed solid-state supercapacitor using MXene-based ink and cellulose nanofiber (CNF) materials. The ink was formulated by controlling the oxidant content and using 2,2,6,6-tetramethylpiperidin-1-oxyl (TEMPO) as a rheology modifier. The 3D-printed supercapacitor achieved an areal capacitance of 2.02 F cm^−2^ and an ED of 101 μWh cm^−2^ at a PD of 299 W cm^−2^. The device was able to maintain 85% of its performance after 5000 cycles. The authors suggest that further optimization of the electrode structure, electrolyte, and active materials could increase the energy and power density of this type of device. Table 3 lists the characteristics of some printed MXene-based supercapacitors. Yang et al. [311] fabricated additive-free 3D architected MXene aerogels via a 3D printed template-assisted method that combines a 3D printed hollow template and a cation-induced gelation process. This method allows the use of MXene ink with a wide range of concentrations (5 to 150 mg mL^−1^) to produce Ti_3_C_2_T*_x_* MXene aerogels with high structural freedom, fine feature size (>50 μm), and controllable density (3 to 140 mg cm^−3^). Through structure optimization, the 3D Ti_3_C_2_T*_x_* MXene aerogel shows a high areal capacitance of 7.5 F cm^−2^ at 0.5 mA cm^−2^ with a high mass loading of 54.1 mg cm^−2^. It also exhibits an ultrahigh areal ED of 0.38 mWh cm^−2^ at a PD of 0.66 mW cm^−2^.

**Table 3:** Characteristics of printed MXene-based supercapacitors.

MXene Material	Substrate	Capacity/Capacitance	Stability	Ref.
Ti_3_C_2_T*_x_*	Glass slide	242 F g^−1^@0.2 A g^−1^	90%@10,000	[20]
Ti_3_C_2_T*_x_* nanosheets	Flexible plastic	562 F cm^−2^@0.08 mA cm^−2^	100%@10	[304]
Ti_3_C_2_T*_x_*	Glossy photo paper	158 mF cm^−2^	95.8%@17,000	[305]
Ti_3_C_2_@_3_D nanocarbon	Nanocarbon disk	194 mF g^−1^@ 10 µA	CE = 66%	[306]
Ti_3_C_2_T*_x_*	Textile/paper	294 mF cm^−2^@2 mV s^−1^	-	[307]
Ti_3_C_2_T*_x_*	Glass	2.02 F cm^−2^@1 mA cm^−2^	85%@5000	[308]
Ti_3_C_2_T*_x_* aerogels	Paper	200 F g^−1^ at 2 mV s^−1^	94%@10,000	[309]
N@ZnCoSe_2_-Ti_3_C_2_T*_x_*	Carbon fibers	19.36 F g^−1^	83.7%@6000	[310]
Ti_3_C_2_T*_x_* aerogels	-	7.5 F cm^−2^@0.5 mA cm^−2^	97.1%@10,000	[311]
NiCoAl-Ag-Ti_3_C_2_T*_x_*	Polyester-cotton	592 C g^−1^@1 A g^−1^	80%@10,000	[145]

## 10. Advanced Technologies

Novel technologies open up new possibilities for the application of Ti_3_C_2_T*_x_*-based materials in self-powered electronics. For example, Xu et al. [314] reported the development of a thermally chargeable supercapacitor using 3D Ti_3_C_2_T*_x_* MXene hollow spheres as freestanding electrodes. They used a filtration and annealing process to convert the 2D Ti_3_C_2_T*_x_* into 3D hollow structures. The resulting device had a high feedback coefficient of 78.4 mV kg^−1^ and a stable output voltage of 400.6 mV under a temperature difference of 5.8 K and could be used to power self-powered integrated electronics when four devices were connected in series. The concept of thermally chargeable supercapacitors, also known as thermal energy storage supercapacitors, has gained much attention in recent times. This type of supercapacitor is unique in the sense that it can be charged using heat energy from various sources such as solar, waste heat, and geothermal, unlike traditional supercapacitors, which require external electrical energy for charging. This feature makes thermally chargeable supercapacitors a promising technology for integrating renewable energy into the electrical grid, as well as for other applications such as energy harvesting and thermoelectric power generation. One of the key benefits of these supercapacitors is their high efficiency in storing energy, along with their rapid charging and discharging times. This makes them ideal for applications that require quick and reliable energy storage and retrieval, such as wearable electronics, energy harvesting devices, and grid-level energy storage. Furthermore, thermally chargeable supercapacitors are highly durable and have a long lifespan, which makes them a low-maintenance solution for energy storage. Another advantage of these supercapacitors is their ability to operate in a wide range of temperatures, making them suitable for use in harsh and extreme environments. Unlike traditional batteries, which have limited temperature ranges and can degrade quickly in high-temperature environments, thermally chargeable supercapacitors can perform optimally in temperatures ranging from sub-zero to over 200 °C. This makes them ideal for a range of industrial, military, and medical applications. Moreover, thermally chargeable supercapacitors are environmentally friendly and have a low environmental impact. They do not contain toxic or hazardous materials and can be easily recycled, making them a safe and sustainable alternative to traditional energy storage solutions. Additionally, they can be manufactured using low-cost and scalable processes, making them accessible and economically viable for a wide range of applications. Recently, there has been much progress in the development of thermally chargeable supercapacitors, with many new materials and designs being explored to improve energy density, stability, and performance. For instance, researchers have explored the use of graphene and MXene materials as electrodes due to their high thermal and electrical conductivity. There have also been advancements in the use of phase change materials, metal-organic frameworks, and nanoparticles to enhance the performance and stability of these supercapacitors. In fact, the significance and necessity of thermally chargeable supercapacitors cannot be overemphasized. With their ability to store energy efficiently, operate in a wide range of temperatures, be environmentally sustainable, and be economically viable, they hold the potential to transform energy storage and provide a new, sustainable, and renewable solution. Zhang et al. [315] reported the improvement of K-ion storage activity in Ti_3_C_2_T*_x_* anodes through building 3D structures and electrolyte optimization. 3D foam-like scaffolds were developed through electrostatic neutralization reactions with melamine and were found to effectively improve the electrolytic accessibility and shorten the K^+^ diffusion path of Ti_3_C_2_T*_x_*. KFSI (potassium bis(fluorosulfonyl)imide) was found to be more effective than potassium hexafluorophosphoric acid, KPF_6_, in maintaining cycle stability as a salt in a non-aqueous carbonate solvent. The 3D foam architecture of Ti_3_C_2_T*_x_* scaffolds and KFSI-based electrolytes resulted in an electrochemical capacitance of 161.4 mAh g^−1^ at 30 mA g^−1^ and 100% Coulombic efficiency over 2000 cycles. The asymmetric potassium-ion capacitor consisting of 3D-FMS as the anode and alternating current as the cathode showed an ED of 57 Wh kg^−1^ at a PD of 5985 W kg^−1^ with 95% capacitance retention over 10,000 cycles at a voltage range of 0.01–4 V. This study demonstrates the effectiveness of the 3D architecture and KFSI-based electrolytes in enhancing the K-ion storage capacity in Ti_3_C_2_T*_x_* electrodes and may lead to further development in MXene-based electrodes for K storage systems.

Another new technology is related to the piezoelectric supercapacitor (PSC). For example, Jadhav et al. [316] reported a novel Ti_3_C_2_T*_x_*-based PSC that has excellent mechanical energy harvesting and energy storage properties. The PSC is made from Ti_3_C_2_T*_x_* multilayer plates combined with a piezoelectric separator made of PVDF and a polymer gel electrolyte. The symmetric PSC showed a capacitance of 61 mF cm^−2^ and an ED of 25 mJ cm^−2^ at a PD of 1300 W cm^−3^. The PSC was found to have good stability after 4500 cycles. Yuan et al. [317] report a method of using femtosecond laser ablation to synthesize Ti_3_C_2_T*_x_* quantum dots (QDs), and laser-reduced graphene oxide (LRGO) is described. The resulting MQD/LRGO transparent composite electrodes have a high transparency of over 90% and good electrochemical activity. These electrodes were used to make flexible, transparent supercapacitors that exhibit an ED of 2.04 × 10^−3^ μWh cm^−2^, a PD of 129.4 μW cm^−2^, and a retention of 97.6% after 12,000 cycles.

## 11. Concluding Remarks

The current developments and successes of new 2D/2D MXene layered heterostructures for cutting-edge research on cutting-edge MXene-based supercapacitor applications are discussed in this paper. Supported by its simple synthesis techniques, distinctive combination, and customizable nature of attributes for individual applications, MXene has become an inescapable hotspot in the field of energy storage and supercapacitors, particularly over the past ten years. The idea of adding complementary potential 2D materials, which not only boost electrochemical activity but also give a coexisting high surface area, avoiding restacking difficulties and higher conductivity, was realized by an urgent need in structural and morphological augmentation. Additionally, these heterostructured frameworks enhance overall interfacial structural and electrochemical stability while inducing individual energy storage contributions and exerting synergistic interfacial functions. It is intriguing how combining several 2D structures into MXene can use a special combination of bulk and interfacial features for the heterostructure. Most heterostructures were often electrostatically self-assembled by physical mixing or vacuum-assisted filtering for the creation of the 2D/2D composite or films because of the significant oxidation vulnerability of MXene under extreme circumstances and long-term exposure to ambient conditions. While the production of heterostructures and their accompanying attributes are highlighted, approaches including hydrothermal treatment, in situ chemical growth, or reduction under controlled environments have also been effectively used. First, MXene/rGO and the related ternary nanocomposites have drawn the greatest attention, with most of these composites using in situ GO reduction in MXene nanosheet templates for MXene/rGO synthesis.

This review thoroughly covers synthesis methods and characterization techniques. An example of an outstanding result is a flexible and free-standing modified MXene/holey graphene film by filtration of the alkalized MXene and holey graphene oxide dispersions, followed by a mild annealing treatment [318]. After the terminal groups (-F/-OH) are removed, this film used as electrode materials for supercapacitors can deliver an ultrahigh volumetric capacitance (1445 F cm^−3^) at 2 mV s^−1^, and still retain an ultrahigh volumetric capacitance (988 F cm^−3^) when the mass loading is increased to 12.6 mg cm^−2^, which is significantly superior to many known electrode materials. The assembled symmetric supercapacitor demonstrates a fantastic volumetric energy density (38.6 Wh L^−1^). Electrodeposited Ni-Co layered double hydroxides on titanium carbide provide a capability of 983.6 F g^−1^ at 2 A g^−1^ and 536.6 F g^−1^ at 50 A g^−1^ and cycling stability with 76% retention after 5000 cycles at 30 A g^−1^ [319]. Gravimetric and volumetric capacitances as high as 503 F g^−1^ and 1682 F cm^−3^, with a capacitance retention of 98.3% after 10,000 cycles, were reported for MXene/PANI electrodes [320]. Carbon-intercalated Ti_3_C_2_T*_x_* displayed a high reversible gravimetric capacitance of 364.3 F g^−1^ at a current density of 1 A g^−1^ with above 99% retention over 10,000 cycles [321]; Shang et al. reported a gelation method to prepare a 3D structured hydrogel from 2D MXene sheets that was assisted by graphene oxide and a suitable reductant. The hydrogel delivered a capacitance up to 370 F g^−1^ at 5 A g^−1^ for 10,000 cycles and a capacity of 165 F g^−1^ at 1,000 A g^−1^ [255]. Fan et al. fabricated a compact and nanoporous MXene film with a folded structure by mechanically pressing a three-dimensional MXene aerogel. It retained a high volumetric capacitance (616 F cm^−3^) when the mass loading reached 12.2 mg cm^−2^. The fabricated symmetric supercapacitor had a good volumetric energy density of 14.1 Wh L^−1^, demonstrating enormous potential in practical applications [322]. Chen et al. obtained a 3D porous structure of Ti_3_C_2_T*_x_* by adding alkali to a Ti_3_C_2_T*_x_* colloid, which was followed by flocculation. The sample showed an excellent specific capacitance of approximately 400.7 F g^−1^ at a current density of 1 A g^−1^, with a capacitance retention of 89% after 5000 charge-discharge cycles [323]. Zhang et al. fabricated a 3D macroporous MXenes film and aerogel using liquid nitrogen rapid freezing. The capacitance reached 372 F g^−1^ (1355 F cm^−3^) for the film and 404 F g^−1^ (1293 F cm^−3^) for the aerogel at a current density of 1 A g^−1^. The supercapacitor constructed using the film had a volumetric energy density of 32.2 Wh L^−1^ at a power density of 946 W L^−1^ [324]. These examples illustrate the enormous potential of MXenes.

The majority of MXene composites are HF etched or in situ HF etched through an acid/salt reaction, despite the fact that the heterostructure production procedures are sufficiently precise to extract the most benefits from the materials. It appears that these methods lead to MXene having an excessive amount of fluorine surface terminations, which reduces its true capacitance and structure and highlights the significance of synthesizing MXene using the previously discussed fluorine-free methods, particularly the bottom-up method known as CVD for high-purity MXene with high electrochemical throughput. Furthermore, the majority of 2D heterostructures were composed of Ti_3_C_2_T*_x_*, with very few experiments on Ti_3_C_2_T*_x_*, V_4_C_3_T*_x_*, and Nb_2_CT*_x_*-based nanocomposites. This is because 70 distinct MXenes have been theoretically established, and almost 30 MXenes have been actually synthesized. Therefore, it is anticipated that various metal-composed or mixed-metal MXenes will display a reassuringly diverse collection of features, opening up new research opportunities on MXene heterostructured nanocomposites with appealing energy storage properties.

Given the variety of heterostructures reported with various compositions, a fundamental investigation into the relationship between the structure, composition, and performance of the MXene 2D materials becomes crucial for developing an interfacial nanocomposite that is suitable for supercapacitor application. In order to discover and improve the underlying principle for charge storage at the nanoscopic level, this also unquestionably calls for basic theoretical simulation and modeling to predict the 2D-2D unraveled active surface areas possessing a higher electrode–electrolyte interface, the influence of surface termination groups, and its intrinsic charge storage mechanism. The need for such investigations is further supported by specific studies that have already used in situ Raman spectroscopy to comprehend the regulating charge storage properties.

Last but not least, the true potential of 2D materials and heterostructures is sufficiently great to provide a solid foundation for understanding the dynamics of interaction and charge storage of other 2D structures like covalent organic frameworks, MOFs, MOenes, h-BN, etc., with MXene for heterostructure formation in the search for the best supercapacitor electrode material. These 2D/2D heterostructures, which have been confirmed using robust material electrochemistry, may be further translated into device configurations like strong and high-performance wire, flexible and microsupercapacitors for portable and wearable electronics with better device compatibility, as has already been investigated for some nanocomposites with discernible performance. As a result, 2D/2D MXene heterostructures hold enormous potential as the best supercapacitor electrode material for upcoming advanced energy storage applications. The current state of MXene research includes maintaining a low concentration of HF etching in the reaction vessel and accurate control of all synthetic parameters throughout the manufacturing process. Ti_2_AlC and Ti_3_AlC_2_ are commercially produced in hundreds of kilograms at low cost. Large quantities of films (1-m long, ~1-µm thick) have been successfully fabricated using the blade coating technique.

Regarding the impact of the synthesis of MXenes on the environment, health, and sustainability, technical controls must be carried out to minimize or eliminate exposure to HF. Also, thermal runaway caused by exothermic reactions must be eliminated during the initial mixing of the MXene precursors with the etchant. Recently, Liu et al. [325] opened future opportunities, anticipating the development of scalable Lewis acid molten salt etching for the production of MXenes that cannot be made through HF etching, such as nitride MXenes and MXenes with new T*_x_* groups.

The potential and future scope of MXene materials in energy storage are highly promising, as evidenced by recent research on their use in potassium-ion capacitors and supercapacitors. The results of these studies have shown that MXene-based devices have excellent energy storage capabilities and high performance, making them a promising solution for various applications, such as electric vehicles and grid stability. To continue improving the performance of the MXene-based energy storage devices, future research could focus on enhancing their stability and cycle performance, for example, by investigating new electrolyte systems or exploring the use of other MXene materials in combination with Ti_3_C_2_T*_x_*. There is also significant potential for the development of flexible and transparent energy storage devices using MXene materials. Already, transparent supercapacitors based on MXene quantum dots and graphene have been developed, displaying high transparency and electrochemical activity. These devices could have numerous applications in wearable technology and flexible displays. Finally, scaling up the production of MXene materials and integrating them into real-world applications is another promising avenue for future research. MXene-based supercapacitors, for example, could be used in portable electronics and electric vehicles, while potassium-ion capacitors could play a role in large-scale energy storage systems [315,326]. In fact, the prospects for MXene-based energy storage devices are highly promising, and continued research and development in this field may lead to the creation of new and high-performance energy storage solutions. Finally, we believe that collaborative efforts or interdisciplinary approaches (material science, solid-state chemistry, electrochemistry, and many more) are requested to stimulate further advancements in MXene-based supercapacitors. For instance, as the electrolyte is an essential component of supercapacitors, it is noteworthy for the advanced application of flexible supercapacitors that designing MXene material and suitable solid electrolyte/MXene interfaces is a high breakthrough approach.

## Figures and Tables

**Table 1:** Characteristics of MXene electrode materials for zinc-ion supercapacitors.

Material	Electrolyte	Capacitance/Capacity	Cycle Stability	Ref.
Zn-Ti_3_C_2_	ZnSO_4_ gel	130 F g^−1^@1 A g^−1^	82.5%@1000	[197]
Mo_1.33_CT_z_@Ti_3_C_2_T_z_	3 M Zn(CF_3_SO_3_)_2_	105 mAh g^−1^@1 A g^−1^	90%@8000	[198]
Ti_3_C_2_T_z_	0.1 M ZnSO_4_	86 mAh g^−1^@20 mA g^−1^	99%@1000	[202]
Ti_3_C_2_T_z_	2 M ZnSO_4_	124 F g^−1^@0.2 A g^−1^	85%@10,000	[203]
Ti_3_C_2_T_z_ hydrogel	ZnSO_4_	349 F g^−1^@5 A g^−1^	97.1%@100,000	[204]
Ti_3_C_2_T_z_@cotton	ZnSO_4_/MnSO_4_	125 F g^−1^@1 mV s^−1^	80.7%@16,000	[205]
Ti_3_C_2_T*_x_*/Bi_2_S_3_@N-C	Zn(CF_3_SO_3_)_2_	150 F g^−1^@1 A g^−1^	81%@2000	[206]
Mo_1.33_CT_z_@RGO	2 M ZnSO_4_ gel	348 F g^−1^@5 mV s^−1^	81%@10,000	[207]
Ti_3_C_2_T*_x_*	ZnSO_4_/PAM	129 mF cm^−2^@ 0.34 mA cm^−2^	77%@10,000	[208]
H-Ti_3_C_2_T*_x_* film	2 M Zn(CF_3_SO_3_)_2_	105 mAh g^−1^@0.2 A g^−1^	90.8%@20,000	[210]
V_2_CT*_x_*	ZnSO_4_ gel	54.1 mF cm^−2^@0.1 mA cm^−2^	81.5%@8000	[211]
Ti_1.1_V_0.7_Cr_x_Nb_1.0_Ta_0.6_C_3_T_z_	3 M Zn(CF_3_SO_3_)_2_	77 mAh g^−1^@0.5 A g^−1^	87%@10,000	[213]

## Data Availability

Where no new data were created.
